# Regulated Capture of V_κ_ Gene Topologically Associating Domains by Transcription Factories

**DOI:** 10.1016/j.celrep.2018.07.091

**Published:** 2018-08-28

**Authors:** Sophiya Karki, Domenick E. Kennedy, Kaitlin Mclean, Adrian T. Grzybowski, Mark Maienschein-Cline, Shiladitya Banerjee, Heping Xu, Elizabeth Davis, Malay Mandal, Christine Labno, Sarah E. Powers, Michelle M. Le Beau, Aaron R. Dinner, Harinder Singh, Alexander J. Ruthenburg, Marcus R. Clark

**Affiliations:** 1 Department of Medicine, Section of Rheumatology and The Knapp Center for Lupus and Immunology Research, University of Chicago, Chicago, IL, USA; 2 Department of Molecular Genetics and Cell Biology and Department of Biochemistry and Molecular Biology, University of Chicago, Chicago, IL, USA; 3 Research Resource Center, University of Illinois at Chicago, Chicago, IL, USA; 4 Department of Physics & Astronomy, University College London, London WC1E6BT, UK; 5 Division of Immunobiology, Department of Pediatrics, University of Cincinnati, Cincinnati, OH, USA; 6 Section of Hematology/Oncology, University of Chicago, Chicago, IL, USA; 7 Integrated Light Microscopy Core Facility, University of Chicago, Chicago, IL, USA; 8 Department of Biology, Lewis University, Romeoville, IL, USA; 9 James Frank Institute, University of Chicago, Chicago, IL, USA; 10 Lead Contact

## Abstract

Expression of vast repertoires of antigen receptors by lymphocytes, with each cell expressing a single receptor, requires stochastic activation of individual variable (V) genes for transcription and recombination. How this occurs remains unknown. Using single-cell RNA sequencing (scRNA-seq) and allelic variation, we show that individual pre-B cells monoallelically transcribe divergent arrays of V_κ_ genes, thereby opening stochastic repertoires for subsequent V_κ_-J_κ_ recombination. Transcription occurs upon translocation of V_κ_ genes to RNA polymerase II arrayed on the nuclear matrix in transcription factories. Transcription is anchored by CTCF-bound sites or E2A-loaded V_κ_ promotors and continues over large genomic distances delimited only by topological associating domains (TADs). Prior to their monoallelic activation, V_κ_ loci are transcriptionally repressed by cyclin D3, which prevents capture of V_κ_ gene containing TADs by transcription factories. Cyclin D3 also represses protocadherin, olfactory, and other monoallelically expressed genes, suggesting a widely deployed mechanism for coupling monoallelic gene activation with cell cycle exit.

## In Brief

The mechanisms controlling V_κ_ transcription and their relationships to *Ig*_*κ*_ recombination are obscure. Karki et al. demonstrate that, upon translocation to transcription factories, V_κ_-gene-containing chromatin loops are transcribed over long distances, which opens large, monoallelic, and diverse V_κ_ repertoires for subsequent V_κ_-J_κ_ recombination.

## Graphical Abstract

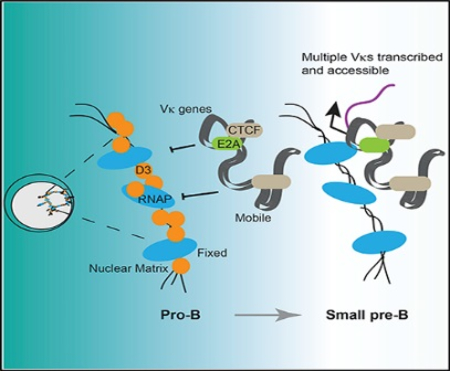

## INTRODUCTION

*Ig*_*κ*_ is composed of variable (V) and joining (J) gene clusters that undergo monoallelic recombination following stochastic choice of single V_κ_ and J_κ_ genes. Recombination is spatiotemporally regulated by stage-specific accessibility of V_κ_ and J_κ_ gene clusters and expression of recombination-activating genes (RAGs) (Clark et al., 2014; Schatz and Ji, 2011). Both the V_κ_ and J_κ_ gene clusters are repressed in pro-B cells. The J_κ_ cluster is repressed by interleukin-7 (IL-7)-receptor-activated STAT5, which both drives proliferation and directly binds the J_κ_ cluster proximate enhancer, E_κ_i, and recruits the polycomb repressive complex (PRC2) that decorates the J_κ_-C_κ_ region with H3K27me3 (Mandal et al., 2011). The choice of one *Ig*_*κ*_ allele for recombination has been correlated with monoallelic accumulation of activating histone marks in the J_κ_ cluster (Farago et al., 2012). However, these studies did not discriminate between deposition of histone marks prior to and after allelic choice and recombination. Furthermore, J_κ_ germline transcription (GLT) prior to recombination is biallelic (Amin et al., 2009), suggesting that J_κ_ accessibility does not determine allelic choice.

Whereas the J_κ_ cluster is less than 1 kb in length, the V_κ_ gene cluster stretches over approximately 3 mb and contains at least 93 (Martinez-Jean et al., 2001) functional and about 162 total V_κ_ genes organized into distal, intermediate, and proximal groups. Each group is defined by one or more topologically associating domains (TADs) formed by CCCTC-binding factor (CTCF)/ cohesion complexes (Aoki-Ota et al., 2012; Lin et al., 2012; Ribeiro de Almeida et al., 2011). The V_κ_-containing TADs contract onto the RAG-bound J_κ_ cluster, leading to V_κ_-J_κ_ recombination (Schatz and Ji, 2011).

In contrast to the J_κ_ cluster, evidence that the V_κ_ genes are epigenetically repressed in early B cell progenitors is conflicting. In pro-B and large pre-B cells, qualitative chromatin immunoprecipitation followed by deep sequencing (ChIP-seq) indicates that the V_κ_ region is not substantially marked with H3K27me3 (Mandal et al., 2011; Xu and Feeney, 2009), while in cell lines, H3K27me3 has been implicated in V_κ_ gene repression (LevinKlein et al., 2017). We have previously demonstrated that the V_κ_, but not J_κ_, cluster genes are repressed in pro-B cells by cyclin D3 bound to the nuclear matrix (NM) (Powers et al., 2012). Repression is independent of CDK4/6-mediated proliferation and cannot be complemented by cyclin D2, which does not bind the nuclear matrix. However, how cyclin D3 mediates V_κ_ repression is not known.

Herein, we demonstrate that, in pro-B cells, the V_κ_ alleles are not repressed by H3K27me3. Rather, they are repressed by cyclin D3, which prevents productive association of V_κ_ gene TADs with serine 2 phosphorylated elongating RNA polymerase II (RNAP) on NM strands (transcription factories; Iborra et al., 1996; Osborne et al., 2004) surrounding the V_κ_ genes. Cell cycle exit then opens monoallelic repertoire of V_κ_ genes that are available for recombination. These and other findings reveal a mechanism by which large and stochastic monoallelic repertories of V_κ_ genes are opened prior to recombination to J_κ_.

## RESULTS

### Monoallelic V_κ_ Transcription by Single-Cell RNA-Seq

To examine whether V_κ_ transcription prior to *Ig*_*κ*_ recombination was biallelic or monoallelic, we isolated B220^+^CD19^+^ CD43^low^IgM^-^ bone marrow (BM) small pre-B cells from a divergent F1 cross (C57BL/6 3 CAST/EiJ) and subjected them to single-cell RNA sequencing. Preliminary bulk RNA-seq on this cell population suggested that it expressed V_κ_ GLT but had not undergone extensive *Ig*_*κ*_ rearrangement (data not shown). We then used CAST/EiJ- or C57BL/6-specific SNPs to assign expressed V_κ_ genes to the CAST or B6 genome, respectively.

**Figure 1.**
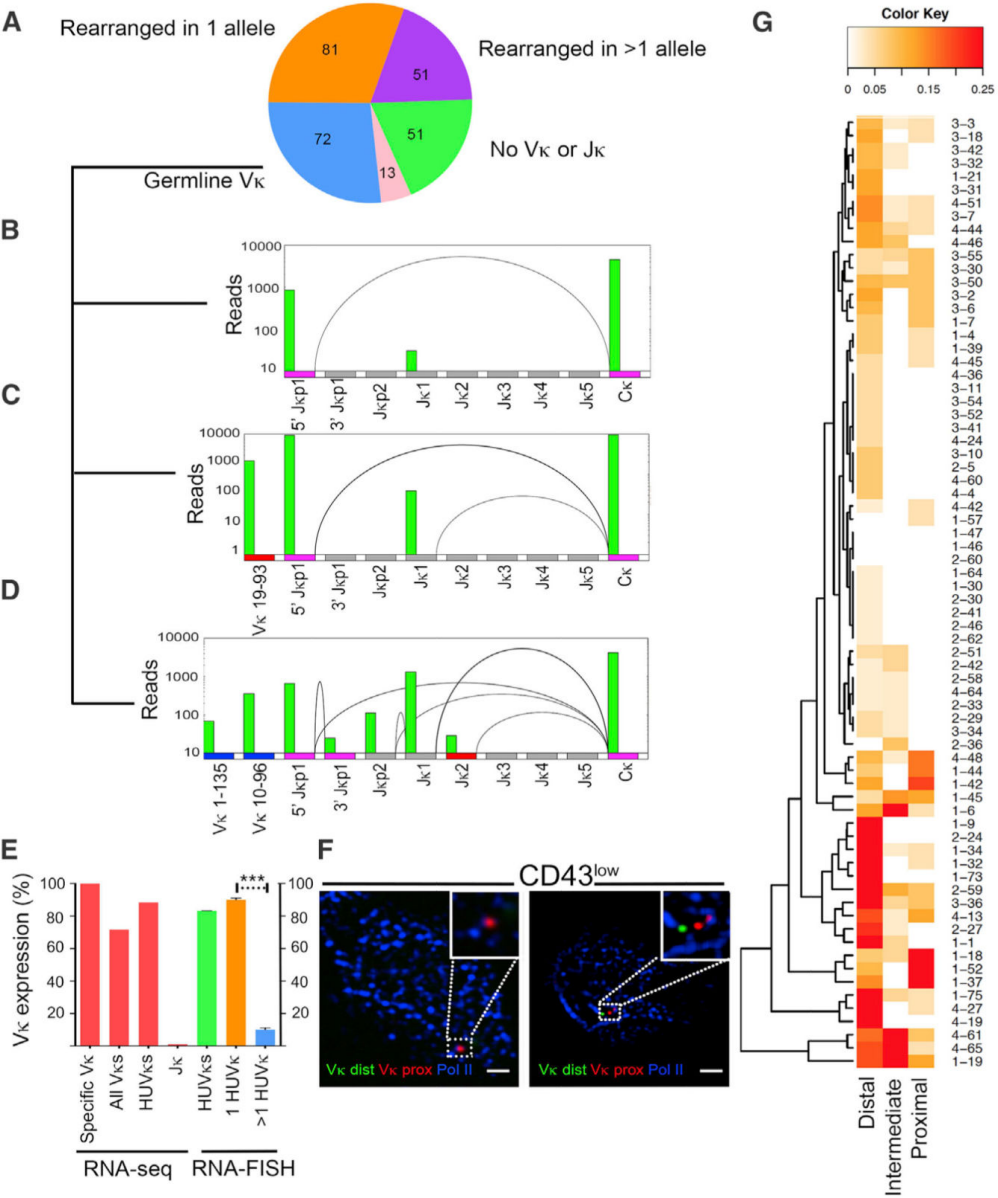
Monoallelic Activation of V_κ_ (A) Single-cell RNA-seq *Ig*_*κ*_ expression profile of CD19^+^B220^+^CD43^lo^IgM^-^ small pre-B cells. Of 268 cells captured, number of cells expressing germline V_κ_J_κ_ (blue), rearrangement in one *Ig*_*κ*_ allele (orange), rearrangement in more than one allele (purple), no V_κ_ or J_κ_ expression (green), or uncategorized (pink) are shown. (B) Representative example of biallelic germline transcription of 5′J_κ_1 promoter (magenta) without V_κ_ expression. mRNA splice products here and below are shown as arcs. (C) Representative example of biallelic germline transcription of 5′J_κ_1 promoter (magenta) with single V_κ_ expression from B6 allele (red). (D) Representative example of biallelic germline transcription of 5′J_κ_1 promoter (magenta) with multiple V_κ_ expression from CAST allele (blue). (E) Percentage of cells with monoallelic expression as determined by either RNA-seq (left bars) or RNA-FISH (right bars). From left to right, percentages of cells expressing specific V_κ_ genes monoallelically (specific V_κ_), cells expressing all V_κ_ genes from one allele (all V_κ_s), cells expressing highly used V_κ_s monoallelically (HUV_κ_s), and monoallelic J_κ_ expression (J_κ_) are shown. Based on RNA-FISH, percentage of cells monoallelically expressing one or more assayed HUV_κ_s (green bar) is shown. Percentage of cells monoallelically expressing only one (1 HUV_κ_, orange) or >1 HUV_κ_ (blue) is shown. Statistical significance was calculated on 100 cells per condition combined from two independent experiments by unpaired Student’s t test (***p < 0.001). (F) Representative images of RNA-FISH for nascent transcripts from all nine highly used V_κ_ genes combined with staining for e-Pol II (blue). Distal V_κ_s are in green and proximal in red. The scale bars represent 1 μm. (G) Heatmap of V_κ_s expressed from distal (V_κ_ 2–137 to 13–84), intermediate (V_κ_ 4–81 to 6–32), and proximal (V_κ_ 8–30 to 3–1) loops in each single cell (cell number given on right). Color reflects fraction of V_κ_ genes in indicated domains expressed in individual cells (scale 0%–25%).

From two experiments, we obtained 268 single-cell libraries (Figure 1A), with an average of 5.2 × 10^6^ 75-bp paired-end reads/cell and 83% concordant alignment rate. Of these, 51 cells did not express V_κ_ or J_κ_ genes, 81 cells had undergone recombination at single *Ig*_*κ*_ allele, and 51 had undergone recombination at both *Ig*_*κ*_ alleles and/or *Igλ*. There were 13 cells that could not be categorized. The remaining 72 cells expressed V_κ_ and/or J_κ_ GLT (Figures 1A–1D). These latter 72 cells were poised for *Ig*_*κ*_ recombination, as evident by biallelic germline J_κ_ expression originating from the distal (J_κ_p1) and proximal promoters (J_κ_p2) and absence of recombination products (Figures 1B–1D). In this cell population, there also was no evidence of V_κ_-V_κ_ recombination (data not shown).

Allelic expression was examined by assigning expressed V_κ_ genes in the 72 poised cells to either the B6 or CAST genome based on SNPs present in individual V_κ_ genes. There was good representation of V_κ_ genes expressed from both B6 and CAST alleles (Figure S1). Analysis of the 93 functional V_κ_ genes in each individual cell revealed that, for each V_κ_ gene, transcription only occurred at one allele (100% monoallelic expression; Figure 1E). Analyzing V_κ_ expression locus-wide revealed that 72% of cells expressed V_κ_s from only one allele (Figure 1E). This was in contrast to the 99% cells (71 of 72) that expressed J_κ_ biallelically (Amin et al., 2009). When we focused only on those cells expressing the nine most highly used V_κ_s (V_κ_1–135, V_κ_ 17–127, V_κ_ 9–120, V_κ_ 1–117, V_κ_10–96, V_κ_10–95, V_κ_ 6–23, V_κ_ 6–17, and V_κ_ 6–15; Aoki-Ota et al., 2012), eighty-eight percent of cells had monoallelic expression for these nine V_κ_ genes.

To confirm that highly used V_κ_ genes were primarily monoallelically expressed, we performed RNA-fluorescence *in situ* hybridization (FISH) on CD43^low^ small pre-B cells probing for all nine highly used V_κ_ GLT transcripts in the same cell, using a RNA probe cocktail (Table S1) specific for distal highly used V_κ_ (Alexa-488-labeled) and proximal highly used V_κ_ genes (AlexaFluor-647-labeled). In this assay, 83% of cells expressed highly used V_κ_ genes monoallelically (Figure 1F). Of those cells with monoallelic highly used V_κ_ expression, 90% expressed only one V_κ_ gene (Figures 1E and 1F). These data suggest that monoallelic V_κ_ transcription is a locus-wide phenomenon that provides a molecular basis for monoallelic *Ig*_*κ*_ recombination.

### Long-Range Transcription over Multiple V_κ_ Genes

In the 72 cells poised for *Ig*_*κ*_ recombination, we found that multiple V_κ_ genes were expressed in 92% of cells with up to 16 per cell. Distal V_κ_ genes were expressed in almost all cells (Figure 1G), with 90% of cells expressing more than one. Forty-six percent of cells expressed both distal and proximal V_κ_ genes (Figure 1G). Forty-five of 72 poised cells (60%) expressed highly used V_κ_s (Aoki-Ota et al., 2012), suggesting that these gene segments are commonly accessible (Figure S1).

Interestingly, in those cells expressing intermediate and/or proximal V_κ_ genes, transcription of the *Igλ* locus was common (Figure S1). These cells had not undergone *Ig*_*κ*_ recombination, suggesting that the opening of *Igλ* is not a consequence of *Ig*_*κ*_ recombination. Rather, our data suggest overlap in the mechanisms regulating accessibility at both loci.

**Figure 2.**
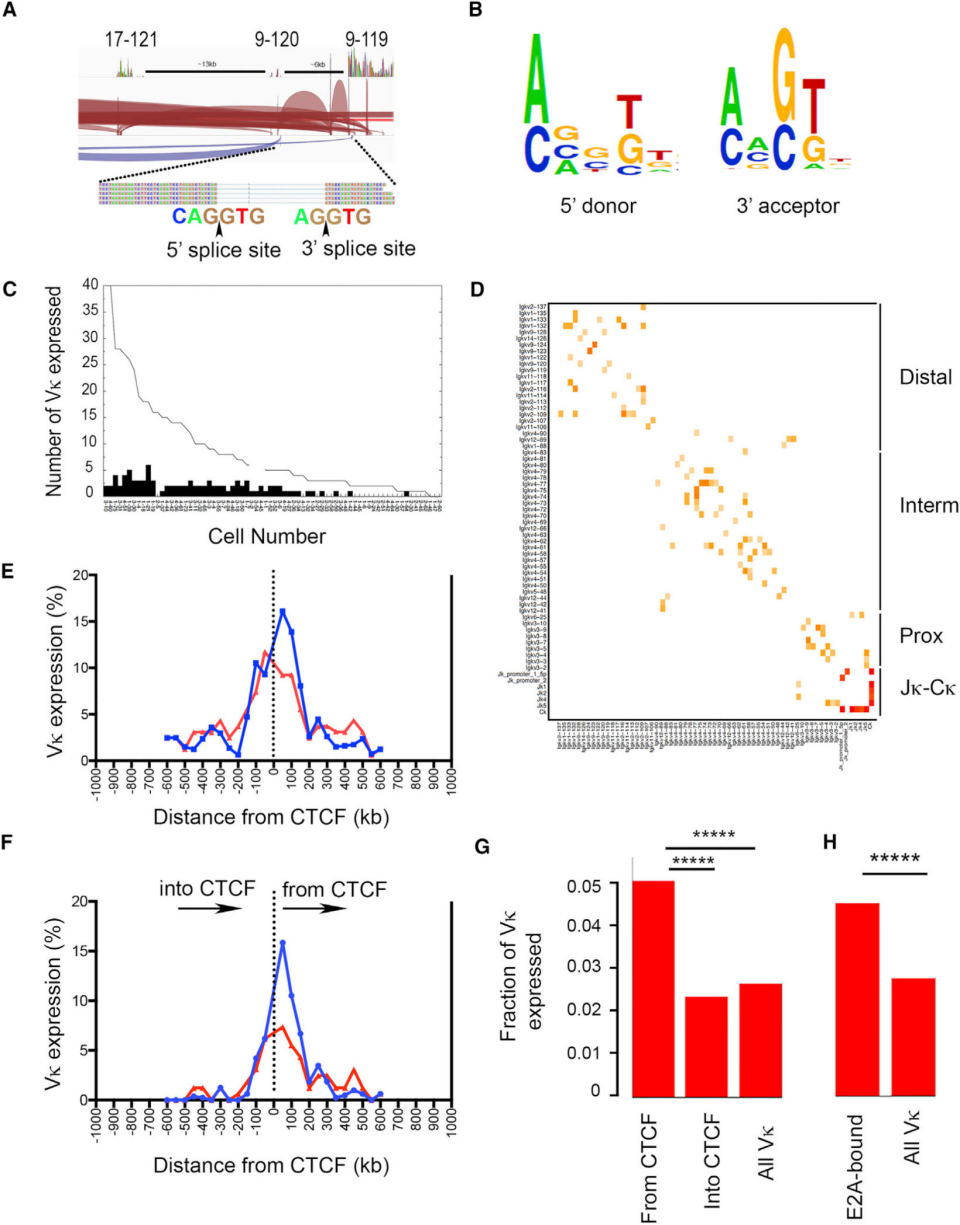
Monoallelic Expression of V_κ_ Chromatin Loops (A) Representative example of V_κ_-V_κ_ splicing from cell no. 1–75 (see Figure S2). Individual sense transcripts are shown in red and antisense transcripts shown in purple. Arcs represent indicated spliced transcripts, including splicing between V_κ_ 17–121, 9–120, and 9–119. 5′ and 3′ splice junctions for V_κ_ 9–120 and V_κ_ 9–119 are shown. Black arrow represents site where transcript is spliced. Genomic distances between V_κ_ genes are shown. (B) Consensus splice junction motifs obtained from 5′ donor and 3′ acceptor sites when all splice junctions were analyzed. (C) Total number of expressed V_κ_s interpolated from splicing from 72 cells poised for recombination. Black bars represent number of highly used V_κ_s per cell. (D) Heatmap of spliced V_κ_ and J_κ_s across *Ig*_*κ*_ locus for all 72 poised single cells. Color reflects fraction of genes that underwent splicing (key in Figure 1G). Distal, intermediate, and proximal topologically associated domains (TADs) of *Ig*_*κ*_ are shown on the right. (E and F) Frequency of V_κ_ expression as a function of distance from CTCF sites. In (E), V_κ_ expression relative to CTCF sites with V_κ_s 5′ to CTCF on left and those 3′ plotted on right are shown. Plot regardless of V_κ_ gene orientation is shown. In (F), plot of expression of V_κ_ genes either oriented toward or away from CTCF sites as a function of distance is shown. For both plots, observed expression frequencies are shown in blue and expected frequencies if expression was randomly distributed shown in red. (G) The number of V_κ_s expressed that are oriented away from or into CTCF sites over total V_κ_s oriented away from or into CTCF sites is shown as “from CTCF” and “into CTCF” fraction (Figure 1F), respectively. All expressed V_κ_s over total V_κ_s are shown as “all V_κ_” fraction. Fisher’s exact test was performed between group pairs over all single cells. (From CTCF to into CTCF: *****p = 4.37 × 10^−13^; from CTCF to all V_κ_: *****p = 7.23 × 10^−9^). (H) The number of V_κ_s expressed that have E2A at promoters in pro-B cells over total E2A-promoter loaded V_κ_s is shown as “E2A-associated” fraction, and all expressed V_κ_s over total V_κ_s are shown as all V_κ_ fraction. Fisher’s exact test was performed between the two groups over all single cells (*****p = 4.9 × 10^−39^).

Remarkably, V_κ_ to V_κ_ mRNA splicing was common. Shown in Figure 2A is an example demonstrating splicing between three V_κ_ genes (17–121, 9–120, and 9–119) in a single cell. In this example, splicing occurred between adjacent and non-adjacent V_κ_ genes. V_κ_-V_κ_ splicing also occurred in the antisense direction. A similar pattern was observed in all cells, with splicing occurring both between adjacent V_κ_ genes and those separated by up to 32 intervening V_κ_ genes (data not shown). De novo analysis of all splice junctions revealed canonical 5′ donor and 3′ acceptor consensus sequences consistent with conventional mRNA splicing (Figure 2B).

Interpolation of intervening V_κ_s between spliced V_κ_s indicated that up to 40 V_κ_ genes were transcribed in a single cell and that 70% of cells transcribed at least one highly used V_κ_ gene (Figure 2C). Interpolation also indicated that transcription occurred over very long distances. The average inferred transcript length was 425 kb, with a maximum length of 1.1 mb. However, there were clear transcription boundaries. Examination of the distribution of V_κ_-V_κ_ splicing revealed that it was strictly limited by boundaries predicted by CTCF sites to approximate the distal, intermediate, and proximal TADs (Lin et al., 2012; Figures 2D and S2). Germline J_κ_-C_κ_ splicing was common (Figures 2D and S2). These data suggest that expression of multiple V_κ_ genes occurs by transcriptional readthrough and is common within TADs.

### V_κ_ Transcription Initiated at CTCF Sites and E2A-Bound Promoters

Transcription has been shown to occur at or near CTCF sites, whereby CTCF can directly associate with RNAP and anchor transcription (Chernukhin et al., 2007). We therefore examined whether V_κ_ transcription occurred close to CTCF-bound sites in single cells. Comparing published pro-B Hi-C data and CTCF ChIP-seq data, there are eight CTCF-bound sites (S1–S8) within the *Ig*_*κ*_ locus with high likelihood for loop formation (Figures S1 and S2; Choi and Feeney, 2014; Lin et al., 2012). The distal V_κ_ TAD is bound by S1 and S5, the intermediate TAD by S5 and S6, and the proximal TAD by S6 and S8 (Figures S1 and S2). We first plotted V_κ_ frequencies as a function of genomic distance from individual CTCF sites and compared this to predicted frequencies considering random distribution of V_κ_ expression (Figure 2E). For this analysis, V_κ_ genes 5′ to a CTCF-bound site were plotted as negative distances and those 3′ as positive distances. As can be seen, there was an overall bias to transcribe V_κ_ genes near CTCF-bound sites (Figure 2E).

Throughout the *Ig*_*κ*_ locus, V_κ_ genes occur in both orientations (Figure S1). We next examined V_κ_ gene transcription bias due to their orientation from CTCF sites. We plotted expressed V_κ_ genes as a function of orientation and distance from CTCF sites. As seen in Figure 2F, there was a strong bias to transcribe V_κ_ genes oriented away from CTCF-bound sites compared to a random distribution and this bias persisted out to 150 kb.

Not all V_κ_ genes are functional (Martinez-Jean et al., 2001; Figure S1). We next assessed functional V_κ_ gene transcription bias. We plotted expressed V_κ_ genes as a fraction of total known functional V_κ_ genes in each indicated orientation relative to CTCF-bound sites. As can be seen, there was a significant bias for transcribing functional V_κ_ genes oriented away from CTCF sites compared to transcribing those oriented toward CTCF sites or when no orientation bias was considered (Figure 2G).

The transcription factor E2A is important for *Ig* gene transcription (Romanow et al., 2000). We therefore examined the relative frequency of V_κ_ gene expression for those with promoters pre-bound by E2A in pro-B cells (E2A ChIP-seq; Lin et al., 2010) compared to the frequency of expression for all V_κ_ genes. As demonstrated in Figure 2H, E2A-associated V_κ_s were expressed at a significantly higher frequency compared to overall V_κ_s. These data suggest V_κ_ transcription can be initiated at or near CTCF-bound sites and at the promoters of V_κ_ genes preloaded with transcription factors.

### V_κ_ Genes Are Not Repressed by H3K27me3 in Pro-B Cells

We next asked how V_κ_ transcription was regulated. Transcription is often repressed epigenetically, with histone methylation (H3K27me3) being a common mechanism. However, the data regarding the role of H3K27me3 in V_κ_ gene repression have been conflicting (Levin-Klein et al., 2017; Mandal et al., 2011; Xu and Feeney, 2009). In part, this confusion reflects the limitation of conventional ChIP-seq, which lacks internal standards and is therefore qualitative. Therefore, to determine whether the V_κ_ genes are, or are not, marked with H3K27me3, we performed an internally calibrated ChIP (ICeChIP) (Grzybowski et al., 2015). The ICeChIP both performs an *in situ* antibody specificity test and displays data on a biologically meaningful scale, the histone modification density (HMD), representing the fraction of all nucleosomes bearing a specific histone mark at a given genomic locus. These attributes allowed us to quantify the amount of H3K27me3 at the V_κ_ genes on an absolute scale.

**Figure 3.**
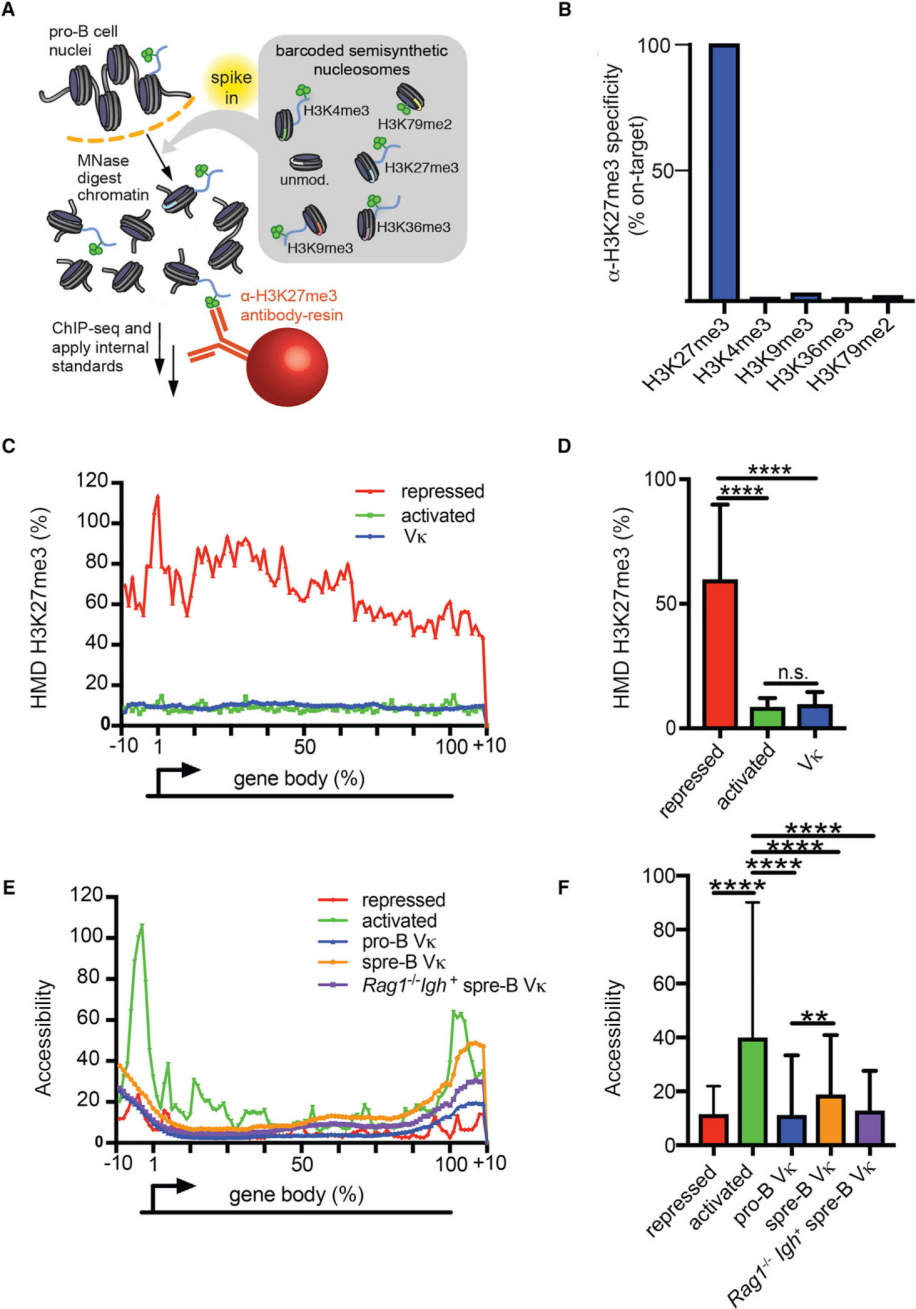
In Pro-B Cells, the V_κ_ Genes Lack Appreciable H3K27me3 (A) Schematic representation of ICeChIP-seq. (B) ICeChIP-seq-based specificity measurement of αH3K27me3 (CST C36B11) antibody. Specificity is expressed as a fraction of normalized H3K27me3 nucleosome capture. (C) Meta-analysis of H3K27me3 ICeChIP displaying the average HMD over the length of each gene body for the following gene sets: V_κ_ regions; activated genes; and repressed genes in pro-B cells (Table S2). (D) H3K27me3 HMD in pro-B cells comparing V_κ_ gene segments to activated genes and repressed genes as in (C). (E) Meta-analysis of accessibility (ATAC-seq) displaying the average HMD over the length of each gene body for the following gene sets: V_κ_ regions in pro-B cells (pro-B); V_κ_ regions in small pre-B cells (spre-B); V_κ_ regions in *Rag1*^*−/−*^*Igh*^+^ small pre-B cells; activated genes in pro-B cells; and repressed genes in pro-B cells. (F) Accessibility calculated from – 10% to +10% relative to the transcription start site (TSS) for each gene set displayed. (D–F) Statistical significance was determined by ANOVA (p < 0.0001 and p < 0.0001, respectively) in combination with Tukey’s multiple comparison test. Error bars represent the average ± SD. **p ≤ 0.01; ****p ≤ 0.0001.

Therefore, chromatin was isolated from *Rag2*^*−/−*^ pro-B cell nuclei and then “spiked” with semisynthetic nucleosome standards, each containing a single histone post-translational modification (H3K27me3, H3K4me3, H3K9me3, H3K36me3, or H3K79me2) and a unique bar-coded DNA strand (Figure 3A). Samples were then immunoprecipitated with antibodies specific for H3K27me3 and subjected to next generation sequencing.

Analysis of the ICeChIP data revealed that the immunoprecipitating antibody we used specifically bound H3K27me3 and did not appreciably react with other histones methyl modifications (Figure 3B). To establish a positive control, we averaged the H3K27me3 HMD at representative genes, which were marked with H3K27me3 and not expressed in pro-B cells (Mandal et al., 2011; Table S2). As a negative control, we averaged the H3K27me3 HMD at representative genes actively transcribed in pro-B cells. Interestingly, H3K27me3 HMD at inactive V_κ_ genes was much lower than that observed in other repressed genes and was comparable to levels observed in activated genes (3C and 3D). These data suggest that, prior to recombination, the V_κ_ genes are not repressed by a H3K27me3-dependent mechanism.

We then examined V_κ_ accessibility in pro-B and small pre-B wild-type (WT) cells using the assay for transposase-accessible chromatin (ATAC)-seq (Figures 3E and 3F). In the activated and repressed control genes used above, there were corresponding substantial differences in accessibility density, especially immediately upstream of the gene body. This likely reflects differences in promoter accessibility. In contrast, there were only small differences in V_κ_ gene accessibility between pro-B cells, where the V_κ_ genes are repressed, and small pre-B cells, where V_κ_ genes are transcribed and undergo *Ig*_*κ*_ recombination. Accessibility of the V_κ_ genes was similar in *Rag1*^*−/−*^*Igh*^+^ small pre-B cells, which are primed for *Ig*_*κ*_ recombination. Therefore, V_κ_ gene transcription and susceptibility to recombination are not associated with intrinsic changes in V_κ_ gene accessibility. These findings, in conjunction with our observation that the V_κ_ genes are not repressed by H3K27me3, suggest that nonconventional mechanism(s) regulate V_κ_ gene transcription.

### The V_κ_ Repressor, Cyclin D3, Associates with NM-RNAP

**Figure 4.**
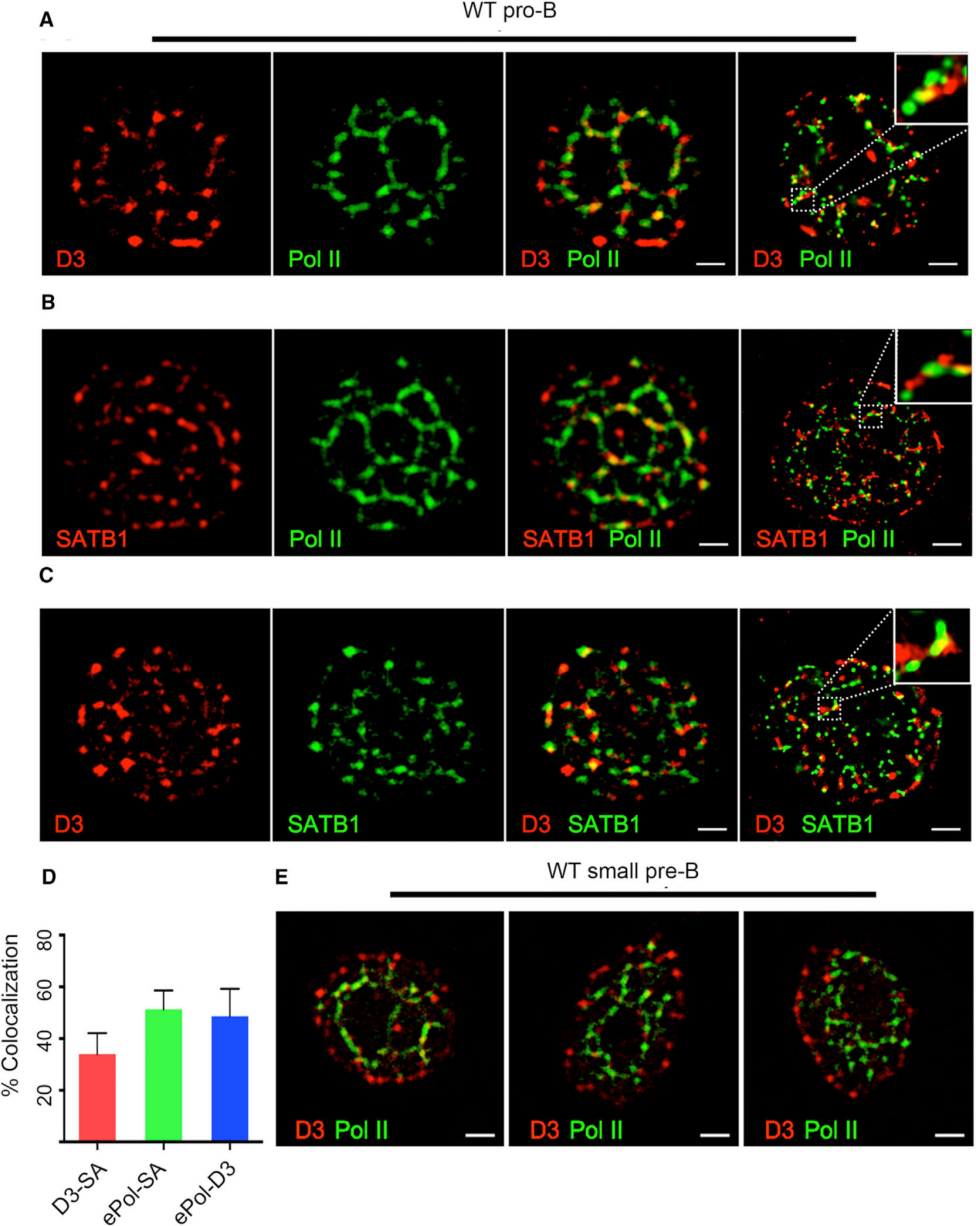
Cyclin D3 Is Assembled with RNAP on the Nuclear Matrix (A) Representative confocal images (from 40 cells; n = 2 experiments) of WT pro-B cells washed 10× (CSK+0.5%Triton) to remove soluble nuclear proteins and then fixed and stained with antibodies specific for cyclin D3 and e-Pol II (RNAP). Super-resolution image of similarly stained WT pro-B cells (right panel) is shown. The scale bars represent 1 μm. (B) Representative confocal images (40 cells; n = 2 experiments) of WT pro-B cells washed and fixed as above and then stained with antibodies specific for SATB1 and e-Pol II. Super-resolution image of similarly stained WT pro-B cells (right panel) is shown. The scale bars represent 1 μm. (C) Representative confocal images (40 cells; n = 2 experiments) of WT pro-B cells washed and fixed as above and then stained with antibodies specific for SATB1 and cyclin D3. Super-resolution image of similarly stained WT pro-B cells (right panel) is shown. The scale bars represent 1 μm. (D) Percent co-localization of elongating RNAP-D3 (ePol-D3), D3-SATB1 (D3-SA), and RNAP-SATB1 (ePol-SA) stains calculated by using Manders on 30 2Dconfocal images per samples (n = 2 experiments). (E) Representative confocal images (40 cells; n = 2 experiments) of WT small pre-B cells washed as above and stained with antibodies specific for cyclin D3 and RNAP. The scale bars represent 1 μm.

Our earlier work has revealed that V_κ_ transcription in pro-B cells is repressed by cyclin D3 bound to the NM (Powers et al., 2012). RNAP is also assembled on the NM in supra-molecular complexes termed “transcription factories.” Translocation of genes to such fixed sites has been suggested as a mechanism for transcriptional activation (Iborra et al., 1996; Osborne et al., 2004). However, how this mechanism of transcription is regulated is unknown. To examine whether there might be a functional relationship between NM-bound cyclin D3 and RNAP, we used confocal microscopy to visualize their spatial relationships on the NM. WT pro-B cells were permeabilized and then washed extensively under mild conditions (cytoskeletal stabilizing buffer [CSK]+0.5% Triton) to remove soluble nuclear proteins (Figure S3A; Sawasdichai et al., 2010). Cells were then fixed, stained with antibodies specific for cyclin D3 or RNAP, and visualized by confocal microscopy. These studies revealed that, in pro-B cells, NM-bound cyclin D3 either co-localized (55% Mander’s coefficient) or was closely apposed with RNAP on apparent strands that formed a “cage-like” pattern (Figures 4A and 4D). Co-staining with antibodies specific for the NM-bound molecule, special AT-rich sequence-binding protein 1 (SATB1), revealed its colocalization with both cyclin D3 and RNAP (35% and 50%, respectively; Figures 4B-4D and S3B) in a similar cage-like pattern. To better visualize these RNAP/cyclin D3 complexes, we used super-resolution microscopy (Leica Ground State Depletion), which provides 20-nm resolution in the X-Y plane. This higher resolution revealed that cyclin D3, RNAP, and SATB1 were intertwined, forming nanometer-scale fibrils throughout the nucleus (Figures 4A-4C and S3B, far right panels).

At the small pre-B cell stage, cyclin D3 transcription is repressed, enabling cells to exit cell cycle and initiate *Ig*_*κ*_ recombination (Cooper et al., 2006). Interestingly, in WT small pre-B cells, cyclin D3 had largely translocated to the periphery of the nucleus away from RNAP (Figure 4E). These data reveal that the spatial relationship between cyclin D3 and RNAP is developmentally regulated.

### Cyclin D3 Regulates Monoallelic V_κ_ Association with NM-RNAP

**Figure 5.**
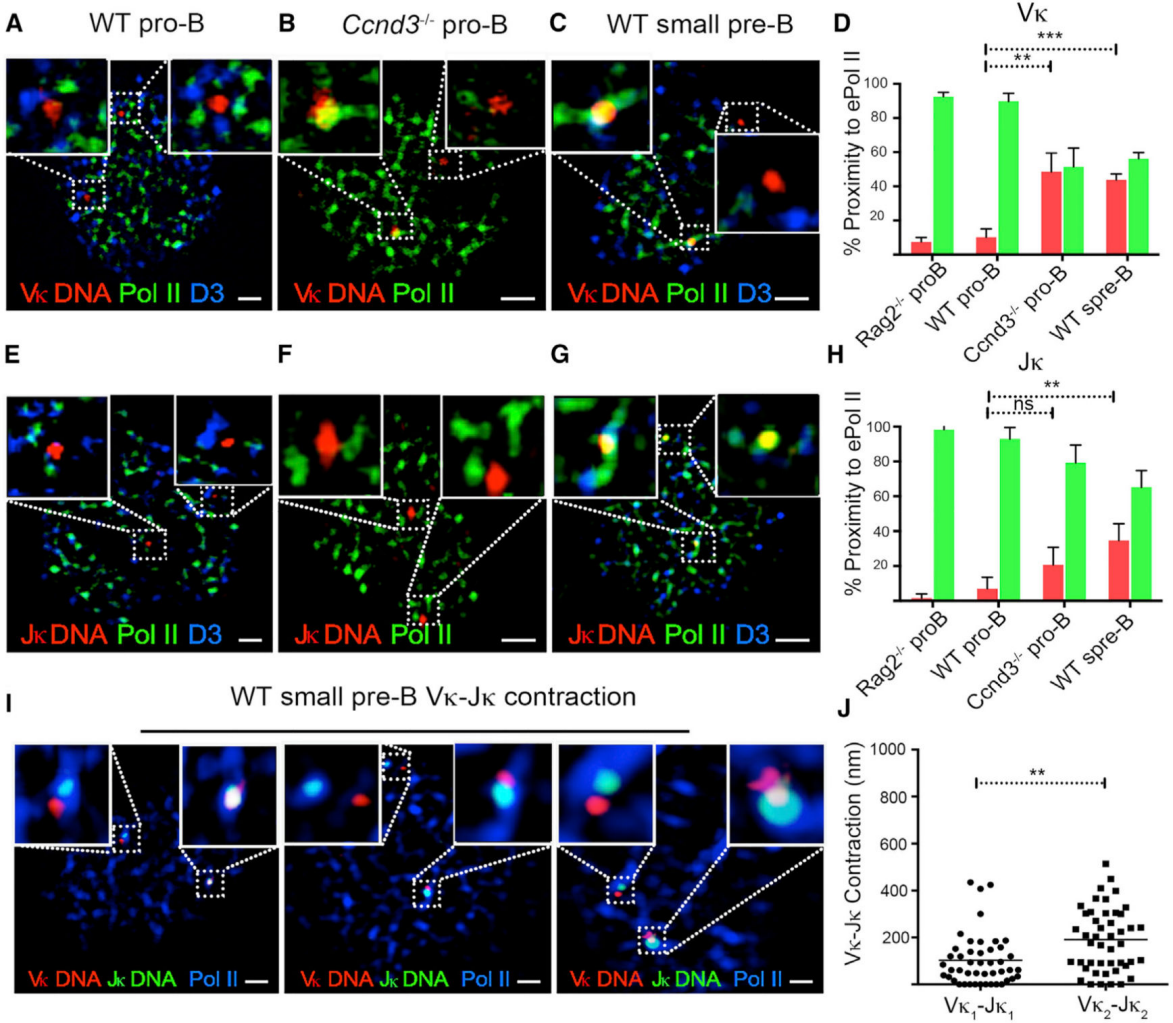
Cyclin D3 Regulates V_κ_, but Not J_κ_, Association with RNAP (A) Representative confocal image (50 cells; n = 3 experiments) of WT pro-B cells washed extensively to remove soluble nuclear proteins and then hybridized with V_κ_ DNA probe (RP23–182E6) spanning 10 distal V_κ_ genes (V_κ_2–113 to 1–122) and stained with antibodies specific for cyclin D3 and e-Pol II (RNAP). The scale bar represents 1 μm. (B) Representative confocal image (50 cells; n = 3 experiments) of *Ccnd3*^*−/−*^ pro-B cells washed, hybridized with V_κ_ DNA probe (RP23–182E6), and stained with antibodies specific for e-Pol II. The scale bar represents 1 μm. (C) Representative confocal image (50 cells; n = 3 experiments) of WT small pre-B cells washed, hybridized with V_κ_ DNA probe (RP23–182E6), and stained with antibodies specific for cyclin D3 and e-Pol II. The scale bar represents 1 μm. (D) Percent J_κ_ co-localized (red) and not co-localized (green) to e-Pol II scored on confocal images of 50 nuclei per sample (n = 3 experiments), and plotted for each sample. Co-localization scored when at least one V_κ_ allele engaged e-Pol II. Statistical significance was calculated by unpaired Student’s t test (**p < 0.01 and ***p < 0.001). (E) Representative confocal image (50 cells; n = 3 experiments) of WT pro-B cells washed, hybridized with J_κ_ DNA probe (RP24–387E13) spanning J_κ_- C_κ_, and stained with antibodies specific for cyclin D3 and e-Pol II. The scale bar represents 1 μm. (F) Representative confocal images (50 cells; n = 3 experiments) of *Ccnd3*^*−/−*^ pro-B cells washed, hybridized with J_κ_ DNA probe (RP24–387E13), and stained with antibodies specific for e-Pol II. The scale bar represents 1 μm. (G) Representative confocal images (50 cells; n = 3 experiments) of WT small pre-B cells washed, hybridized with J_κ_ DNA probe (RP24–387E13) spanning J_κ_C_κ_, and stained with antibodies specific for cyclin D3 and e-Pol II. The scale bar represents 1 μm. (H) Percent J_κ_ co-localized (red) and not co-localized (green) to e-Pol II scored on confocal images of 50 nuclei per sample (n = 3 experiments) and plotted for each sample. Co-localization was scored when at least one J_κ_ allele engaged e-Pol II. Statistical significance was calculated by unpaired Student’s t test (**p < 0.01). (I) Representative confocal images (40 cells; n = 2 experiments) of WT small pre-B cells, washed to remove soluble nuclear proteins, hybridized to V_κ_ DNA probe RP23–182E6 (red) and J_κ_ DNA probe RP24–382E13 (green), and stained with antibodies specific for e-Pol II (blue). The scale bars represent 1 μm. (J) Minimum distances between V_κ_ and J_κ_ in WT small pre-B cells plotted for the allele in each cell closer to RNAP (V_κ_1-J_κ_1) and the allele farther removed from RNAP (V_κ_2-J_κ_2). Distances between V_κ_ and J_κ_ were calculated using Euclidean Distance Transformation on Imaris. Analysis was performed on cells imaged as in (I). Statistical significance was calculated (n = 37) by paired Student’s t test (**p < 0.01).

We next examined the spatial relationships between cyclin D3, RNAP, and the V_κ_ gene cluster. WT or *Ccnd3*^*−/−*^ pro-B cells were washed as above, fixed, and then subjected to FISH with a 488-labeled bacterial artificial chromosome (BAC) probe, RP-23 182E6, that binds a 0.2-mb region spanning 10 distal V_κ_ gene segments (V_κ_ 2–113 to 1–122), followed by staining with antibodies specific for cyclin D3 and RNAP (immunoFISH). In WT pro-B cells, both V_κ_ alleles were surrounded by NM RNAP-cyclin D3 complexes (Figure 5A). However, V_κ_ genes and RNAP rarely co-localized (<5%; Figures 5A and 5D). In contrast, in *Ccnd3*^*−/−*^ pro-B cells and WT small pre-B cells, V_κ_ genes frequently co-localized with NM RNAP (35%–40%; Figures 5B–5D). Co-localization of V_κ_ genes to RNAP was specific to *Ig*_*κ*_, as *TCRβ* (Vβ) was rarely found to associate with RNAP in WT small pre-B cells (Figure S4A).

Almost invariably (>95%), only one V_κ_ allele co-localized with RNAP in *Ccnd3*^*−/−*^ pro-B and WT small pre-B cells (Figures 5B–5D), consistent with single-cell RNA sequencing (scRNAseq) findings. Furthermore, in both *Ccnd3*^*−/−*^ pro-B cells and WT small pre-B cells, the early replicating allele, previously shown to undergo recombination (Farago et al., 2012), associated with RNAP (V_κ_ doublet; Figures S4B and S4C). This latter observation suggests that V_κ_ transcription initiates at the *Ig*_*κ*_ allele fated to recombine first.

We next examined the association of J_κ_ cluster with RNAP and its regulation by cyclin D3 using a BAC probe spanning the entire J_κ_ cluster and Ck (RP24–387E13). In contrast to V_κ_, we found that J_κ_ did not co-localize with NM-RNAP in WT pro-B or *Ccnd3*^*−/−*^ pro-B cells (Figures 5E, 5F, and 5H). This is consistent with previous findings that J_κ_ GLT is not regulated by cyclin D3 but by the reciprocal action of STAT5 and E2A (Mandal et al., 2011; Powers et al., 2012). In WT small pre-B cells, both J_κ_ alleles co-localized (40%) with RNAP (Figures 5G and 5H; Amin et al., 2009). These results suggest that monoallelic V_κ_ transcription is achieved by monoallelic co-localization of V_κ_ to NMRNAP. Furthermore, cyclin D3 represses co-localization of the V_κ_ gene cluster, but not J_κ_ cluster, with NM-RNAP.

V_κ_-J_κ_ contraction is necessary for recombination. To examine whether the V_κ_ allele co-localizing with RNAP marks the allele that undergoes V_κ_-J_κ_ contraction, we performed 2-color V_κ_ and J_κ_ DNA-FISH in combination with RNAP immunofluorescence (IF) on WT small pre-B cells (Figures 5I, 5J, and S4F). As demonstrated, the *Ig*_*κ*_ allele in which V_κ_ genes were co-localized with RNAP (V_κ_1-J_κ_1) was more contracted (mean < 200 nm) than the allele in which V_κ_ genes were not co-localized with RNAP (mean > 200 nm). In contrast, in WT pro-B, there was no V_κ_ gene translocation to RNAP and both alleles were not contracted (Figures S4D and S4G). Monoallelic V_κ_ gene translocation to RNAP and contraction was also observed in *Ccnd3*^*−/−*^ pro-B cells (Figures S4E and S4H), where J_κ_ is still repressed (Figure S4I; Powers et al., 2012). These findings suggest that the V_κ_ gene cluster allele, which translocates to RNAP, marks the contracting and therefore recombining allele. Furthermore, contraction can happen independently of both J_κ_ GLT and V_κ_-J_κ_ recombination.

**Figure 6.**
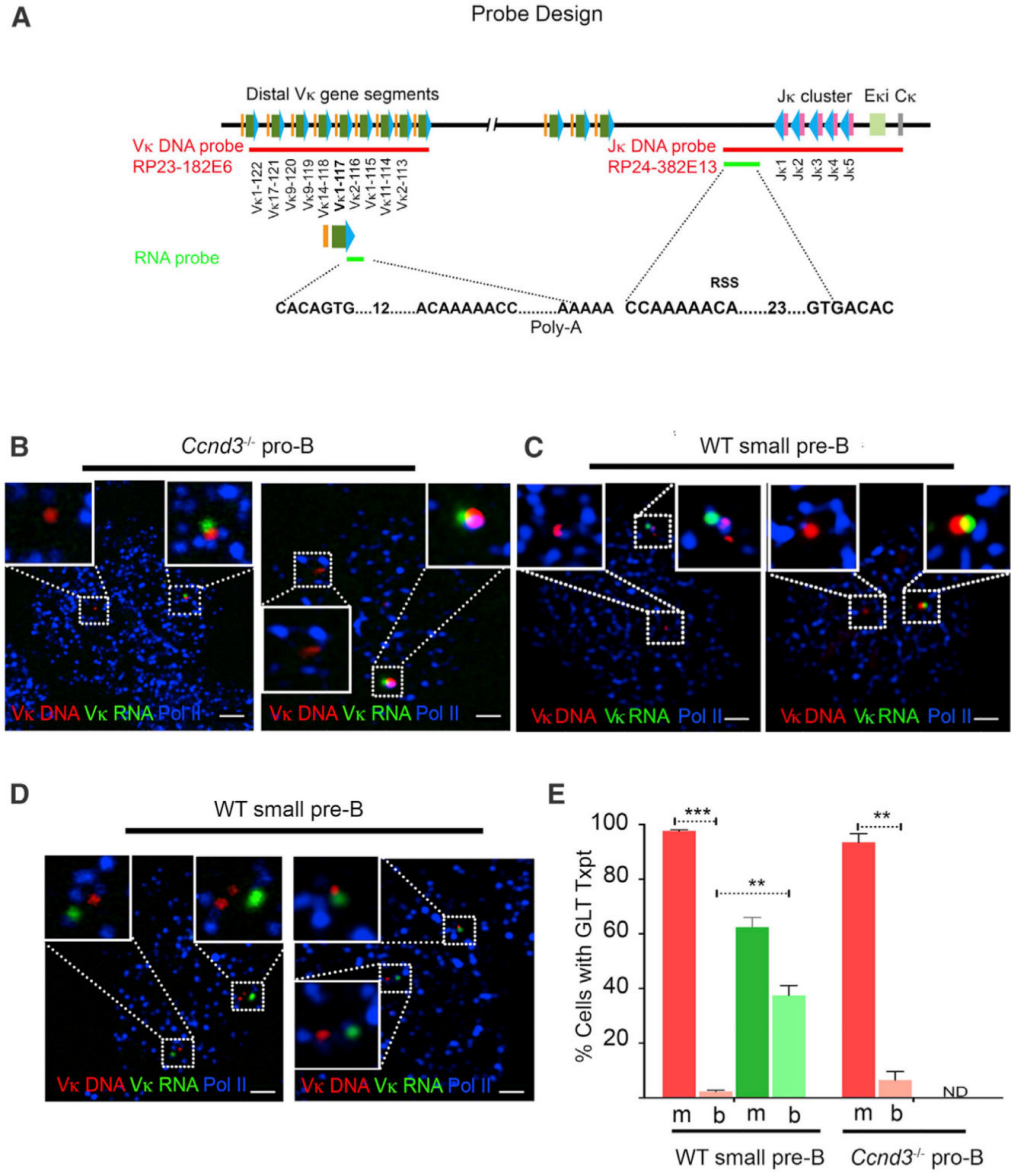
Monoallelic V_κ_ Transcription Repressed by Cyclin D3 (A) Schematic of DNA and RNA probes used in the study. V_κ_ DNA probe targets distal V_κ_ gene segments (V_κ_ 113–122), whereas RNA probe only targets a single V_κ_ gene (1–117). The RNA probe binds to region 3′ of the RSS site and therefore targets unrearranged, germline transcripts. J_κ_ DNA probe targets J_κ_-C_κ_ region, whereas the J_κ_ RNA probe targets germline sequences downstream of the distal promoter (5′ of J_κ_1) and 3.5 kb upstream of Jk1 (Table S3). (B) Representative confocal images (40 cells; n = 2 experiments) of *Ccnd3*^*−/−*^ pro-B cells, washed extensively to remove soluble nuclear proteins and then hybridized to V_κ_ DNA probe RP23–182E6, and stained with antibodies specific for e-Pol II followed by hybridization with RNA probe targeting 3′ of V_κ_ 1–117. Shown are two representative examples, arranged horizontally. The scale bars represent 1 μm. (C) Representative confocal images (40 cells; n = 2 experiments) of WT small pre-B cells, washed, hybridized, and stained as in (B). Shown are two representative examples, arranged horizontally. The scale bars represent 1 μm. (D) Representative confocal images (40 cells; n = 2 experiments) of WT small pre-B cells, washed, hybridized with J_κ_ DNA probe RP24–387E13, and stained with antibodies specific for e-Pol II followed by hybridization with RNA probe targeting 5′ to J_κ_. Shown are two representative examples, arranged horizontally. The scale bars represent 1 μm. (E) Percent WT small pre-B cells and *Ccnd3*^*−/−*^ pro-B cells with V_κ_ (red) or J_κ_ (green) germline transcripts scored on 50–60 nuclei per sample (n = 2 experiments) and further scored for monoallelic (m) versus biallelic (b) transcription. Statistical significance was calculated by unpaired Student’s t test (**p < 0.01 and ***p < 0.001).

To equate V_κ_ and RNAP co-localization with transcription, we combined DNA-FISH with RNA-FISH using a RNA probe for a single germline V_κ_ (highly used V_κ_ 1–117) transcript encompassed within the RP-23 182E6 BAC probe used for DNA FISH (Figure 6A; Table S3). In both *Ccnd3*^*−/−*^ pro-B and WT small pre-B cells, we detected a V_κ_ 1–117 GLT either co-localized or in close proximity to a single V_κ_ gene/RNAP complex (Figures 6B, 6C, S5A, and S5B). Consistent with scRNA-seq data, in those cells expressing V_κ_ 1–117 germline transcripts, transcription was monoallelic in 95%–98% of WT small pre-B cells and *Ccnd3*^*−/−*^ pro-B cells (Figure 6E).

We then examined J_κ_ GLT in WT small pre-B cells using a RNA probe complementary to a region 5′ of Jk1 and 3′ of the distal promoter (Figure 6A; Table S3). Biallelic GLT J_κ_ was noted in nearly 40% of cells that had transcripts (Figures 6D, 6E, and S5C). These data confirm that monoallelic V_κ_ translocation to RNAP leads to monoallelic transcription, whereas biallelic J_κ_ translocation to RNAP leads to biallelic transcription.

### Cyclin D3 Is a General Repressor of Monoallelic Genes

**Figure 7.**
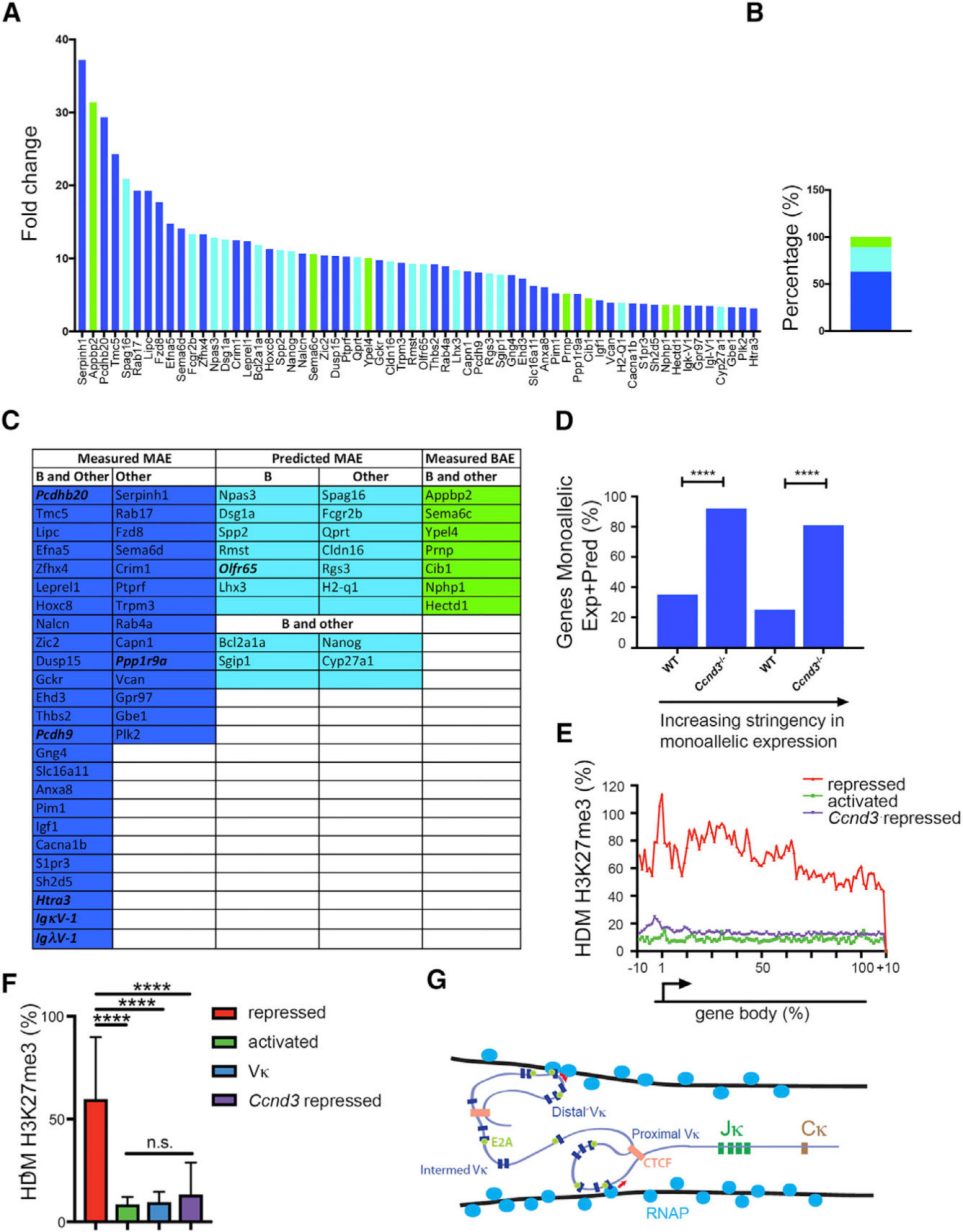
Cyclin D3 Represses Other Mono-allelically Expressed Genes (A) Upregulated genes in *Ccnd3*^*−/−*^ pro-B cells on microarray hierarchically ranked by fold increase over WT pro-B cells ( = >3-fold) plotted. Genes experimentally measured as monoallelic in B and other tissues are shown in blue, those predicted monoallelic in B cells and other tissues are shown in light blue, and those that are measured biallelic in B cells and other tissues are shown in green. (B) Percentage of genes measured as in (A). Monoallelic (MAE) in B cells and other tissues (blue), predicted monoallelic (light blue), and biallelic (BAE) in B cells and other tissues (green) are shown. (C) List of monoallelic and biallelic genes in *Ccnd3*^*−/−*^ pro-B cells, where genes are color coded as in (A) and (B). Imprinted genes (Ppp1r9a and Htra3), olfactory receptor genes (Olfr65), Ig_κ_ variable (Ig_κ_V-1), Igλ (IgλV-1) variable, and protocadherin genes (Pcdhb20 and Pcdh9) are bold and italicized. (D) Percentage of randomly monoallelic genes expressed in WT and *Ccnd3*^*−/−*^ pro-B cells. Two criteria are used. Bars on left show fraction of expressed genes known to be monoallelically expressed in at least three of five B cell lines and in at least five other tissues. Bars on right show fraction of expressed genes known to be monoallelically expressed in at least ten other tissues. Statistical significance was measured by Fisher’s exact t test (left, ****p = 1.3 × 10–07; right, ****p = 2.4 × 10–09). (E and F) Metagene analysis of H3K27me3 ICeChIP displaying the average HMD over the length of each gene body for the following gene sets: *Ccnd3* repressed genes; activated genes; and repressed genes in pro-B cells (E). H3K27me3 HMD in pro-B cells comparing Ccnd3 repressed genes to V_κ_ gene-segments, activated genes, and repressed genes (F) is shown. Statistical significance was determined by ANOVA p < 0.0001 in combination with Tukey’s multiple comparison test. Error bars represent the average ± SD. ****p ≤ 0.0001. (G) Model of V_κ_ repertoire diversity. Our data indicate that V_κ_ genes are surrounded by transcription factories (NM-bound RNAP; cross-section shown). With loss of cyclin D3, V_κ_-gene-containing TADs can stochastically engage one or more transcription factories with transcription either being initiated at E2A-bound promoters (red arrows) or CTCF sites.

Deletion of cyclin D3 induced expression of approximately 200 genes in addition to V_κ_ genes in pro-B cells (Powers et al., 2012). These genes were hierarchically ranked by fold increase overexpression in WT pro-B cells, and those with equal or greater than a three-fold increase were plotted (Figure 7A). We next interrogated the Database of Autosomal Monoallelic Expression (dbMAE) (Savova et al., 2016; Figures 7A–7C). In this database, genes are considered to have random monoallelic expression (MAE) if they demonstrate monoallelic expression in cell clones from several different tissues. Of the 65 genes upregulated three-fold or higher in *Ccnd3*^*−/−*^ pro-B cells, 62 could be assessed for MAE either directly or indirectly by epigenetic marks. Interestingly, 63% (39 of 62) of cyclin-D3-repressed genes have been directly demonstrated to be monoallelically expressed in at least five different F1 tissues, including B cells (dark blue bars, Figure 7A). Only 11% (7) genes were biallelically expressed in multiple tissues (green, Figure 7B).

We then compared the frequency of randomly monoallelic genes in *Ccnd3*^*−/−*^ pro-B cells to that in WT pro-B cells (10,000 genes) using the criteria above (Figure 7D, left) or a more stringent criteria, in which monoallelic expression must occur in at least ten tissues (Figure 7D, right). With either criterion, cyclin D3 preferentially repressed randomly monoallelic expressed genes in pro-B cells. As was observed for the V_κ_ genes, cyclin-D3-repressed genes were not appreciably marked with H3K27me3 (Figures 7E and 7F). These data suggest that non-epigenetic cyclin-D3-mediated repression is a general mechanism linking cell cycle exit to random monoallelic expression.

## DISCUSSION

For V_κ_-J_κ_ recombination, V_κ_ genes must be made accessible through mechanisms that both ensure developmental-stage-specific recombination yet allow diverse use of functional V_κ_ gene segments arrayed over approximately 3 mb. Herein, we demonstrate that V_κ_-gene-containing TADs are stochastically captured by transcription factories that then track and transcribe over long distances to open multiple V_κ_ genes. In each cell, different TADs can be stochastically captured by different transcription factories and transcription initiated at one of multiple E2A-bound promoters or CTCF sites (Figure 7G). In this way, unique stochastic repertoires of V_κ_ genes are transcribed in each cell. We propose that these transcribed V_κ_ genes define the repertoire available for recombination to J_κ_ (Yancopoulos and Alt, 1985). In any one cell, V_κ_ transcription is initiated primarily at one allele and transcription is repressed by cyclin D3. Therefore, our studies provide a mechanism for understanding several important features of *Ig*_*κ*_ recombination, including the generation of V_κ_ diversity, monoallelic choice, and the restriction of recombination to non-dividing cells.

Capture of genes by fixed RNAP factories has been observed for other genes, including c-myc (Osborne et al., 2007), and other clustered genes, including Igh (Park et al., 2014), protocadherin genes (Guo et al., 2012), and olfactory receptor genes (Clowney et al., 2012). Indeed, it has been proposed to be the most common mechanism of transcriptional activation (Papantonis and Cook, 2013). Stochastic chromatin loop capture provides a mechanism by which Vκ genes, arrayed over large genomic distances, could be coordinately and stochastically expressed in individual cells.

Transcription of Vκ genes tended to be initiated at Vκ promoters, especially those known to be pre-bound by E2A (Lin et al., 2010). However, we also observed a strong preference for transcribing Vκ genes that were proximate to, and oriented away from, CTCF-bound sites. This is consistent with chromatin loops being captured at or near CTCF-bound sites with only transcription away from these sites being productive. This is not unexpected, as it is known that RNAP is recruited to CTCF sites (Chernukhin et al., 2007). Furthermore, two anchor mechanisms for initiating transcription is consistent with the pattern of Vh usage observed in the primary Igh repertoire (Bolland et al., 2016), suggesting a similar underlying mechanism.

Previous studies have relied on bulk cultured pre-B cells or pre-B cell clones from F1 (C57BL/6 × CAST/EiJ) mice, in which *Ig*_*κ*_ recombination is induced by withdrawing IL-7 or by shifting temperatures in a sensitive A-MuLV cell line (Farago et al., 2012; Levin-Klein et al., 2017). These experimental approaches have significant limitations. Most notably, cell cycle exit and *Ig*_*κ*_ recombination progresses asynchronously in these cell populations, and therefore, it is difficult to differentiate mechanisms of initial allelic choice from those that reinforce allelic choice. Our findings make it likely that epigenetic changes observed at the V_κ_ genes in cell lines reinforce allelic decisions and do not play a role in allelic choice (Farago et al., 2012; Levin-Klein et al., 2017). Likewise, the recent observation that V_κ_ gene transcription is biallelic in cell lines (Levin-Klein et al., 2017) likely reflects expression in a heterogeneous cell population. Another limitation of most cell line experiments is a lack of controls relevant to normal lymphopoiesis. Whereas in F1 cell line populations, the *Ig*_*κ*_ alleles can be compared, the presence or magnitude of observed differences in relevant primary cells remains largely unexplored.

Cyclin-D3-mediated repression of V_κ_ transcription is independent of its role in cycle progression (Powers et al., 2012). Different domains of cyclin D3 mediate cell cycle progression and V_κ_ repression, as inhibition of CDK4/6, dampens cell cycle progression, but does not induce V_κ_ transcription (Powers et al., 2012). Furthermore, V_κ_ repression is mediated by the large fraction of cyclin D3 bound to the nuclear matrix and not available to productively bind CDK4/6. Fundamentally, we do not understand how genes translocate to transcription factories, and therefore, it is difficult to postulate how cyclin D3 might regulate this process. However, our data identify a specific mechanism regulating gene activation by transcription factories.

Our observation that elongating RNAP can track over long genomic distances is similar to the behavior of the RAG proteins, which scan for recombination signal sequences (Hu et al., 2015). Both mechanisms are constrained by CTCF-bound sites and therefore both function within TADs. Together, these two sequential loop capture events, first by NM-RNAP and then by RAG1/2 (Hu et al., 2015), are predicted to shape repertoire and lead to monogenic V_κ_-J_κ_ recombination.

Other V genes, including *Igh* and *Tcrγ* V genes, were de-repressed in *Ccnd3*^*−/−*^ pro-B cells. This suggests that monoallelic choice at these loci might also be determined by V accessibility. More broadly, cyclin D3 predominantly repressed monoallelically expressed genes, including members of the olfactory and protocadherin gene families. Olfactory receptors are encoded by a very diverse family of receptor genes (approximately 1,400 in mice; Monahan and Lomvardas, 2015) that are expressed monoallelically and monogenically in terminally differentiated olfactory neurons. Like the V_κ_ genes, multiple olfactory genes can be transcribed in single immature neurons prior to terminal maturation and the choice of a single gene (Hanchate et al., 2015). Also, similar to antigen receptor genes, protocadherin and olfactory gene segments are clustered within topological domains (Guo et al., 2012; Monahan and Lomvardas, 2015). These data suggest that TAD capture transcription, and its regulation by cyclin D3, is a general mechanism of monogenic choice among clustered gene families.

## EXPERIMENTAL PROCEDURES

### Mice

WT (C57BL/6), *Ccnd3*^*−/−*^ (C57BL/6), *Rag2*^*−/−*^ (Balb/C), and C57BL/6 × CAST/EiJ mice were housed in clean animal facility at University of Chicago and used at 6–12 weeks of age under Institutional Animal Care and Use Committee (IACUC) protocol.

### Isolation and Culture or Sorting of WT, *Ccnd3*^*−/−*^ Pro-B, and *Rag2*^*−/−*^ Pro-B Cells

Pro-B cells were isolated by positive selection (CD19^+^) for *Rag2*^*−/−*^ pro-B cells or by negative selection (CD3^-^CD4^-^CD8^-^Ter-119^-^IgM^-^CD11b^-^CD11c^-^Gr1^-^NK1.1^-^) for WT and *Ccnd3*^*−/−*^ mice using magnetic-activated cell sorting (MACs) columns and culturing them in 12 ng/μL of IL-7 for 2 days. Alternatively, cells were fluorescence-activated cell sorting (FACS) sorted for pro-B cells (CD19^+^B220^+^IgM^-^CD43^+^) and small pre-B cells (CD19^+^B220^+^IgM^-^CD43^-^small).

### *In Situ* Hybridization

V_κ_ (RP-23 182E6), C_κ_ (RP-24 387E13), and Vβ (RP-23 184C1) BACs (Children’s Hospital Oakland Research Institute [CHORI]) were labeled using nick translation. The V_κ_ RNA probe (V_κ_1–117) bound between heptamer recombination signal sequence (RSS) and 3′ UTR of V_κ_1–117 (Table S3), and the J_κ_ RNA probe bound 5′ of J_κ_1 (Table S3; Affymetrix). RNA probes were used with ViewRNA ISH kit (Affymetrix; QVC0001).

### Combined Immuno DNA-RNA FISH

Cultured or FACS-sorted pro-B (B220^+^CD19^+^IgM^-^CD43^+^) and small pre-B (B220^+^CD19^+^IgM^-^CD43^-^) cells were plated on poly-L lysine and washed on ice with CSK buffer with 0.5% Triton (Sawasdichai et al., 2010). For DNA FISH plus IF, cells were fixed with 2% paraformaldehyde (PFA), treated with 0.2 μg/mL RNase for 30 min in 37°C followed by 0.7% Triton/0.1 M HCL for 10 min on ice, denatured in 50% formamide/2× saline sodium citrate (SSC) for 10 min in 80°C, hybridized to probes at 37°C for 16 hr, and washed, blocked, and stained the next day (Chaumeil et al., 2013). Samples were stained for pSer2 RNA Pol II (clone 3E10; EMD Millipore 04–1571) and cyclin D3 (Cell Signaling Technology; DCS22 mAb 2936) and mounted in Prolong Gold (Molecular Probe P36930). For DNA-RNA FISH, the RNase step was omitted, and after DNA-FISH and IF steps, samples were incubated at 40°C with RNA probe followed by pre-amplification mix, amplification mix, and finally labeled probe (as described by the manufacturer; ViewRNA ISH Assay Affymetrix; QVC0001).

### Combined Immuno RNA FISH

For RNA FISH using distal (488-labeled) and proximal RNA probe (647-labeled) cocktails, probes were custom made by Affymetrix (Table S1). FACS-sorted CD43^low^ small pre-B cells (B220^+^CD19^+^IgM^-^CD43^low^) were plated on poly-L-lysine-coated coverslips, fixed, and permeabilized. After immuno-staining for pSer2 RNA Pol II, cells were hybridized with RNA probes using manufacturer’s protocol (ViewRNA ISH Assay Affymetrix; QVC0001). Samples were mounted in Prolong Gold for imaging.

### Imaging

Images were captured with a Leica TCS SP5 II STED laser scanning confocal microscope (Leica Microsystems), and image processing was performed using ImageJ. For super-resolution imaging, samples were mounted in Prolong Gold and images captured with a Leica SR GSD 3D Ground State Depletion Microscope 3 days post-mounting. Alexa Fluor 647 was depleted with 15% laser (approximately 23 mW) and acquired at threshold of 10 events with 5% laser and 10% back-pump (approximately 2 mW of 405-nm laser power). Alexa Fluor 532 was depleted with 100% laser and acquired with threshold of 20 with 20% laser.

### ICeChIP

#### Preparation of Chromatin

Pro-B cells from *Rag2*^*−/−*^ bone marrow were cultured in complete Opti-MEM supplemented with IL-7 (10 ng/mL). After one week, 65 million cells were iso-lated and nuclei prepared for ICeChIP as described (Grzybowski et al., 2015). Next, semisynthetic nucleosome standards for H3K27me3, H3K4me3, H3K9me3, H3K36me3, and H3K79me2 were “spiked in.” Native chromatin (containing standards) was digested with MNase (Worthington). Chromatin was released from nuclei with 0.6 M NaCl, centrifuged, and soluble chromatin (supernatant) collected. Mononucleosomes were purified from the clarified fragmented chromatin extract using hydroxyapartite (HAP) resin (Bio-Rad Ceramic HAP type I 20 μm) and Millipore Ultrafree MC-HV Centrifugal Filters (0.45 μm).

Semisynthetic nucleosome standards were generated by refolding (at equal molar) recombinant core histones (H2A, H2B, and H4) with semisynthetic histones (H3) consisting of a recombinant H3 protein harboring the indicated synthetic histone modification. Purified histones were then mixed with barcoded ladder DNA identifiable by sequencing.

H3K27me3 ChIP was performed with 10 μg of chromatin and the remainder used as input control (Grzybowski et al., 2015). Protein A Dynabeads conjugated to H3K27me3-specific antibodies (CST C36B11) were incubated with chromatin purified and spiked as above for 10 min at 4°C. Beads were washed as described (Grzybowski et al., 2015), samples eluted, and both samples and input treated with RNase A. Proteins were digested with proteinase K and DNA recovered using a Qiaquick DNA Purification kit (QIAGEN).

### Data Preparation and Analysis

Paired-end DNA libraries were prepared from input and immunoprecipitation (IP) samples followed by sequencing on an Illumina NextSeq500 by the University of Chicago Functional Genomics Core facility. Data were prepared by the following pipeline (Grzybowski et al., 2015). Raw FastQ reads were run through FastQ Grommer and then aligned with Bowtie alignment software (sensitive preset option; end-to-end alignment) to the mm9 reference genome (mm9_NCBI_build_37.1) concatenated with semisynthetic nucleosome standard barcode sequences. Reads that were unpaired, unmapped, and in wrong pair were filtered out using SAMtools. Low-quality reads (MAPing Quality [MAPQ] score < 20) were also removed. High-quality paired-end reads were flattened into single entries, and reads longer than 220 bp were removed to exclude fragments larger than mononucleosomes. Resulting files were then converted to genome coverage bedgraphs using BEDTools.

IP specificity and HMD were determined with the following equations (Grzybowski et al., 2015):

$$Barcode\;P\;enrichment=\frac{\sum_{1}^{N}IP}{\sum_{1}^{N}input};$$

$$HMD\;\left(per/bp\right)=\frac{\frac{IP}{input}*100\%}{Barcode\;IP\;enrichment}.$$

Barcode enrichment corresponds to the number of barcode reads for each semisynthetic nucleosome standard in the IP compared to the input sample. This information was then used to calculate HMD. BEDtools MapBed was used to calculate the average H3K27me3 HMD over gene regions. For meta-analysis of HMD and accessibility (ATAC-seq), cushions corresponding to 10% of each gene’s length were added to the beginning and end of each gene. Annotate peaks (Homer software) was then used to generate 100 bins for each gene and then calculate the average HMD for each percentage of the gene region. Accessibility data were further analyzed focusing on the region −10% to +10% corresponding to the transcriptional start site.

### Single-Cell RNA-Seq of B6XCAST F1

CD19^+^B220^+^IgM^-^CD43^low^ small pre-B cells were bulk sorted and then single cell sorted on pre-primed C1 Fluidic Chips. Cells were lysed, RT performed (Clontech SMARTer), and cDNAs transferred to 96-well plates. Libraries were prepared using Nextera XT (Illumina) and then tagmented. Pooled libraries were purified with AMPure magnetic separation assayed for quality with Qubit dsDNA HS (Life Technologies) and sequenced (75 bp paired). Raw fastq data were quality trimmed to a minimum Phred score of 20 using trimmomatic. Reads were then filtered against mouse ribosomal sequences using bowtie2, followed by full genome and transcriptome alignment to mouse reference mm10 using STAR. Apparent PCR duplicates and unassignable reads were removed using Picard Mark Duplicates (http://broadinstitute. github.io/picard). SNPs for CAST/EiJ mice were obtained from release1505-GRCm38 from Sanger Institute’s ftp site (ftp://ftp-mouse.sanger.ac.uk/). SNPs with quality score of at least 100, reported by Sanger, were retained for further analysis. A coverage threshold of 30 per SNP per expressed V_κ_ segment was used for allelic assignment.

Unrearranged cells were discriminated by biallelic expression of distal 5′J_κ_1 promoter and/or proximal promoter (J_κ_p2), presence of heptamer and nonamer RSS motif in the J segment reads, and absence of recombination products. Read alignments were split into separate bed regions based on their CIGAR assignment for splicing (N in CIGAR strings) using bedtools bamtobed. V segments annotated using bedtools were counted to get V-V splicing. Spliced V_κ_ genes were interpolated and used for the analysis. Splice junction sequences were consistent with published literature (Padgett, 2012). Only segment-to-segment splicing that was observed at least 10 times was reported.

### Monoallelic Gene Expression Database

Gene ids of 62 cyclin-D3-repressed genes were entered on dbMAE database (https://mae.hms.harvard.edu/) and tested for random monoallelic expression in multiple tissues. Allele-specific expression data from multiple tissues compiled from various studies were used to assess random monoallelic expression (Savova et al., 2016).

## Supplementary Material

1

2

## Highlights

- Prior to *Ig*_*κ*_ recombination, each pre-B cell monoallelically transcribes multiple V_κ_s
- V_κ_ containing chromatin loops translocate to fixed RNA Pol II transcription centers
- V_κ_ translocation and transcription is repressed by nuclear-matrix-bound cyclin D3
- V_κ_ transcription initiates near CTCF and E2A-bound sites
