# Supplementary material for: Regulated Capture of Vκ Gene Topologically Associating Domains by Transcription Factories
Source: Cell Rep. Author manuscript; Available in PMC 2018 Dec 28. (PMC6310487; doi:10.1016/j.celrep.2018.07.091)
Supplement: 1 [file NIHMS1506162-supplement-1.pdf]

Cell Reports, Volume *24*

Supplemental Information

Regulated Capture of Vκ Gene Topologically Associating Domains by Transcription Factories

Sophiya Karki, Domenick E. Kennedy, Kaitlin Mclean, Adrian T. Grzybowski, Mark Maienschein-Cline, Shiladitya Banerjee, Heping Xu, Elizabeth Davis, Malay Mandal, Christine Labno, Sarah E. Powers, Michelle M. Le Beau, Aaron R. Dinner, Harinder Singh, Alexander J. Ruthenburg, and Marcus R. Clark

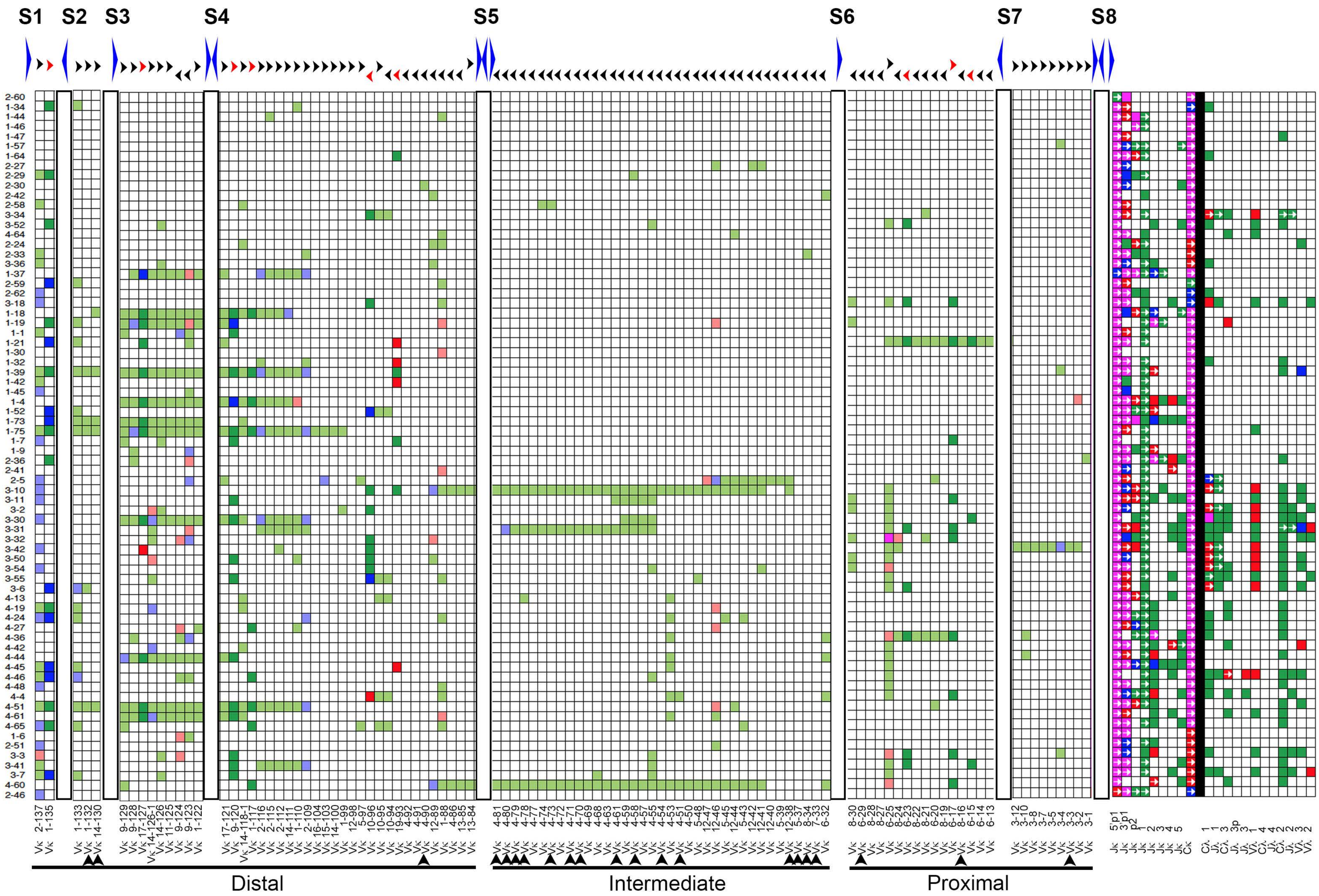
S1
S2
S3
S4
S5
S6
S7
S8
Distal
Intermediate
Proximal

Figure S1 Related to Figures 1 and 2. Single Cell RNA-seq Heatmap of *Igκ* and *Igλ* Expression.

The heatmap shows 72 analyzable cells in germline configuration, with Vκs expressed from B6 shown in red, CAST shown in blue and unassigned shown in green. High usage Vκs are shown as dark red (B6), dark blue (CAST) and dark green (unassigned). Biallelic expression of Vκ or Jκ segments is shown as magenta. All Jκ and Cκ segments are shown as dark red, blue and green for B6, CAST and unassigned respectively. Only 105 of 162 Vκ are shown, which includes 93 that have been evaluated to be functional [S1] and 12 that showed expression in 3 or more cells. Tracks of sequential Vκ expression represent those Vκs, which had undergone splicing between the first, and the last Vκ in the series and intervening Vκs were interpolated. CTCF location and orientation are shown as blue arrows on the top. Only peaks with strong binding and high contact probability (see experimental procedures) are shown [S2][S3]. Individual CTCF sites are numbered S1-S8. Smaller black arrows on the top represent transcriptional orientation of each Vκ, with red highlighting orientation of high used Vκ. Solid arrow heads at bottom denote poorly used Vκ genes.

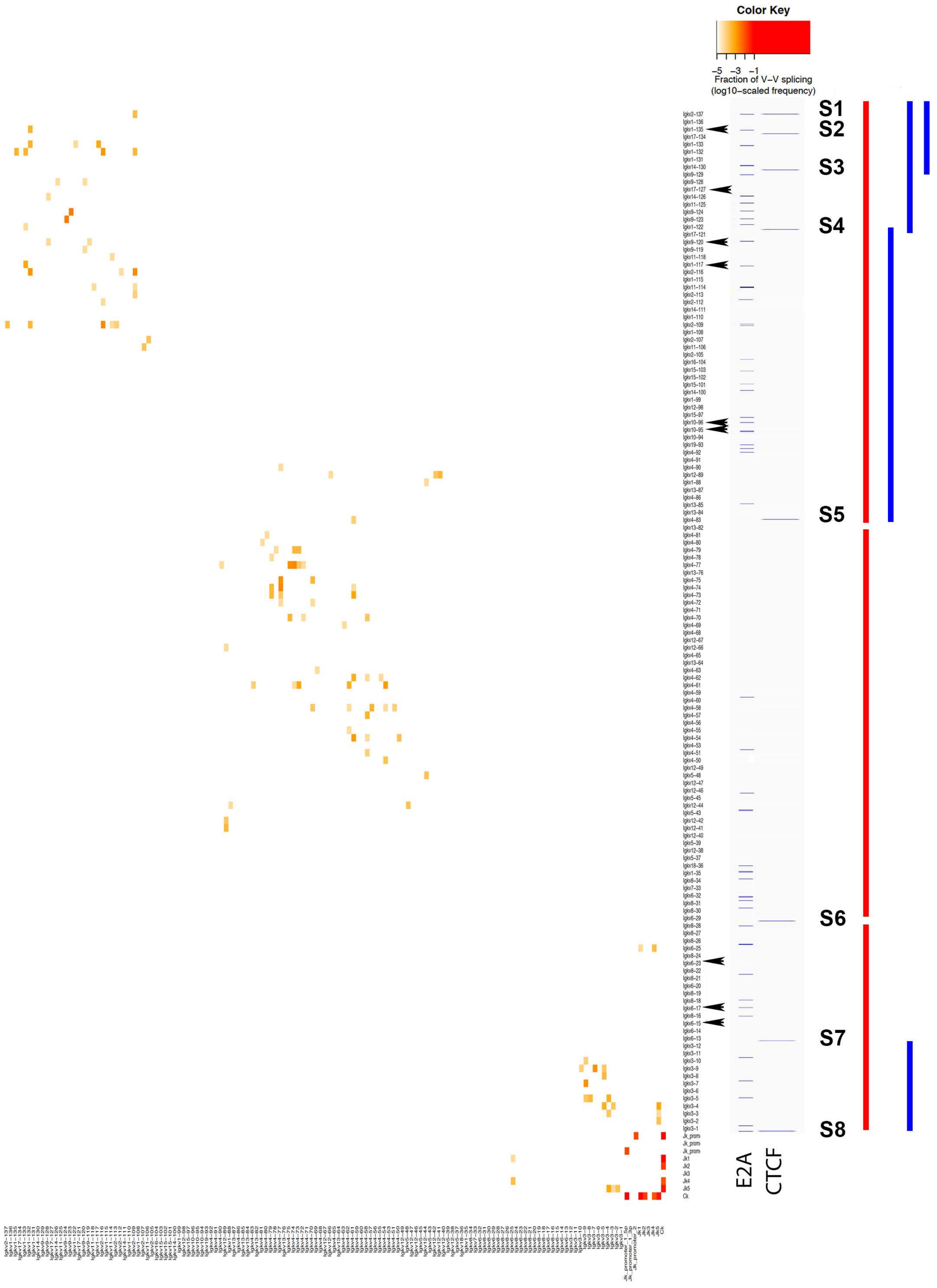

Color Key
−5 −3 −1
Fraction of V−V splicing
(log10−scaled frequency)
S1
S2
S3
S4
S5
S6
S7
S8
E2A
CTCF

Figure S2 Related to Figure 2. Heatmap of Vκ Spliced over *Igκ* Locus.

All spliced Vκs (colored) and unspliced Vκs (white space) shown. Highly used Vκs shown as black arrow. Bed files comparing CTCF peaks and E2A peaks from *Rag2*$^{-/-}$pro-B cells [S3] Individual CTCF sites are numbered as S1-S8. Red and blue lines represent TADs and sub-TADs, respectively as predicted from CTCF sites.

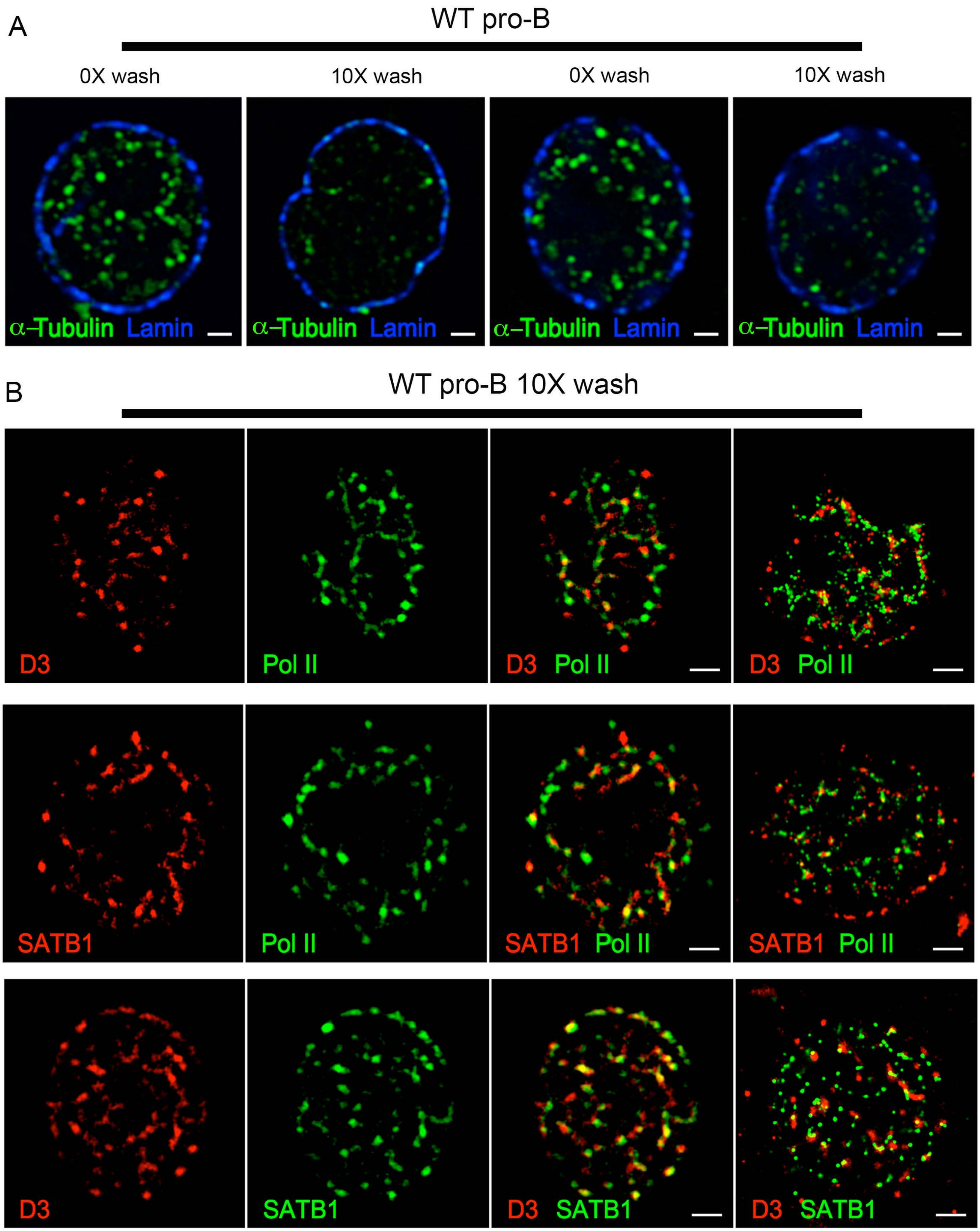

A
WT pro-B
0X wash
10X wash
0X wash
10X wash
α–Tubulin Lamin
α–Tubulin Lamin
α–Tubulin Lamin
α–Tubulin Lamin
B
WT pro-B 10X wash
D3
Pol II
D3 Pol II
D3 Pol II
SATB1
Pol II
SATB1 Pol II
SATB1 Pol II
D3
SATB1
D3 SATB1
D3 SATB1

Figure S3 Related to Figure 4. Assembly of cyclin D3 and RNAP on SATB1^{+} Nuclear Matrix.

(**A**) Representative confocal images of WT pro-B cells (40 cells, n=2 experiments) without CSK+0.5% triton washing, fixed and stained for the soluble nuclear protein α−tubulin and lamin (panel 1 and 3). Confocal images of WT pro-B cells washed 10 times (3 minutes/wash) with CSK+0.5% triton (panel 2 and 4). Scale bar: 1μm.

(**B**) Representative confocal images of WT pro-B cells (40 cells, n=2 experiments) washed 10X (3' each) with CSK+0.5% triton buffer and fixed and stained for cyclin D3/ RNAP, SATB1/ RNAP and SATB1/cyclin D3 (panel 1-3). Super-resolution image of WT pro-B cells washed and stained with cyclin D3/ RNAP, SATB1/ RNAP and SATB1/cyclin D3 (panel 4). Scale bar: 1μm.

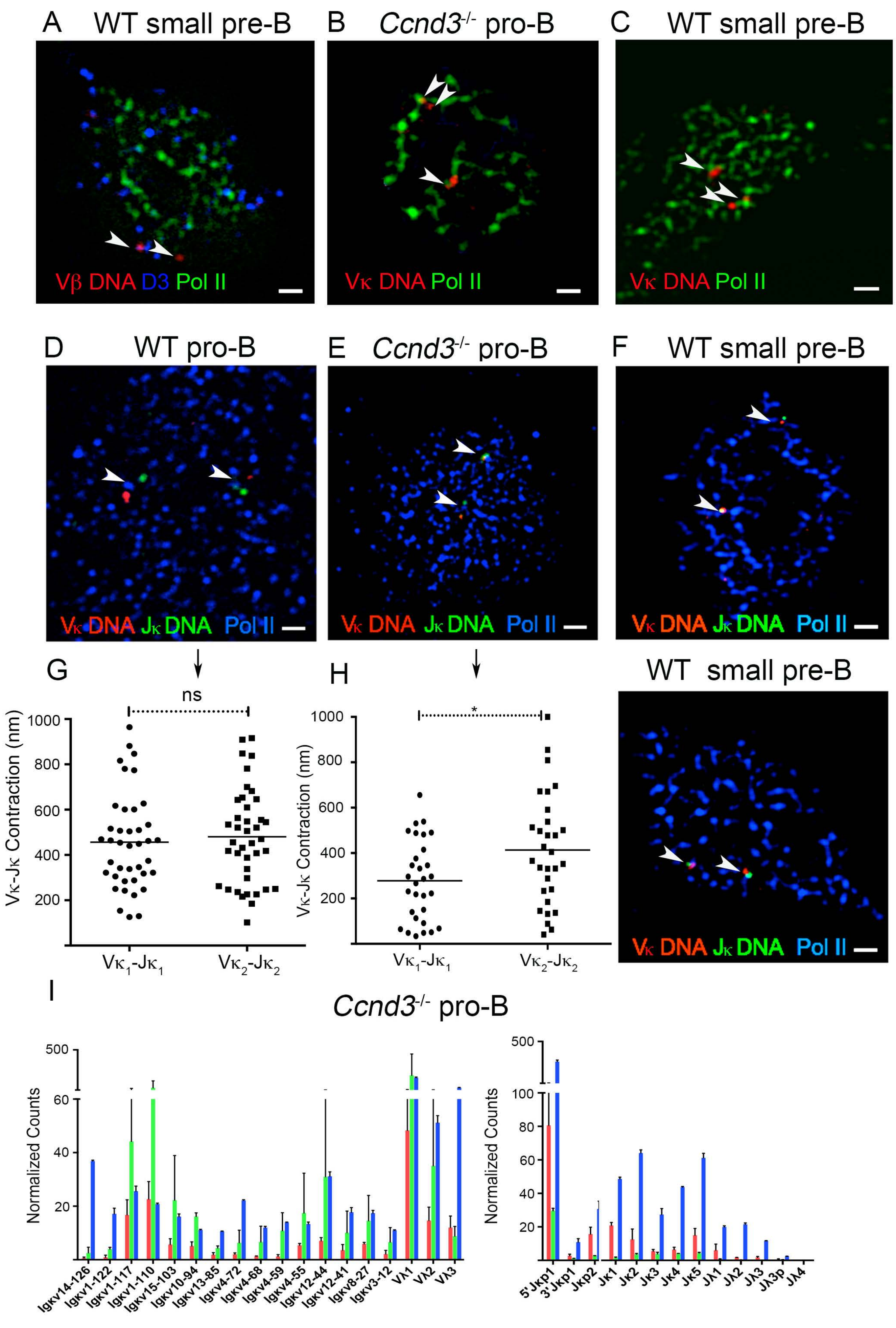

A WT small pre-B
Vβ DNA D3 Pol II
B Ccnd3-/- pro-B
Vκ DNA Pol II
C WT small pre-B
Vκ DNA Pol II
D WT pro-B
Vκ DNA Jκ DNA Pol II
E Ccnd3-/- pro-B
Vκ DNA Jκ DNA Pol II
F WT small pre-B
Vκ DNA Jκ DNA Pol II
G
ns
Vκ-Jκ Contraction (nm)
1000
800
600
400
200
Vκ1-Jκ1
Vκ2-Jκ2
H
*
Vκ-Jκ Contraction (nm)
1000
800
600
400
200
Vκ1-Jκ1
Vκ2-Jκ2
WT small pre-B
Vκ DNA Jκ DNA Pol II
I
Ccnd3-/- pro-B
Normalized Counts
500
60
40
20
Igκv14-126
Igκv1-122
Igκv1-117
Igκv1-110
Igκv15-103
Igκv10-94
Igκv13-85
Igκv4-72
Igκv4-68
Igκv4-59
Igκv4-55
Igκv12-44
Igκv12-41
Igκv8-27
Igκv3-12
Vλ1
Vλ2
Vλ3
Normalized Counts
500
100
80
60
40
20
5'Jκp1
3'Jκp1
Jκp2
Jκ1
Jκ2
Jκ3
Jκ4
Jκ5
Jλ1
Jλ2
Jλ3
Jλ3p
Jλ4

Figure S4 Related to Figure 5. Vκ, not TCR Vβ, Co-localizes to NM-RNAP and Transcribing Vκ Replicates Early and Contracts Preferentially.

(**A**) Representative confocal images of WT small pre-B cells (50 cells, n=3 experiments) washed with CSK+0.5% Triton buffer, hybridized with TCR Vβ DNA probe (RP23-184C1) and stained with cyclin D3 and RNAP. Arrows show individual alleles. Scale bar: 1μm.

(**B-C**) Representative confocal images of *Ccnd3*$^{-/-}$ pro-B cells (50 cells, n=3 experiments) and WT small pre-B cells and washed with CSK+0.5% Triton buffer, hybridized with Vκ DNA probe (RP23-182E6: top) spanning 10 distal Vκ genes, and stained with e-Pol II. Arrows show individual alleles. Double arrow show asynchrony in replication. Scale bar: 1μm.

(**D-F**) Representative confocal images of WT pro-B cells, WT small pre-B cells and *Ccnd3*$^{-/-}$ pro-B cells (40 cells, n=2 experiments), washed with CSK+0.5%Triton, hybridized to DNA probe RP23-182E6 (Vκ: red) and RP24-382E13 (Jκ: green) and stained for e-Pol II (blue). Arrows show individual alleles. Scale bar: 1μm.

(**G**) Minimum distances between Vκ and Jκ, plotted for alleles <200nm (Vκ1-Jκ1) from RNAP and >200nm (Vκ2-Jκ2) from RNAP using Euclidean Distance Transformation on Imaris. Analysis performed on same images used in (D). Statistical significance calculated on=37 by paired Student's t test (p<0.05 *, p<0.01 ** and p<0.001 ***).

(**H**) Minimum distances between Vκ and Jκ, plotted for alleles <200nm (Vκ1-Jκ1) from RNAP and >200nm (Vκ2-Jκ2) from RNAP using Euclidean Distance Transformation on Imaris. Analysis performed on same images used in (E). Statistical significance calculated on=37 by paired Student's t test (p<0.05 *, p<0.01 ** and p<0.001 ***).

(**I**) *Ccnd3*$^{-/-}$ pro-B RNA-seq normalized read counts, showing expression of multiple Vκ and Vλ gene segments (first panel) and repression of Jκ-Cκ and Jλ-Cλ (second panel). Red bar denotes expression in WT pro-B cells, green denotes expression in *Ccnd3*$^{-/-}$ pro-B cells and blue denotes expression in WT small pre-B cells.

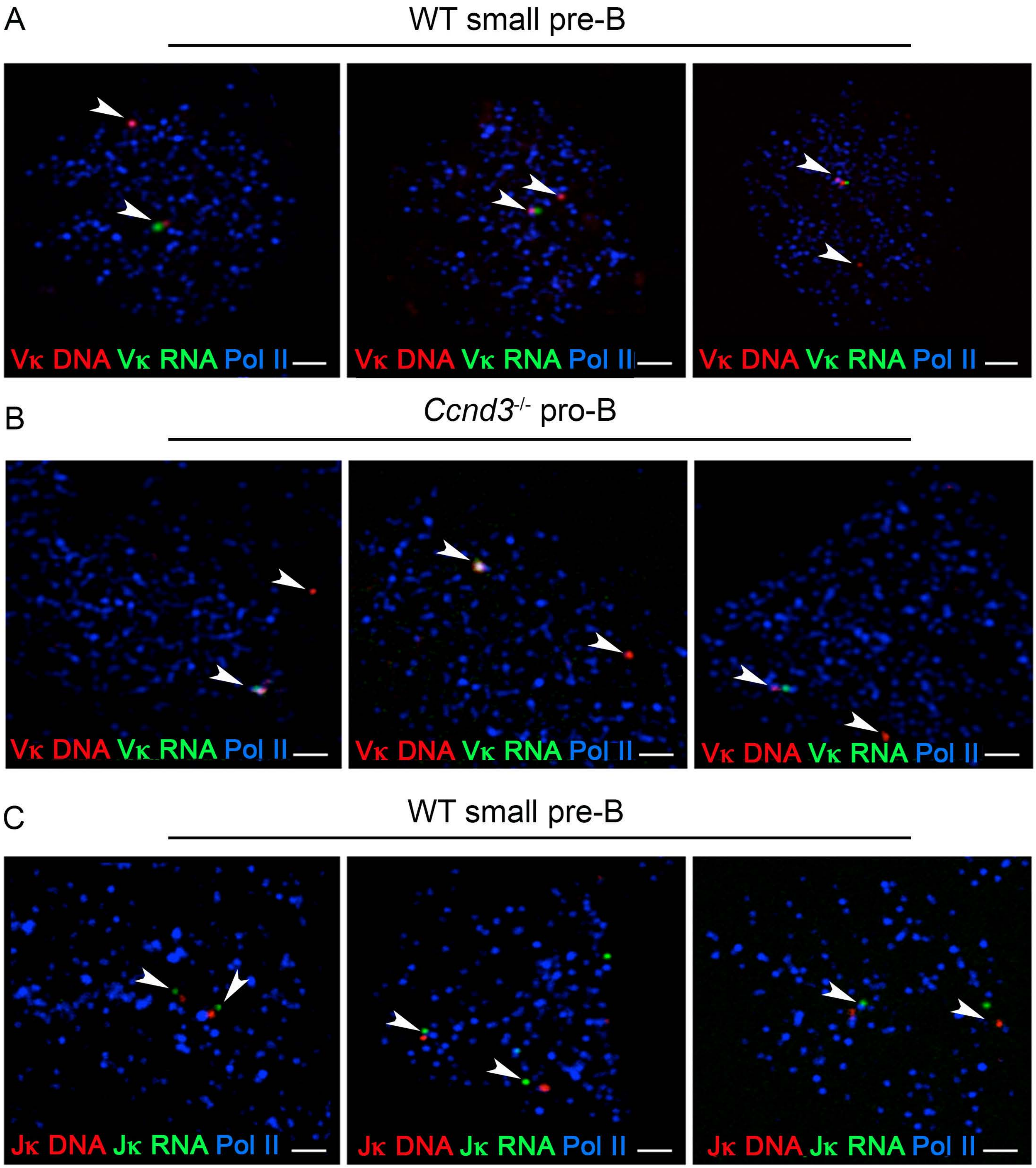
A
WT small pre-B
Vκ DNA Vκ RNA Pol II
Vκ DNA Vκ RNA Pol II
Vκ DNA Vκ RNA Pol II
B
Ccnd3-/- pro-B
Vκ DNA Vκ RNA Pol II
Vκ DNA Vκ RNA Pol II
Vκ DNA Vκ RNA Pol II
C
WT small pre-B
Jκ DNA Jκ RNA Pol II
Jκ DNA Jκ RNA Pol II
Jκ DNA Jκ RNA Pol II

Figure S5 Related to Figure 6. Mono-allelic Vκ Transcription upon Loss of cyclin D3.

(**A**) Representative confocal images of WT small pre-B cells (40 cells, n=2 experiments), washed with CSK+0.5%Triton, hybridized to Vκ DNA probe RP23-182E6 and RNA probe targeting Vκ 1-117, and stained for RNAP, shown horizontally from two independent experiments. Arrows show individual alleles. Scale bar: 1μm.

(**B**) Representative confocal images of *Ccnd3*$^{-/-}$ pro-B cells (40 cells, n=2 experiments), washed with CSK+0.5%Triton, hybridized to Vκ DNA probe RP23-182E6 and RNA probe targeting Vκ 1-117, and stained for RNAP, shown horizontally from two independent experiments. Arrows show individual alleles. Scale bar: 1μm.

(**C**) Representative confocal images of WT small pre-B cells (40 cells, n=2 experiments), washed with CSK+0.5%Triton, hybridized to Jκ DNA probe RP24-387E13 and RNA probe targeting GL Jκ and stained for RNAP, shown horizontally from two independent experiments. Arrows show individual alleles. Scale bar: 1μm.

>Type4
tcagcagagtggaggctgaggatttgggagtttattattgctggcaaggtacacattttcctcacacagtgattcagacc
cgaacaaaaacttccttgcctggggcagcccagctgacaatgtgcaatctggagaggagcagagagcatcttgtgtctgt
gtgagagggaggggctggggaacatgagtacttgttcacagctgtgagctctgttcagactcatatggctttatgagtgt
tttgactagatctcaagtgacatgaatttctggtccttcttgtaataggcattgtgttgcctctgcatcaggattatttc
cttttactaatctagctgatttttgatgatttaactattttattaattatttgagcttcatatttgttattcttaatga
agtatgtgtgtttgttaaattcattttcagattttaaattcttagatttgcatccatatggttaacatgcctctcacag
tgctatgtcctcttacagaaacctgcagatgctgtcagtgccgctacatcagctgcaattgaagctgttcagtgtgtgac
ctcatcaattgttggtttttattccttccatgcatatgcactctcacttcctttttcagtgtctggagcattcttggccc
cctattacttcctgaacccttcccttaagagagacagtaatagtttagatataattttccagggaagcctgtatcctgct
gcactgcagaggtactaagtcctgtccattggtacactcagttttacatttgagataaaatcaaagaatttgacttgtgc
tgaatctggtttaggtccatatataattcgttttgatattatctaatactaggaaatgagcattgtaattcaggaattat
tgatcagaagagatttaattttgtgagatgtttgtgagccatgtgtttaccaacttttgctcttaagaaactttagaaaa
atgattgctatagacttgaaaatatgcgattttattacagaaaaaaaaaaaaagcttccagaggggctgggcaaagtga
aagaggttgaaagaggaagacccagagaagagtcagaaagaattggactctcgcagatagagtggtgaaggattttgtct
caatgggttgtaagtattttgcaggggtgaattgtgtcagaataggaaggaagcaattattgttaagttggtggagacca
gtaggtaatcatttgggattaaggaagtattaggagatggtaggagatggtgaatattgagaaatcagaaagagacagtt
atgtgcacaggcccataaagcagtcagtcattttgtaaagctgtagcatgagagcggtgtggcttcatagtgactgagac
atattgacagccttggctccagggaacaatggaatctaatgaacagatagactgagtagctacatgaataagttaaacca
gccacttttaagaggtggtgcattctgtttaaaagtgagtctgaagattttgtagactattactgtctacaatatgctag
ttctcctcccacagtgatacaaatcataacataaaccccatgaaagcagaaatgagaggctgggctgctctagctgctcc
ttctcatgaatcacccactctgaatttctcagatggaacaggtctctctgagagtttggggtgactcctaagacacaaaa
tacacatatgcaatgcttctggaaggcaggagagcactacaattctgattactctaattccatacaaaagagaataataa
agttagaagatataatgccacctttctagggttccctagttatgctgtcctttgactgtctccataaattctagactgag
tttgccaactttcaaaactctttctcgaataccattttcattacactgagtttttttttcttttttttttttatcaaact
ggagatggagtttattactgctttcaaggttcacatgttcctcccacagtgatacagaccctaacaaaaatagtcttact
ttgggtatcacagctgctaaatatgttattcatgtttgagagaggttctgaggactccagtttattggttccagctgag
ggcaagtgcagcctggctacagctcactggtcattcatttttggcttcattgccacaataacaatatgaacaggtgccct
cccagaagccagaaaaaaatatttaaaaagctgagtgaaaacccatttagattcaaaaggcttgactgccttttcaatca
atcaatgttaaccagtaaagcagatggaaagccaagatatatttcttccttaaaatattttctatgttatatttcatttg
ccatattttatgttgttcactttaattcatatgtatcctttttttgaatttcaagttatagcaaaggattatggttaggcc
accttccttttctcagacaaatgtgatcaagctgttgtaagaaaaccaggaatcctatgtactgttccttttctcagtaa
tatttctctgccttttaaatagtagtgttttgtagctaattttcatcatttaaataatgttcactggaatttgcacagta
gcatccattcttctctgagtctccactgatgatacaactcaatcacttcaaaggaagcaactgcatcctttgttaaggta
gaaagggcattggtactcatgaaggggaaggaagataggtatacagagggctgtttagcccctcctaccaaacaaatga
ctcttcatgaacttagctaaaactcctttgacatctgtgatatagtttctgttggtgactcaccatgaggaacatctgga
gaagccaaaccccacacttctgctttcatggaggtttatgttatgacttatatcattgtgggaggaagcgtattaccctg
ttggcaaaagtaagtggcaatatcttcttgctccaggttgctattattattattactctgatgatgattggtgtactaat
aaaatttcaaatcttcattgtcataataaaaaagtgtattcctcagaaccttatcacattccacatatttgttgagcaac
agtatgataaaattgtaggttattacaacattatatgagttattaaaattataaacagaaacccaaaatcatggagtgca
gtatcttgaatttatatttatatctcaatttcctttgcaaaattcaagcatatatcagtatatctaaagcagtcatttg
atatttctcttttacacatttcacaccatttcttcttactgtttagtgcatgattttatttttaaatttattttgcaata
gcccagatttgtacatggaggtaaattcaactttattctcagaaataaaacctaaaaagaaatctgttaaaattaggccc
aaggagacttcactaatatcaattcttcatcactacaatatctaaaagcacattccccagagccttatttctttcaataa
gttcctgtagagcttatgaatgaagtgtccataagattaaagatcagagaattataatataactattgaaatattcaagt
aggactgtggatagaagtgatgactgaggcgatacaggcatggcaacacacgatcactcacatttttgtttttcctgaaca
catctgaggatcacatgatcaaatgatcagatagcctttccttctagtaaagatggatttgattgcagagagcttctgag
aataacatgttcctatatatacttaattgacgtcaggggtttaaaagctggaggtagatatgtttaaattactacagtac
tcacagctctttctctttctttctctctctgtttatgtatgtgtgcccacaaagaatctctgtaatcacataactctcaa
ttgatatacttcttaaactactcaagaaaaatgatttcatacattttgatcattgaaagcatagccatatttttttatcca
gagcttttttaaaagatagatgttttttattttttttttcttttttttttttcagttctctttttactaaaaggtcccaaagactt
tgcaacttagaaaaaaaaaatctcttagcaaaatggttaagacaaagggtaagaatttgtgggcaacctagcctctcactt
ctgcttccctgggtggtttttgttatgatttgtatcactgtgggtagaagattatcatactgtagacaataataagttgc
aatatcttcaggctccaggttg

>Type6
gctatttaaatagctatttactgtatcctagatttttatagattcataaaaccctgtgtgtgtgtgtactggtatgtgca
tgtgtgtgtgtgtgtgtgtgtgtgtgtgtgcatgtcagagggcacaactgtatgtcacatagaaaaaggtaaatttgttatg
ttcaatacacagaatttgagcatgagaattaaaaaaaataaagatttaatctctttacttctttttttccataaagacct
tccaaatcaaaggatcagttcgttaatttctaccaaactttgcaacacacaggcatagcacctgcaggcagaggggaag
tgtgtaggtctagggaggtgtagtgcaggcagctggttagaatctcagaggaggtttatgtaggaggctgaagcactgtg
agaggatagctgctatattggtttattactgtcagcaacattatagtactcctcccacagtgcttcagcctcctacacaa
acctccttgagagtttaaacatctgtgtacaccacacacagctctggttatgcacacttctccattcagacttagaggtg
ccatgtctgaaatgctgacgaaaaagtttacagagcccagtaaaaaataatgtgttcaatgcttcttatgtcttttggta
taaaacttttatacaccaaaatgcagggaaaaaactccacagtattgtttttctattccttctgcaacatattctgtaat
aaaaggctctgactggatttgcagctcatagtgaccttctgtcctactgacatagccagggaggatggagactgtgtcat
cacaatgtctgcacaggcacctgcaataattaataagcagtgaaaatgcatagattactgtatcctagatttttatagat
tcataaaaccctgtgtgtgtgtgtgtactggtatgtgcatgtgtgtgtatgtgtgtgtgtgtgtgtgtgtgtgtgtgtgt
gtgcatgtcagagggcacaactgtatgtcacatagaaaaaggtaaatttgttatgttcaatacacagaatctgagcatga
gaattaaaaaaaataaagatttaatctctttacttctttttgccattaaaacattccaagtcaaaggatctattcatta
atttctaccaaactttgcaacagacaggcatagcagctgcaggcagaggggaagtgtgtaggtctagggatgtgaggtg
caggcagctggttagaatctcagaggaggtttatgtaggaggctgaagtactgtgagaggatagctgttatattg

Table S1 Related to Figure 1. RNA Probe Sequences of Distal and Proximal V Cocktail used for RNA ImmunoFISH.

RNA probes were custom designed by Affymetrix, targeting the distal (Vκ 1-135, 17-127, 9-120, 1-117, 10-96, 19-93) and proximal (6-23, 6-17 and 6-15) V sequences. The target sequence for each V included RSS and 3'UTR to obtain probes that would be specific for germline transcripts. Probe sequences for distal probe cocktail (Type 4: 488 labeled) and proximal probe cocktail (Type 6: 647 labeled) are shown. The target sequences have been combined and probe sequences are shown in green. Probes were hybridized using ViewRNA ISH assay and immunofluorescence was performed (see experimental procedure).

List of genes used in this study

Vκ	Activated	H3K27me3 Repressed	Ccnd3 Repressed
Igkv2-137	Btg2	Cyp27b1	MYO1B
Igkv1-135	Vpreb3	Sp6	SPAG16
Igkv1-133	Tcf3	Srcin1	CYP27A1
Igkv1-132	Btg1	Ramp2	SPP2
Igkv14-130	Ikzf1	Galr2	RAB17
Igkv9-129	Ebf1	Tmem30b	CACNA1E
Igkv9-128	Ikzf3	Dnajc22	FCGR2B
Igkv17-127	Cd79b	Ntn3	SCML4
Igkv14-126-1	Fos	H2-T3	IGF1
Igkv14-126	Irf4	Lbx1	RMST
Igkv11-125	Syk	Pitx3	DDC
Igkv9-124	Il7r	Gm996	GRB10
Igkv9-123	Ccnd3	Lhx6	BCL11A
Igkv1-122	Rag1	Hrh3	TNFSF13
Igkv17-121	B2m	AI46131	SLC16A11
Igkv9-120	Cd93	Vax2os	CCL9
Igkv14-118-1	Lef1	Zfp580	APPBP2
Igkv1-117	Bach2	Dmpk	42982
Igkv2-116	Pax5	Hmx2	DGKE
Igkv1-115	Jun	Cilp2	NME1
Igkv2-112	Actb	Comp	PDK2
Igkv14-111	Ezh2	Foxf1	FBXL20
Igkv1-110	Gapdh	Isl2	GM9866
Igkv2-109	Cd79a		SNTG2
Igkv16-104	Spib		HECTD1
Igkv15-103	Cd19		NPAS3
Igkv14-100	Irf8		KLHDC1
Igkv1-99	Hprt		5830428M24RIK
Igkv12-98			4930447C04RIK
Igkv15-97			BCL11B
Igkv10-96			GPR132
Igkv10-95			GNG4
Igkv10-94			FAM65B
Igkv19-93			JARID2
Igkv4-92			4921525O09RIK
Igkv4-91			SLC6A19
Igkv4-90			SCAMP1
Igkv12-89			CRHBP
Igkv1-88			PLK2
Igkv4-86			SYNPO2L
Igkv13-85			ANXA8
Igkv13-84			OSGEP
Igkv4-81			RAB2B
Igkv4-80			PCDH9
Igkv4-79			ZIC2
Igkv4-78			NALCN
Igkv4-77			PRLR
Igkv4-74			FBXO32
Igkv4-73			GRINA
Igkv4-72			DNAJB7
Igkv4-71			HOXC8
Igkv4-70			LEPREL1
Igkv4-69			CLDN16
Igkv4-68			SEC22A
Igkv4-63			GBE1
Igkv4-61			2810055G20RIK
Igkv4-59			CBR1
Igkv4-58			TMEM181A
Igkv4-57			THBS2
Igkv4-55			GGNBP1
Igkv4-54			4930539E08RIK
Igkv4-53			PIM1
Igkv4-51			GLO1
Igkv4-50			H2-OB
Igkv5-48			MSH5
Igkv12-47			H2-Q1
Igkv12-46			EFNA5
Igkv5-45			LBH
Igkv12-44			EHD3
Igkv5-43			CRIM1
Igkv12-42			HAAO
Igkv12-41			FZD8
Igkv12-40			DSG1A
Igkv5-39			KLHL14
Igkv12-38			LRRTM2
Igkv5-37			PCDHB16
Igkv8-34			PCDHB20
Igkv7-33			CLCF1
Igkv6-32			CAPN1
Igkv8-30			TRPM3
Igkv6-29			SCD1
Igkv8-28			TCF7L2
Igkv8-27			CACNA1B
Igkv6-25			LHX3
Igkv8-24			PDK1
Igkv6-23			YPEL4
Igkv8-22			PRG3
Igkv8-21			COMMD9
Igkv6-20			FJX1
Igkv8-19			BMF
Igkv6-17			SEMA6D
Igkv8-16			NPHP1
Igkv6-15			PRNP
Igkv6-14			DUSP15
Igkv6-13			SULF2
Igkv3-12			SPO11
Igkv3-10			ZFHX4
Igkv3-8			1700034I23RIK
Igkv3-7			PFN2
Igkv3-5			DPM3
Igkv3-4			1700094D03RIK
Igkv3-3			SEMA6C
Igkv3-2			PDZK1
Igkv3-1			DRAM2

Vκ	Activated	H3K27me3 Repressed	Ccnd3 Repressed
			AGL
			CAMK2D
			ELTD1
			ABCA1
			E130308A19RIK
			RGS3
			SLC5A9
			PTPRF
			PPT1
			MTOR
			GCKR
			MXD4
			HTRA3
			AFAP1
			SLC2A9
			KLF3
			IGJ
			2010109A12RIK
			TGFBR3
			USP30
			2410018M08RIK
			PPP1R9A
			1700025E21RIK
			RAB11FIP5
			HDAC11
			NANOG
			CCND2
			ZFP93
			CEACAM2
			SPINT2
			SBSN
			ARRDC4
			CIB1
			FURIN
			9930013L23RIK
			2310010J17RIK
			SERPINH1
			FCHSD2
			OLFR65
			COQ7
			TMC7
			TMC5
			GPRC5B
			QPRT
			IFITM1
			EPS8L2
			KCNQ1OT1
			TNFRSF22
			MYOM2
			RBPMS
			GPR97
			4632415K11RIK
			RAB4A
			1700110K17RIK
			SCN4B
			IL10RA
			SEMA7A
			CYP11A1
			LIPC
			BCL2A1D
			BCL2A1A
			BCL2A1B
			RAB6B
			CISH
			TCTA
			ARPP21
			BCL2A1C
			VILL
			1700048O20RIK
			XLR
			3830403N18RIK
			PLXNB3
			CYSLTR1
			2900056M20RIK
			TMLHE
			EIF2S3Y
			UTY
			DDX3Y

Table S2 Related to Figure 3. List of genes used in H3K27me3 ICeChIP Analysis.

Vκ regions, activated genes, and repressed genes in pro-B cells.

Vκ 1-117 RNA probe	Affymetrix Product # VB6-17816
	ggtctgtatcactgtgggaggaa<br>gatacccaaagtaagactatttttgttag<br>aacatgaataacatatttagcagctgt<br>ggagtccctcagaacctctctca<br>ccctcagctggaaccaataaact<br>ctgtagccaggctgcacttg<br>gccaaaaatgaatgaccagtgag<br>ttcatattgttattgtggcaatgaa
Jκ RNA probe	Affymetrix Product # VB6-18544
	cctgtctaaactcccacataaatactc<br>acggtctctaggctcctgacc<br>tcttttaaagtacttttatttacttctatagttc<br>cctcccctacctcctcacctct<br>ttcccctttcatactgcatatca<br>ctctgcttgtacttttagtattccact<br>ctcatctccctgccttccac<br>tggacccaccaggctacaac<br>gaatgtagagattggctgaggtcta<br>tggggactgaagaaattgaagaa<br>tgtataactactgccagtgaagcc<br>gctctgggcaaaggttggt<br>aaacacttctgtccacacgcat<br>ggctgtggaaaccaccagag<br>gcatcaaggtattccagcgag<br>taacaaggcatggagcctaact<br>aaatatttttccacgcaggaa<br>cgatctgtacataagcttattaagctaaa<br>catgcaccctagcataagagaca<br>cactggattgtacaaaaatacaactg<br>aatctacagggagctctagcagtagt<br>gctaagtagaggaattaaatatgaagatc<br>ttgtccatagacttgtaaggttattgt<br>gctagctagtttgaagatgttttgtc<br>gatcattgaggaagaaggtggtaa<br>aattgaatggccttctttggata<br>atgtaatttggcatatgtgtgctag<br>ccagttttgttccccaggtg<br>tgtgcttggttgtttttaccatt<br>ccccatgtgtctaccactgacatat<br>tgaattctgagctctctgccttc<br>ccagcttactctatacgtgtgataaac<br>ttacatattggctgttgtgggtt<br>gaggggctaagcctctgaaat<br>gtggatcagggtttactaacctca<br>cacttcacatgaaagagaaaacagtaa<br>aggacattaggctacagccattt<br>gtgaagatgtgtctcttgatttcaga<br>aagtctggtcccagtctggaaa<br>gcatgtcacaacgtggcacaa<br>cctttcctgtgtgtgcatgtgt<br>catgtgggcacaagccaagt<br>cccacttgtccctgaagaaagg<br>tgagacgctccttatgtccatt<br>caaaggaggccacgtaagga

Table S3 Related to Figure 6. RNA Probe Sequences used for Combined RNA-DNA ImmunoFISH.

RNA probes were custom designed by Affymetrix from the target sequence provided (Vκ 1-117 RSS+3'UTR and Jκ 3.5kb upstream of Jκ1). Multiple short probes spanning the region of interest were obtained. Probe sequences for Vκ1-117 and Jκ are shown. Probes were hybridized using ViewRNA ISH assay in combination with DNA FISH and immunofluorescence (see experimental procedures).

SUPPLEMENTAL REFERENCES

[S1] Martinez-Jean, C., Folch, G., and Lefranc, M.P. (2001). Nomenclature and overview of the mouse (Mus musculus and Mus sp.) immunoglobulin kappa (IGK) genes. Experimental and clinical immunogenetics *18*, 255-279.

[S2] Choi, N.M., and Feeney, A.J. (2014). CTCF and ncRNA Regulate the Three-Dimensional Structure of Antigen Receptor Loci to Facilitate V(D)J Recombination. Front Immunol *5*, 49.

[S3] Lin, Y.C., Benner, C., Mansson, R., Heinz, S., Miyazaki, K., Miyazaki, M., Chandra, V., Bossen, C., Glass, C.K., and Murre, C. (2012). Global changes in the nuclear positioning of genes and intra- and interdomain genomic interactions that orchestrate B cell fate. Nat Immunol *13*, 1196-1205.
