# Supplementary material for: Regulated Capture of Vκ Gene Topologically Associating Domains by Transcription Factories
Source: Cell Rep. Author manuscript; Available in PMC 2018 Dec 28. (PMC6310487; doi:10.1016/j.celrep.2018.07.091)
Supplement: 2 [file NIHMS1506162-supplement-2.pdf]

Cell Reports

Article

Regulated Capture of Vκ Gene Topologically Associating Domains by Transcription Factories

Graphical Abstract

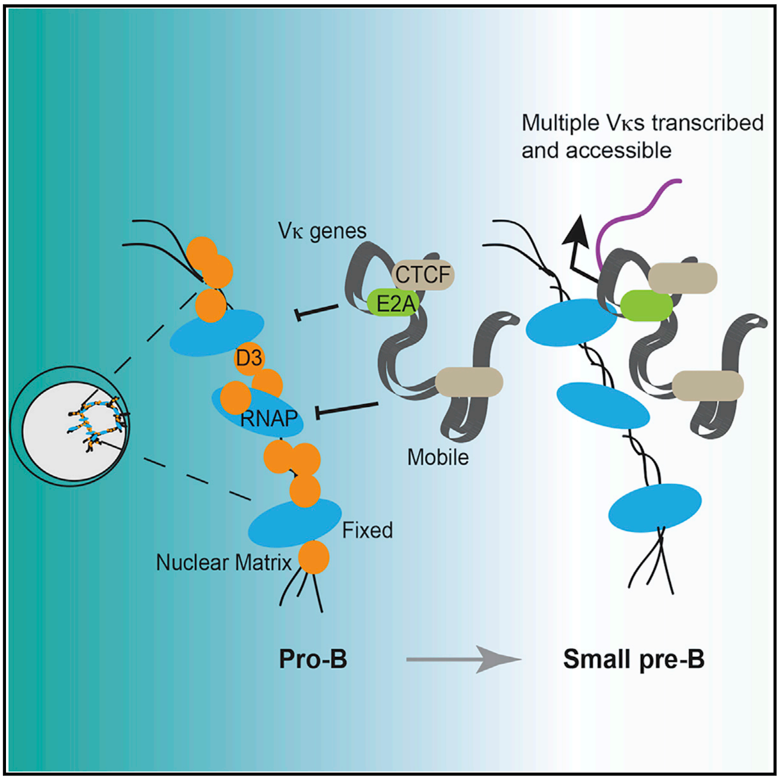

Authors

Sophiya Karki, Domenick E. Kennedy, Kaitlin Mclean, ..., Harinder Singh, Alexander J. Ruthenburg, Marcus R. Clark

Correspondence

mclark@uchicago.edu

In Brief

The mechanisms controlling Vκ transcription and their relationships to *Igκ* recombination are obscure. Karki et al. demonstrate that, upon translocation to transcription factories, Vκ-gene-containing chromatin loops are transcribed over long distances, which opens large, monoallelic, and diverse Vκ repertoires for subsequent Vκ-Jκ recombination.

Highlights

- Prior to *Igκ* recombination, each pre-B cell monoallelically transcribes multiple Vκs
- Vκ containing chromatin loops translocate to fixed RNA Pol II transcription centers
- Vκ translocation and transcription is repressed by nuclear-matrix-bound cyclin D3
- Vκ transcription initiates near CTCF and E2A-bound sites

Data and Software Availability

GSE84705

Karki et al., 2018, Cell Reports *24*, 2443–2456
August 28, 2018 © 2018 The Authors.
https://doi.org/10.1016/j.celrep.2018.07.091

CellPress

Cell Reports
Article

OPEN ACCESS
CellPress

Regulated Capture of Vκ Gene Topologically Associating Domains by Transcription Factories

Sophiya Karki,[1] Domenick E. Kennedy,[1] Kaitlin Mclean,[1] Adrian T. Grzybowski,[2] Mark Maienschein-Cline,[3] Shiladitya Banerjee,[4] Heping Xu,[5] Elizabeth Davis,[6] Malay Mandal,[1] Christine Labno,[7] Sarah E. Powers,[8] Michelle M. Le Beau,[6] Aaron R. Dinner,[9] Harinder Singh,[5] Alexander J. Ruthenburg,[2] and Marcus R. Clark[1,10,*]

[1]Department of Medicine, Section of Rheumatology and The Knapp Center for Lupus and Immunology Research, University of Chicago, Chicago, IL, USA
[2]Department of Molecular Genetics and Cell Biology and Department of Biochemistry and Molecular Biology, University of Chicago, Chicago, IL, USA
[3]Research Resource Center, University of Illinois at Chicago, Chicago, IL, USA
[4]Department of Physics & Astronomy, University College London, London WC1E6BT, UK
[5]Division of Immunobiology, Department of Pediatrics, University of Cincinnati, Cincinnati, OH, USA
[6]Section of Hematology/Oncology, University of Chicago, Chicago, IL, USA
[7]Integrated Light Microscopy Core Facility, University of Chicago, Chicago, IL, USA
[8]Department of Biology, Lewis University, Romeoville, IL, USA
[9]James Frank Institute, University of Chicago, Chicago, IL, USA
[10]Lead Contact
*Correspondence: mclark@uchicago.edu
https://doi.org/10.1016/j.celrep.2018.07.091

SUMMARY

Expression of vast repertoires of antigen receptors by lymphocytes, with each cell expressing a single receptor, requires stochastic activation of individual variable (V) genes for transcription and recombination. How this occurs remains unknown. Using single-cell RNA sequencing (scRNA-seq) and allelic variation, we show that individual pre-B cells monoallelically transcribe divergent arrays of Vκ genes, thereby opening stochastic repertoires for subsequent Vκ-Jκ recombination. Transcription occurs upon translocation of Vκ genes to RNA polymerase II arrayed on the nuclear matrix in transcription factories. Transcription is anchored by CTCF-bound sites or E2A-loaded Vκ promotors and continues over large genomic distances delimited only by topological associating domains (TADs). Prior to their monoallelic activation, Vκ loci are transcriptionally repressed by cyclin D3, which prevents capture of Vκ gene containing TADs by transcription factories. Cyclin D3 also represses protocadherin, olfactory, and other monoallelically expressed genes, suggesting a widely deployed mechanism for coupling monoallelic gene activation with cell cycle exit.

INTRODUCTION

Igκ is composed of variable (V) and joining (J) gene clusters that undergo monoallelic recombination following stochastic choice of single Vκ and Jκ genes. Recombination is spatiotemporally regulated by stage-specific accessibility of Vκ and Jκ gene clusters and expression of recombination-activating genes (RAGs) (Clark et al., 2014; Schatz and Ji, 2011). Both the Vκ and Jκ gene clusters are repressed in pro-B cells. The Jκ cluster is repressed by interleukin-7 (IL-7)-receptor-activated STAT5, which both drives proliferation and directly binds the Jκ cluster proximate enhancer, Eκi, and recruits the polycomb repressive complex (PRC2) that decorates the Jκ-Cκ region with H3K27me3 (Mandal et al., 2011). The choice of one *Igκ* allele for recombination has been correlated with monoallelic accumulation of activating histone marks in the Jκ cluster (Farago et al., 2012). However, these studies did not discriminate between deposition of histone marks prior to and after allelic choice and recombination. Furthermore, Jκ germline transcription (GLT) prior to recombination is biallelic (Amin et al., 2009), suggesting that Jκ accessibility does not determine allelic choice.

Whereas the Jκ cluster is less than 1 kb in length, the Vκ gene cluster stretches over approximately 3 mb and contains at least 93 (Martinez-Jean et al., 2001) functional and about 162 total Vκ genes organized into distal, intermediate, and proximal groups. Each group is defined by one or more topologically associating domains (TADs) formed by CCCTC-binding factor (CTCF)/cohesion complexes (Aoki-Ota et al., 2012; Lin et al., 2012; Ribeiro de Almeida et al., 2011). The Vκ-containing TADs contract onto the RAG-bound Jκ cluster, leading to Vκ-Jκ recombination (Schatz and Ji, 2011).

In contrast to the Jκ cluster, evidence that the Vκ genes are epigenetically repressed in early B cell progenitors is conflicting. In pro-B and large pre-B cells, qualitative chromatin immunoprecipitation followed by deep sequencing (ChIP-seq) indicates that the Vκ region is not substantially marked with H3K27me3 (Mandal et al., 2011; Xu and Feeney, 2009), while in cell lines, H3K27me3 has been implicated in Vκ gene repression (Levin-Klein et al., 2017). We have previously demonstrated that the

This is an open access article under the CC BY license (http://creativecommons.org/licenses/by/4.0/).

OPEN ACCESS
CellPress

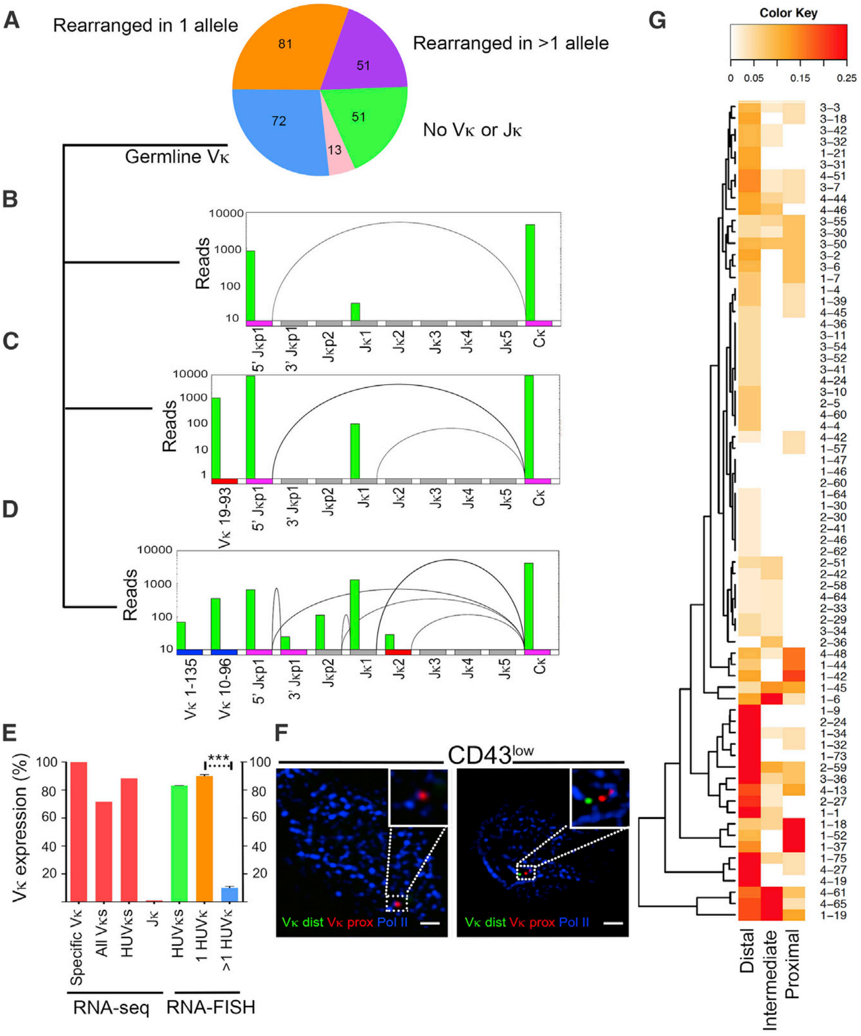

Figure 1. Monoallelic Activation of Vκ

(A) Single-cell RNA-seq *Igκ* expression profile of CD19^{+}B220^{+}CD43^{lo}IgM^{-} small pre-B cells. Of 268 cells captured, number of cells expressing germline VκJκ (blue), rearrangement in one *Igκ* allele (orange), rearrangement in more than one allele (purple), no Vκ or Jκ expression (green), or uncategorized (pink) are shown.

(B) Representative example of biallelic germline transcription of 5′Jκ1 promoter (magenta) without Vκ expression. mRNA splice products here and below are shown as arcs.

(C) Representative example of biallelic germline transcription of 5′Jκ1 promoter (magenta) with single Vκ expression from B6 allele (red).

(D) Representative example of biallelic germline transcription of 5′Jκ1 promoter (magenta) with multiple Vκ expression from CAST allele (blue).

(E) Percentage of cells with monoallelic expression as determined by either RNA-seq (left bars) or RNA-FISH (right bars). From left to right, percentages of cells expressing specific Vκ genes monoallelically (specific Vκ), cells expressing all Vκ genes from one allele (all Vκs), cells expressing highly used Vκs monoallelically (HUVκs), and monoallelic Jκ expression (Jκ) are shown. Based on RNA-FISH, percentage of cells monoallelically expressing one or more assayed HUVκs (green bar) is shown. Percentage of cells monoallelically expressing only one (1 HUVκ, orange) or >1 HUVκ (blue) is shown. Statistical significance was calculated on 100 cells per condition combined from two independent experiments by unpaired Student's t test (***$p < 0.001$).

(F) Representative images of RNA-FISH for nascent transcripts from all nine highly used Vκ genes combined with staining for e-Pol II (blue). Distal Vκs are in green and proximal in red. The scale bars represent 1 μm.

(G) Heatmap of Vκs expressed from distal (Vκ 2-137 to 13-84), intermediate (Vκ 4-81 to 6-32), and proximal (Vκ 8-30 to 3-1) loops in each single cell (cell number given on right). Color reflects fraction of Vκ genes in indicated domains expressed in individual cells (scale 0%–25%).

Vκ, but not Jκ, cluster genes are repressed in pro-B cells by cyclin D3 bound to the nuclear matrix (NM) (Powers et al., 2012). Repression is independent of CDK4/6-mediated proliferation and cannot be complemented by cyclin D2, which does not bind the nuclear matrix. However, how cyclin D3 mediates Vκ repression is not known.

Herein, we demonstrate that, in pro-B cells, the Vκ alleles are not repressed by H3K27me3. Rather, they are repressed by cyclin D3, which prevents productive association of Vκ gene TADs with serine 2 phosphorylated elongating RNA polymerase II (RNAP) on NM strands (transcription factories; Iborra et al., 1996; Osborne et al., 2004) surrounding the Vκ genes. Cell cycle exit then opens monoallelic repertoire of Vκ genes that are available for recombination. These and other findings reveal a mechanism by which large and stochastic monoallelic repertories of Vκ genes are opened prior to recombination to Jκ.

RESULTS

Monoallelic Vκ Transcription by Single-Cell RNA-Seq

To examine whether Vκ transcription prior to *Igκ* recombination was biallelic or monoallelic, we isolated B220^{+}CD19^{+} CD43^{low}IgM^{-} bone marrow (BM) small pre-B cells from a divergent F1 cross (C57BL/6 × CAST/EiJ) and subjected them to single-cell RNA sequencing. Preliminary bulk RNA-seq on this cell population suggested that it expressed Vκ GLT but had not undergone extensive *Igκ* rearrangement (data not shown). We then used CAST/EiJ- or C57BL/6-specific SNPs to assign expressed Vκ genes to the CAST or B6 genome, respectively.

From two experiments, we obtained 268 single-cell libraries (Figure 1A), with an average of 5.2×10^{6} 75-bp paired-end reads/cell and 83% concordant alignment rate. Of these, 51 cells did not express Vκ or Jκ genes, 81 cells had undergone recombination at single *Igκ* allele, and 51 had undergone recombination

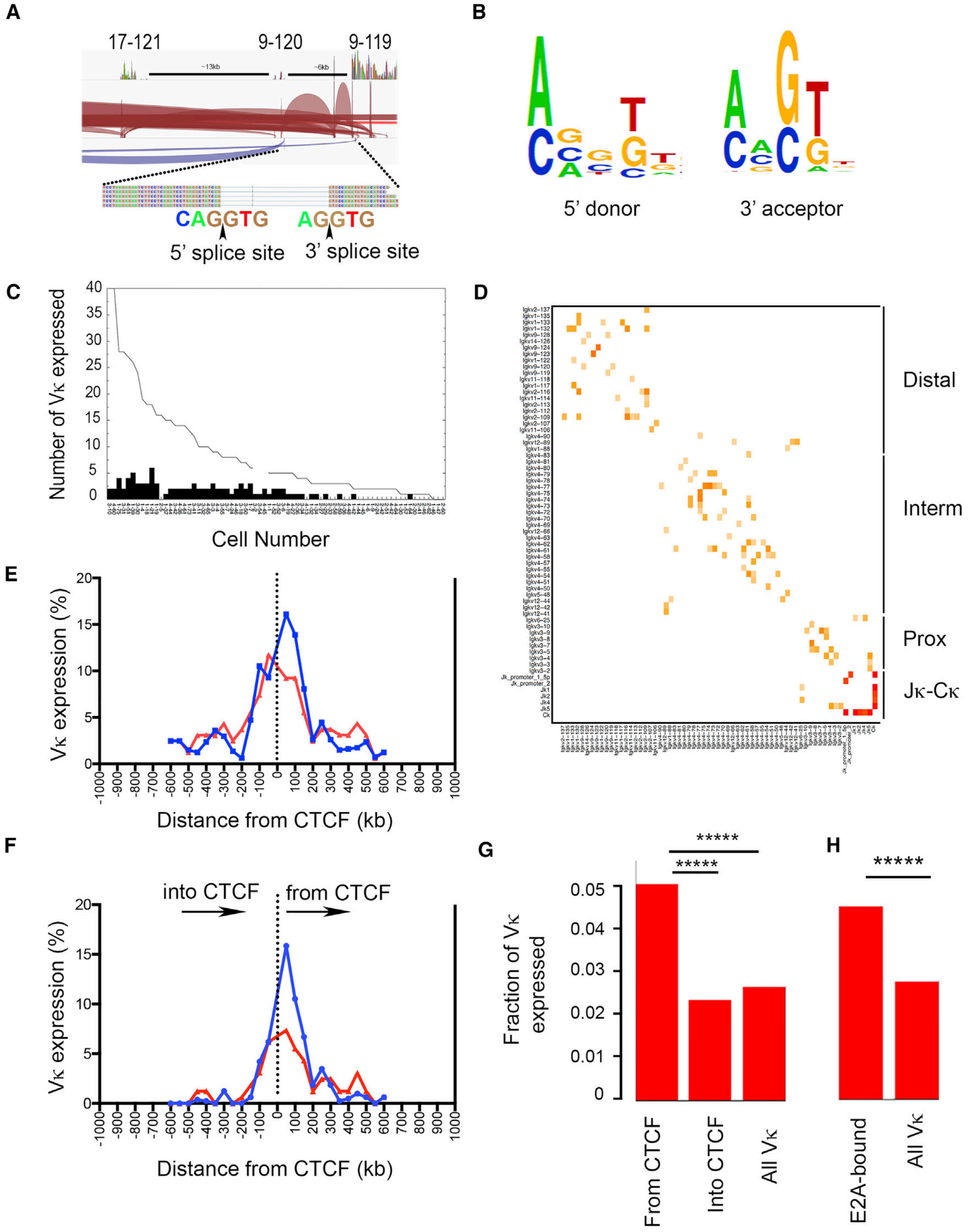

Figure 2. Monoallelic Expression of Vκ Chromatin Loops

(A) Representative example of Vκ-Vκ splicing from cell no. 1-75 (see Figure S2). Individual sense transcripts are shown in red and antisense transcripts shown in purple. Arcs represent indicated spliced transcripts, including splicing between Vκ 17-121, 9-120, and 9-119. 5′ and 3′ splice junctions for Vκ 9-120 and Vκ 9-119 are shown. Black arrow represents site where transcript is spliced. Genomic distances between Vκ genes are shown.

(B) Consensus splice junction motifs obtained from 5′ donor and 3′ acceptor sites when all splice junctions were analyzed.

(C) Total number of expressed Vκs interpolated from splicing from 72 cells poised for recombination. Black bars represent number of highly used Vκs per cell.

(D) Heatmap of spliced Vκ and Jκs across *Igκ* locus for all 72 poised single cells. Color reflects fraction of genes that underwent splicing (key in Figure 1G). Distal, intermediate, and proximal topologically associated domains (TADs) of *Igκ* are shown on the right.

(legend continued on next page)

at both *Igκ* alleles and/or *Igλ*. There were 13 cells that could not be categorized. The remaining 72 cells expressed Vκ and/or Jκ GLT (Figures 1A–1D). These latter 72 cells were poised for *Igκ* recombination, as evident by biallelic germline Jκ expression originating from the distal (Jκp1) and proximal promoters (Jκp2) and absence of recombination products (Figures 1B–1D). In this cell population, there also was no evidence of Vκ-Vκ recombination (data not shown).

Allelic expression was examined by assigning expressed Vκ genes in the 72 poised cells to either the B6 or CAST genome based on SNPs present in individual Vκ genes. There was good representation of Vκ genes expressed from both B6 and CAST alleles (Figure S1). Analysis of the 93 functional Vκ genes in each individual cell revealed that, for each Vκ gene, transcription only occurred at one allele (100% monoallelic expression; Figure 1E). Analyzing Vκ expression locus-wide revealed that 72% of cells expressed Vκs from only one allele (Figure 1E). This was in contrast to the ~99% cells (71 of 72) that expressed Jκ biallelically (Amin et al., 2009). When we focused only on those cells expressing the nine most highly used Vκs (Vκ1-135, Vκ 17-127, Vκ 9-120, Vκ 1-117, Vκ10-96, Vκ10-95, Vκ 6-23, Vκ 6-17, and Vκ 6-15; Aoki-Ota et al., 2012), eighty-eight percent of cells had monoallelic expression for these nine Vκ genes.

To confirm that highly used Vκ genes were primarily monoallelically expressed, we performed RNA-fluorescence *in situ* hybridization (FISH) on CD43^{low} small pre-B cells probing for all nine highly used Vκ GLT transcripts in the same cell, using a RNA probe cocktail (Table S1) specific for distal highly used Vκ (Alexa-488-labeled) and proximal highly used Vκ genes (Alexa-Fluor-647-labeled). In this assay, 83% of cells expressed highly used Vκ genes monoallelically (Figure 1F). Of those cells with monoallelic highly used Vκ expression, 90% expressed only one Vκ gene (Figures 1E and 1F). These data suggest that monoallelic Vκ transcription is a locus-wide phenomenon that provides a molecular basis for monoallelic *Igκ* recombination.

Long-Range Transcription over Multiple Vκ Genes

In the 72 cells poised for *Igκ* recombination, we found that multiple Vκ genes were expressed in 92% of cells with up to 16 per cell. Distal Vκ genes were expressed in almost all cells (Figure 1G), with 90% of cells expressing more than one. Forty-six percent of cells expressed both distal and proximal Vκ genes (Figure 1G). Forty-five of 72 poised cells (~60%) expressed highly used Vκs (Aoki-Ota et al., 2012), suggesting that these gene segments are commonly accessible (Figure S1).

Interestingly, in those cells expressing intermediate and/or proximal Vκ genes, transcription of the *Igλ* locus was common (Figure S1). These cells had not undergone *Igκ* recombination, suggesting that the opening of *Igλ* is not a consequence of *Igκ* recombination. Rather, our data suggest overlap in the mechanisms regulating accessibility at both loci.

Remarkably, Vκ to Vκ mRNA splicing was common. Shown in Figure 2A is an example demonstrating splicing between three Vκ genes (17-121, 9-120, and 9-119) in a single cell. In this example, splicing occurred between adjacent and non-adjacent Vκ genes. Vκ-Vκ splicing also occurred in the antisense direction. A similar pattern was observed in all cells, with splicing occurring both between adjacent Vκ genes and those separated by up to 32 intervening Vκ genes (data not shown). *De novo* analysis of all splice junctions revealed canonical 5′ donor and 3′ acceptor consensus sequences consistent with conventional mRNA splicing (Figure 2B).

Interpolation of intervening Vκs between spliced Vκs indicated that up to 40 Vκ genes were transcribed in a single cell and that 70% of cells transcribed at least one highly used Vκ gene (Figure 2C). Interpolation also indicated that transcription occurred over very long distances. The average inferred transcript length was 425 kb, with a maximum length of 1.1 mb. However, there were clear transcription boundaries. Examination of the distribution of Vκ-Vκ splicing revealed that it was strictly limited by boundaries predicted by CTCF sites to approximate the distal, intermediate, and proximal TADs (Lin et al., 2012; Figures 2D and S2). Germline Jκ-Cκ splicing was common (Figures 2D and S2). These data suggest that expression of multiple Vκ genes occurs by transcriptional readthrough and is common within TADs.

Vκ Transcription Initiated at CTCF Sites and E2A-Bound Promoters

Transcription has been shown to occur at or near CTCF sites, whereby CTCF can directly associate with RNAP and anchor transcription (Chernukhin et al., 2007). We therefore examined whether Vκ transcription occurred close to CTCF-bound sites in single cells. Comparing published pro-B Hi-C data and CTCF ChIP-seq data, there are eight CTCF-bound sites (S1–S8) within the *Igκ* locus with high likelihood for loop formation (Figures S1 and S2; Choi and Feeney, 2014; Lin et al., 2012). The distal Vκ TAD is bound by S1 and S5, the intermediate TAD by S5 and S6, and the proximal TAD by S6 and S8 (Figures S1 and S2). We first plotted Vκ frequencies as a function of genomic distance from individual CTCF sites and compared this to predicted frequencies considering random distribution of Vκ expression (Figure 2E). For this analysis, Vκ genes 5′ to a CTCF-bound site were plotted as negative distances and those 3′ as positive distances. As can be seen, there was an overall bias to transcribe Vκ genes near CTCF-bound sites (Figure 2E).

(E and F) Frequency of Vκ expression as a function of distance from CTCF sites. In (E), Vκ expression relative to CTCF sites with Vκs 5′ to CTCF on left and those 3′ plotted on right are shown. Plot regardless of Vκ gene orientation is shown. In (F), plot of expression of Vκ genes either oriented toward or away from CTCF sites as a function of distance is shown. For both plots, observed expression frequencies are shown in blue and expected frequencies if expression was randomly distributed shown in red.

(G) The number of Vκs expressed that are oriented away from or into CTCF sites over total Vκs oriented away from or into CTCF sites is shown as “from CTCF” and “into CTCF” fraction (Figure 1F), respectively. All expressed Vκs over total Vκs are shown as “all Vκ” fraction. Fisher’s exact test was performed between group pairs over all single cells. (From CTCF to into CTCF: *****$p = 4.37 \times 10^{-13}$; from CTCF to all Vκ: *****$p = 7.23 \times 10^{-9}$).

(H) The number of Vκs expressed that have E2A at promoters in pro-B cells over total E2A-promoter loaded Vκs is shown as “E2A-associated” fraction, and all expressed Vκs over total Vκs are shown as all Vκ fraction. Fisher’s exact test was performed between the two groups over all single cells (*****$p = 4.9 \times 10^{-39}$).

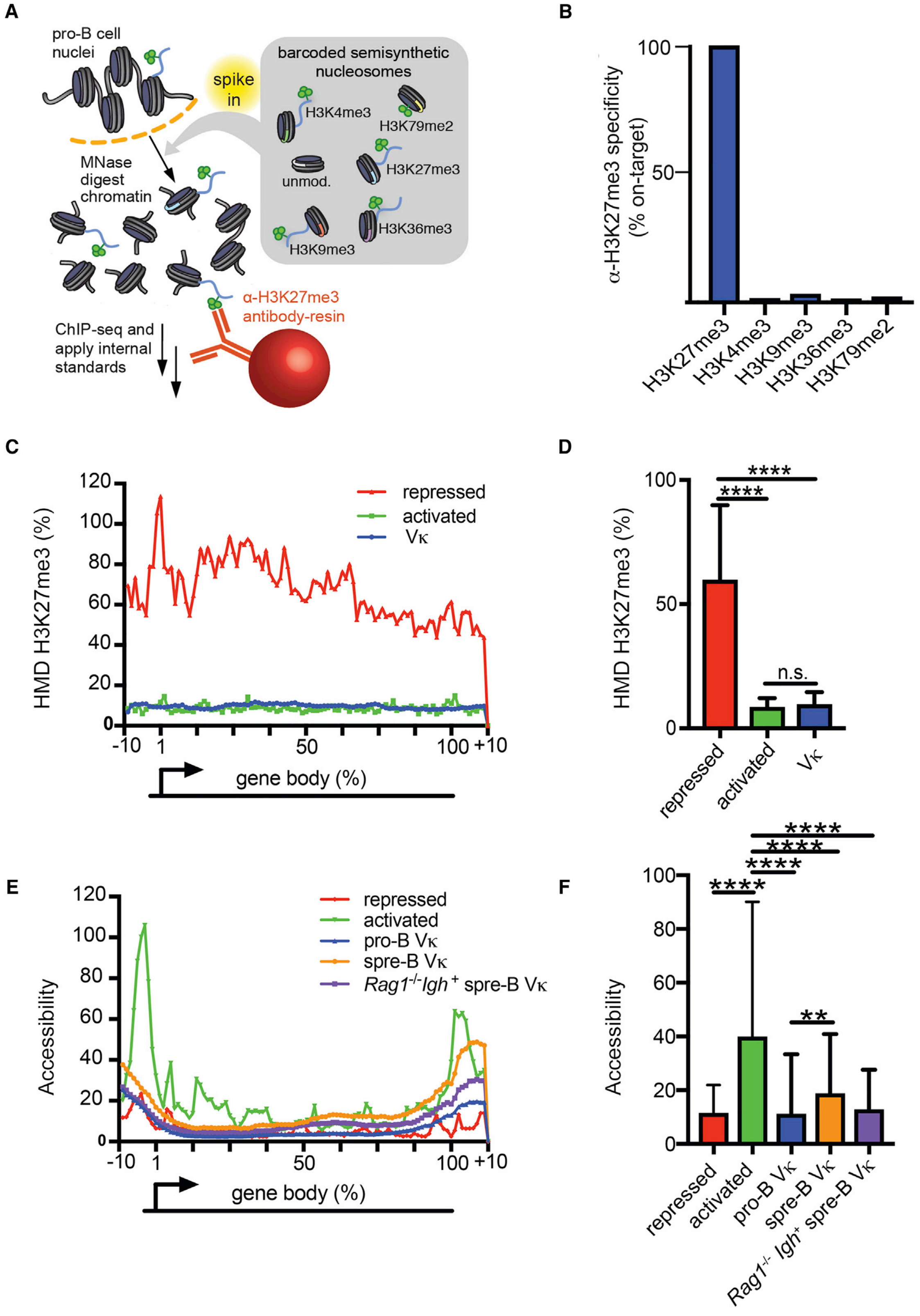

(legend on next page)

Throughout the *Igκ* locus, Vκ genes occur in both orientations (Figure S1). We next examined Vκ gene transcription bias due to their orientation from CTCF sites. We plotted expressed Vκ genes as a function of orientation and distance from CTCF sites. As seen in Figure 2F, there was a strong bias to transcribe Vκ genes oriented away from CTCF-bound sites compared to a random distribution and this bias persisted out to 150 kb.

Not all Vκ genes are functional (Martinez-Jean et al., 2001; Figure S1). We next assessed functional Vκ gene transcription bias. We plotted expressed Vκ genes as a fraction of total known functional Vκ genes in each indicated orientation relative to CTCF-bound sites. As can be seen, there was a significant bias for transcribing functional Vκ genes oriented away from CTCF sites compared to transcribing those oriented toward CTCF sites or when no orientation bias was considered (Figure 2G).

The transcription factor E2A is important for *Ig* gene transcription (Romanow et al., 2000). We therefore examined the relative frequency of Vκ gene expression for those with promoters prebound by E2A in pro-B cells (E2A ChIP-seq; Lin et al., 2010) compared to the frequency of expression for all Vκ genes. As demonstrated in Figure 2H, E2A-associated Vκs were expressed at a significantly higher frequency compared to overall Vκs. These data suggest Vκ transcription can be initiated at or near CTCF-bound sites and at the promoters of Vκ genes preloaded with transcription factors.

Vκ Genes Are Not Repressed by H3K27me3 in Pro-B Cells

We next asked how Vκ transcription was regulated. Transcription is often repressed epigenetically, with histone methylation (H3K27me3) being a common mechanism. However, the data regarding the role of H3K27me3 in Vκ gene repression have been conflicting (Levin-Klein et al., 2017; Mandal et al., 2011; Xu and Feeney, 2009). In part, this confusion reflects the limitation of conventional ChIP-seq, which lacks internal standards and is therefore qualitative. Therefore, to determine whether the Vκ genes are, or are not, marked with H3K27me3, we performed an internally calibrated ChIP (ICeChIP) (Grzybowski et al., 2015). The ICeChIP both performs an *in situ* antibody specificity test and displays data on a biologically meaningful scale, the histone modification density (HMD), representing the fraction of all nucleosomes bearing a specific histone mark at a given genomic locus. These attributes allowed us to quantify the amount of H3K27me3 at the Vκ genes on an absolute scale.

Therefore, chromatin was isolated from *Rag2*$^{-/-}$ pro-B cell nuclei and then "spiked" with semisynthetic nucleosome standards, each containing a single histone post-translational modification (H3K27me3, H3K4me3, H3K9me3, H3K36me3, or H3K79me2) and a unique bar-coded DNA strand (Figure 3A). Samples were then immunoprecipitated with antibodies specific for H3K27me3 and subjected to next generation sequencing.

Analysis of the ICeChIP data revealed that the immunoprecipitating antibody we used specifically bound H3K27me3 and did not appreciably react with other histones methyl modifications (Figure 3B). To establish a positive control, we averaged the H3K27me3 HMD at representative genes, which were marked with H3K27me3 and not expressed in pro-B cells (Mandal et al., 2011; Table S2). As a negative control, we averaged the H3K27me3 HMD at representative genes actively transcribed in pro-B cells. Interestingly, H3K27me3 HMD at inactive Vκ genes was much lower than that observed in other repressed genes and was comparable to levels observed in activated genes (Figures 3C and 3D). These data suggest that, prior to recombination, the Vκ genes are not repressed by a H3K27me3-dependent mechanism.

We then examined Vκ accessibility in pro-B and small pre-B wild-type (WT) cells using the assay for transposase-accessible chromatin (ATAC)-seq (Figures 3E and 3F). In the activated and repressed control genes used above, there were corresponding substantial differences in accessibility density, especially immediately upstream of the gene body. This likely reflects differences in promoter accessibility. In contrast, there were only small differences in Vκ gene accessibility between pro-B cells, where the Vκ genes are repressed, and small pre-B cells, where Vκ genes are transcribed and undergo *Igκ* recombination. Accessibility of the Vκ genes was similar in *Rag1*$^{-/-}$*Igh*$^{+}$ small pre-B cells, which are primed for *Igκ* recombination. Therefore, Vκ gene transcription and susceptibility to recombination are not associated with intrinsic changes in Vκ gene accessibility. These findings, in conjunction with our observation that the Vκ genes are not repressed by H3K27me3, suggest that nonconventional mechanism(s) regulate Vκ gene transcription.

The Vκ Repressor, Cyclin D3, Associates with NM-RNAP

Our earlier work has revealed that Vκ transcription in pro-B cells is repressed by cyclin D3 bound to the NM (Powers et al., 2012). RNAP is also assembled on the NM in supra-molecular complexes termed "transcription factories." Translocation of genes to such fixed sites has been suggested as a mechanism for transcriptional activation (Iborra et al., 1996; Osborne et al., 2004). However, how this mechanism of transcription is regulated is unknown. To examine whether there might be a functional

Figure 3. In Pro-B Cells, the Vκ Genes Lack Appreciable H3K27me3

(A) Schematic representation of ICeChIP-seq.

(B) ICeChIP-seq-based specificity measurement of αH3K27me3 (CST C36B11) antibody. Specificity is expressed as a fraction of normalized H3K27me3 nucleosome capture.

(C) Meta-analysis of H3K27me3 ICeChIP displaying the average HMD over the length of each gene body for the following gene sets: Vκ regions; activated genes; and repressed genes in pro-B cells (Table S2).

(D) H3K27me3 HMD in pro-B cells comparing Vκ gene segments to activated genes and repressed genes as in (C).

(E) Meta-analysis of accessibility (ATAC-seq) displaying the average HMD over the length of each gene body for the following gene sets: Vκ regions in pro-B cells (pro-B); Vκ regions in small pre-B cells (spre-B); Vκ regions in *Rag1*$^{-/-}$*Igh*$^{+}$ small pre-B cells; activated genes in pro-B cells; and repressed genes in pro-B cells.

(F) Accessibility calculated from −10% to +10% relative to the transcription start site (TSS) for each gene set displayed.

(D–F) Statistical significance was determined by ANOVA ($p < 0.0001$ and $p < 0.0001$, respectively) in combination with Tukey's multiple comparison test. Error bars represent the average ± SD. **$p \leq 0.01$; ****$p \leq 0.0001$.

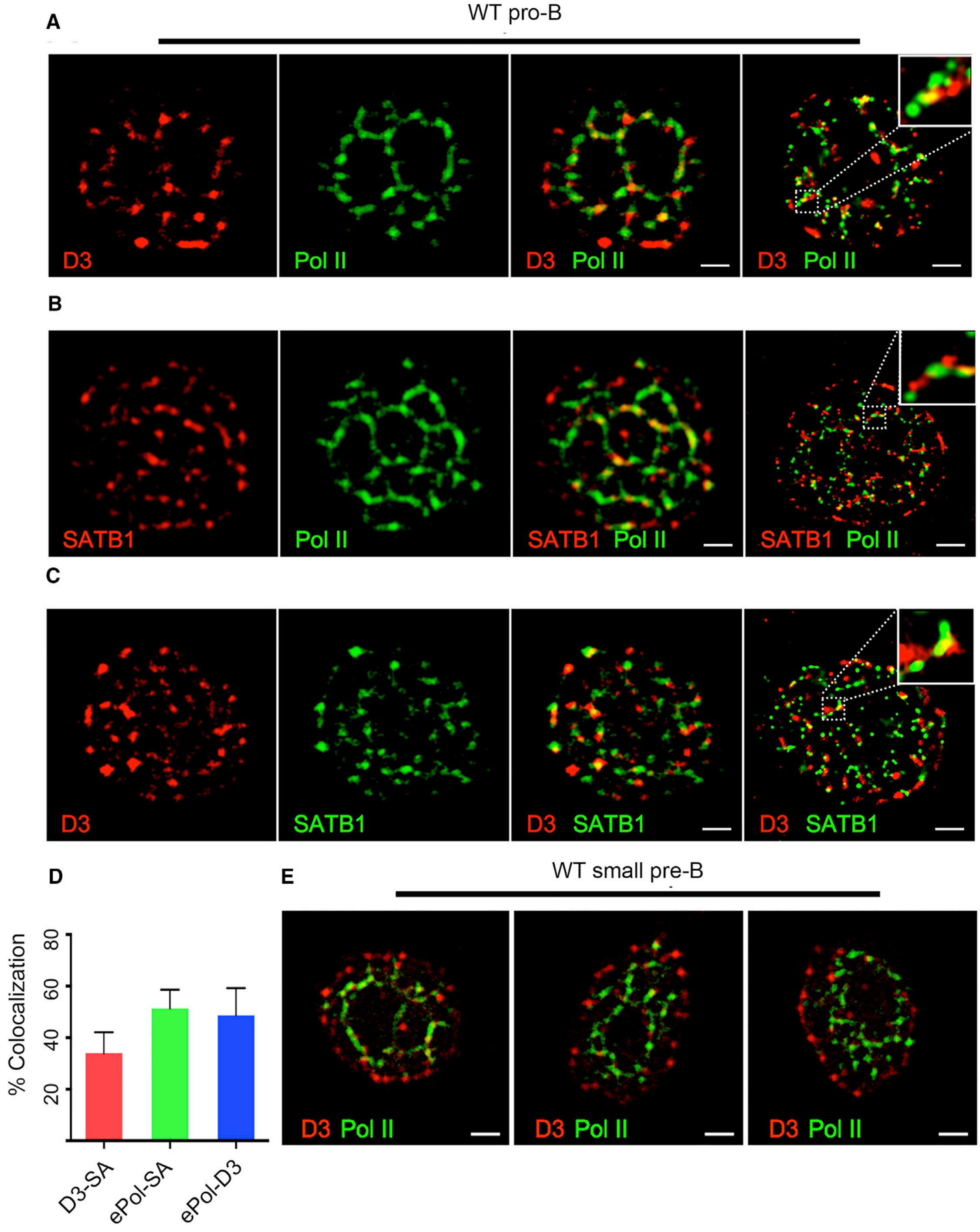

Figure 4. Cyclin D3 Is Assembled with RNAP on the Nuclear Matrix

(A) Representative confocal images (from 40 cells; n = 2 experiments) of WT pro-B cells washed 10× (CSK+0.5%Triton) to remove soluble nuclear proteins and then fixed and stained with antibodies specific for cyclin D3 and e-Pol II (RNAP). Super-resolution image of similarly stained WT pro-B cells (right panel) is shown. The scale bars represent 1 μm.

(B) Representative confocal images (40 cells; n = 2 experiments) of WT pro-B cells washed and fixed as above and then stained with antibodies specific for SATB1 and e-Pol II. Super-resolution image of similarly stained WT pro-B cells (right panel) is shown. The scale bars represent 1 μm.

(C) Representative confocal images (40 cells; n = 2 experiments) of WT pro-B cells washed and fixed as above and then stained with antibodies specific for SATB1 and cyclin D3. Super-resolution image of similarly stained WT pro-B cells (right panel) is shown. The scale bars represent 1 μm.

(legend continued on next page)

relationship between NM-bound cyclin D3 and RNAP, we used confocal microscopy to visualize their spatial relationships on the NM. WT pro-B cells were permeabilized and then washed extensively under mild conditions (cytoskeletal stabilizing buffer [CSK]+0.5% Triton) to remove soluble nuclear proteins (Figure S3A; Sawasdichai et al., 2010). Cells were then fixed, stained with antibodies specific for cyclin D3 or RNAP, and visualized by confocal microscopy. These studies revealed that, in pro-B cells, NM-bound cyclin D3 either co-localized (55% Mander's coefficient) or was closely apposed with RNAP on apparent strands that formed a "cage-like" pattern (Figures 4A and 4D). Co-staining with antibodies specific for the NM-bound molecule, special AT-rich sequence-binding protein 1 (SATB1), revealed its co-localization with both cyclin D3 and RNAP (35% and 50%, respectively; Figures 4B–4D and S3B) in a similar cage-like pattern. To better visualize these RNAP/cyclin D3 complexes, we used super-resolution microscopy (Leica Ground State Depletion), which provides 20-nm resolution in the X-Y plane. This higher resolution revealed that cyclin D3, RNAP, and SATB1 were intertwined, forming nanometer-scale fibrils throughout the nucleus (Figures 4A–4C and S3B, far right panels).

At the small pre-B cell stage, cyclin D3 transcription is repressed, enabling cells to exit cell cycle and initiate *Igκ* recombination (Cooper et al., 2006). Interestingly, in WT small pre-B cells, cyclin D3 had largely translocated to the periphery of the nucleus away from RNAP (Figure 4E). These data reveal that the spatial relationship between cyclin D3 and RNAP is developmentally regulated.

Cyclin D3 Regulates Monoallelic Vκ Association with NM-RNAP

We next examined the spatial relationships between cyclin D3, RNAP, and the Vκ gene cluster. WT or *Ccnd3*$^{-/-}$ pro-B cells were washed as above, fixed, and then subjected to FISH with a 488-labeled bacterial artificial chromosome (BAC) probe, RP-23 182E6, that binds a 0.2-mb region spanning ~10 distal Vκ gene segments (Vκ 2-113 to 1-122), followed by staining with antibodies specific for cyclin D3 and RNAP (immunoFISH). In WT pro-B cells, both Vκ alleles were surrounded by NM RNAP-cyclin D3 complexes (Figure 5A). However, Vκ genes and RNAP rarely co-localized (<5%; Figures 5A and 5D). In contrast, in *Ccnd3*$^{-/-}$ pro-B cells and WT small pre-B cells, Vκ genes frequently co-localized with NM RNAP (35%–40%; Figures 5B–5D). Co-localization of Vκ genes to RNAP was specific to *Igκ*, as *TCRβ* (Vβ) was rarely found to associate with RNAP in WT small pre-B cells (Figure S4A).

Almost invariably (>95%), only one Vκ allele co-localized with RNAP in *Ccnd3*$^{-/-}$ pro-B and WT small pre-B cells (Figures 5B–5D), consistent with single-cell RNA sequencing (scRNA-seq) findings. Furthermore, in both *Ccnd3*$^{-/-}$ pro-B cells and WT small pre-B cells, the early replicating allele, previously shown to undergo recombination (Farago et al., 2012), associated with RNAP (Vκ doublet; Figures S4B and S4C). This latter observation suggests that Vκ transcription initiates at the *Igκ* allele fated to recombine first.

We next examined the association of Jκ cluster with RNAP and its regulation by cyclin D3 using a BAC probe spanning the entire Jκ cluster and Cκ (RP24-387E13). In contrast to Vκ, we found that Jκ did not co-localize with NM-RNAP in WT pro-B or *Ccnd3*$^{-/-}$ pro-B cells (Figures 5E, 5F, and 5H). This is consistent with previous findings that Jκ GLT is not regulated by cyclin D3 but by the reciprocal action of STAT5 and E2A (Mandal et al., 2011; Powers et al., 2012). In WT small pre-B cells, both Jκ alleles co-localized (40%) with RNAP (Figures 5G and 5H; Amin et al., 2009). These results suggest that monoallelic Vκ transcription is achieved by monoallelic co-localization of Vκ to NM-RNAP. Furthermore, cyclin D3 represses co-localization of the Vκ gene cluster, but not Jκ cluster, with NM-RNAP.

Vκ-Jκ contraction is necessary for recombination. To examine whether the Vκ allele co-localizing with RNAP marks the allele that undergoes Vκ-Jκ contraction, we performed 2-color Vκ and Jκ DNA-FISH in combination with RNAP immunofluorescence (IF) on WT small pre-B cells (Figures 5I, 5J, and S4F). As demonstrated, the *Igκ* allele in which Vκ genes were co-localized with RNAP (Vκ1-Jκ1) was more contracted (mean < 200 nm) than the allele in which Vκ genes were not co-localized with RNAP (mean > 200 nm). In contrast, in WT pro-B, there was no Vκ gene translocation to RNAP and both alleles were not contracted (Figures S4D and S4G). Monoallelic Vκ gene translocation to RNAP and contraction was also observed in *Ccnd3*$^{-/-}$ pro-B cells (Figures S4E and S4H), where Jκ is still repressed (Figure S4I; Powers et al., 2012). These findings suggest that the Vκ gene cluster allele, which translocates to RNAP, marks the contracting and therefore recombining allele. Furthermore, contraction can happen independently of both Jκ GLT and Vκ-Jκ recombination.

To equate Vκ and RNAP co-localization with transcription, we combined DNA-FISH with RNA-FISH using a RNA probe for a single germline Vκ (highly used Vκ 1-117) transcript encompassed within the RP-23 182E6 BAC probe used for DNA FISH (Figure 6A; Table S3). In both *Ccnd3*$^{-/-}$ pro-B and WT small pre-B cells, we detected a Vκ 1-117 GLT either co-localized or in close proximity to a single Vκ gene/RNAP complex (Figures 6B, 6C, S5A, and S5B). Consistent with scRNA-seq data, in those cells expressing Vκ 1-117 germline transcripts, transcription was monoallelic in ~95%–98% of WT small pre-B cells and *Ccnd3*$^{-/-}$ pro-B cells (Figure 6E).

We then examined Jκ GLT in WT small pre-B cells using a RNA probe complementary to a region 5′ of Jκ1 and 3′ of the distal promoter (Figure 6A; Table S3). Biallelic GLT Jκ was noted in nearly 40% of cells that had transcripts (Figures 6D, 6E, and S5C). These data confirm that monoallelic Vκ translocation to RNAP leads to monoallelic transcription, whereas biallelic Jκ translocation to RNAP leads to biallelic transcription.

(D) Percent co-localization of elongating RNAP-D3 (ePol-D3), D3-SATB1 (D3-SA), and RNAP-SATB1 (ePol-SA) stains calculated by using Manders on 30 2D confocal images per samples (n = 2 experiments).

(E) Representative confocal images (40 cells; n = 2 experiments) of WT small pre-B cells washed as above and stained with antibodies specific for cyclin D3 and RNAP. The scale bars represent 1 μm.

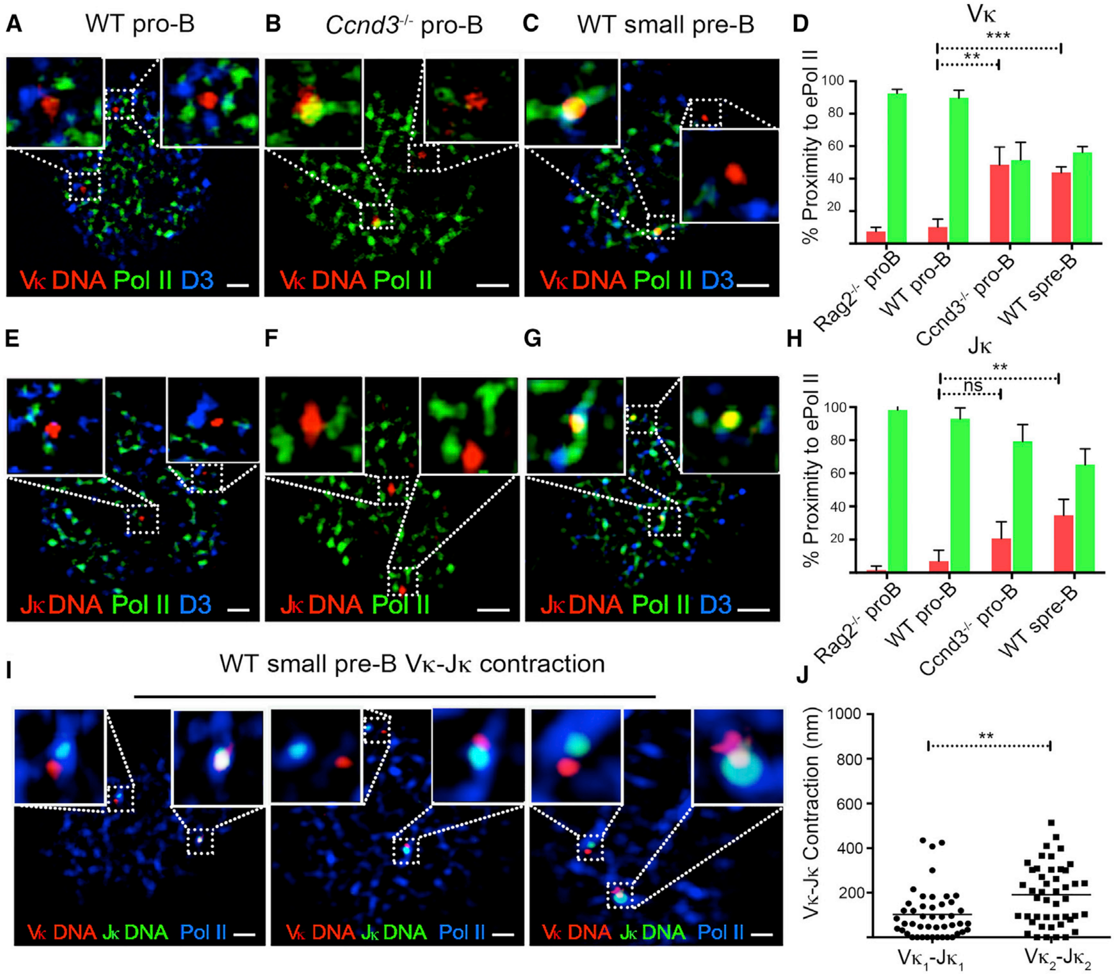

Figure 5. Cyclin D3 Regulates Vκ, but Not Jκ, Association with RNAP

(A) Representative confocal image (50 cells; n = 3 experiments) of WT pro-B cells washed extensively to remove soluble nuclear proteins and then hybridized with Vκ DNA probe (RP23-182E6) spanning 10 distal Vκ genes (Vκ2-113 to 1-122) and stained with antibodies specific for cyclin D3 and e-Pol II (RNAP). The scale bar represents 1 μm.

(B) Representative confocal image (50 cells; n = 3 experiments) of *Ccnd3*$^{-/-}$ pro-B cells washed, hybridized with Vκ DNA probe (RP23-182E6), and stained with antibodies specific for e-Pol II. The scale bar represents 1 μm.

(C) Representative confocal image (50 cells; n = 3 experiments) of WT small pre-B cells washed, hybridized with Vκ DNA probe (RP23-182E6), and stained with antibodies specific for cyclin D3 and e-Pol II. The scale bar represents 1 μm.

(D) Percent Jκ co-localized (red) and not co-localized (green) to e-Pol II scored on confocal images of 50 nuclei per sample (n = 3 experiments), and plotted for each sample. Co-localization scored when at least one Vκ allele engaged e-Pol II. Statistical significance was calculated by unpaired Student's t test (**$p < 0.01$ and ***$p < 0.001$).

(E) Representative confocal image (50 cells; n = 3 experiments) of WT pro-B cells washed, hybridized with Jκ DNA probe (RP24-387E13) spanning Jκ- Cκ, and stained with antibodies specific for cyclin D3 and e-Pol II. The scale bar represents 1 μm.

(F) Representative confocal images (50 cells; n = 3 experiments) of *Ccnd3*$^{-/-}$ pro-B cells washed, hybridized with Jκ DNA probe (RP24-387E13), and stained with antibodies specific for e-Pol II. The scale bar represents 1 μm.

(G) Representative confocal images (50 cells; n = 3 experiments) of WT small pre-B cells washed, hybridized with Jκ DNA probe (RP24-387E13) spanning JκCκ, and stained with antibodies specific for cyclin D3 and e-Pol II. The scale bar represents 1 μm.

(H) Percent Jκ co-localized (red) and not co-localized (green) to e-Pol II scored on confocal images of 50 nuclei per sample (n = 3 experiments) and plotted for each sample. Co-localization was scored when at least one Jκ allele engaged e-Pol II. Statistical significance was calculated by unpaired Student's t test (**$p < 0.01$).

(I) Representative confocal images (40 cells; n = 2 experiments) of WT small pre-B cells, washed to remove soluble nuclear proteins, hybridized to Vκ DNA probe RP23-182E6 (red) and Jκ DNA probe RP24-382E13 (green), and stained with antibodies specific for e-Pol II (blue). The scale bars represent 1 μm.

(J) Minimum distances between Vκ and Jκ in WT small pre-B cells plotted for the allele in each cell closer to RNAP (Vκ1-Jκ1) and the allele farther removed from RNAP (Vκ2-Jκ2). Distances between Vκ and Jκ were calculated using Euclidean Distance Transformation on Imaris. Analysis was performed on cells imaged as in (I). Statistical significance was calculated (n = 37) by paired Student's t test (**$p < 0.01$).

OPEN
ACCESS
CellPress

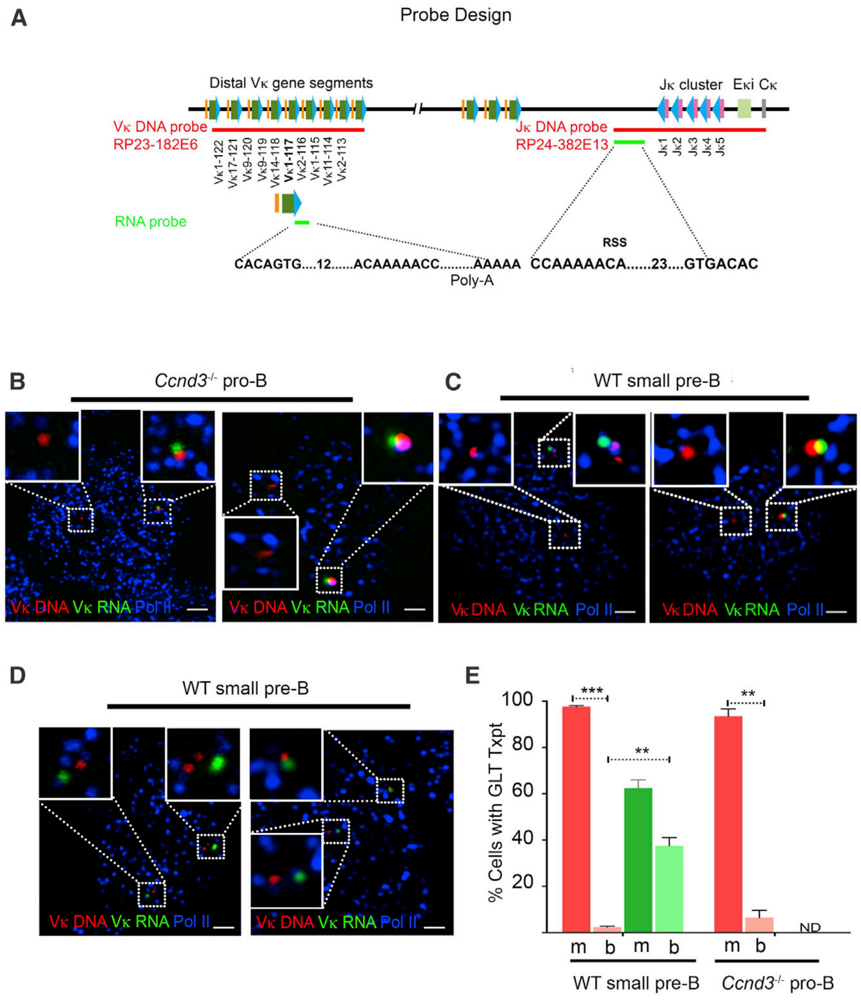

Figure 6. Monoallelic Vκ Transcription Repressed by Cyclin D3

(A) Schematic of DNA and RNA probes used in the study. Vκ DNA probe targets distal Vκ gene segments (Vκ 113-122), whereas RNA probe only targets a single Vκ gene (1-117). The RNA probe binds to region 3′ of the RSS site and therefore targets unrearranged, germline transcripts. Jκ DNA probe targets Jκ-Cκ region, whereas the Jκ RNA probe targets germline sequences downstream of the distal promoter (5′ of Jκ1) and 3.5 kb upstream of Jκ1 (Table S3).

(B) Representative confocal images (40 cells; n = 2 experiments) of *Ccnd3*$^{-/-}$ pro-B cells, washed extensively to remove soluble nuclear proteins and then hybridized to Vκ DNA probe RP23-182E6, and stained with antibodies specific for e-Pol II followed by hybridization with RNA probe targeting 3′ of Vκ 1-117. Shown are two representative examples, arranged horizontally. The scale bars represent 1 μm.

(C) Representative confocal images (40 cells; n = 2 experiments) of WT small pre-B cells, washed, hybridized, and stained as in (B). Shown are two representative examples, arranged horizontally. The scale bars represent 1 μm.

(D) Representative confocal images (40 cells; n = 2 experiments) of WT small pre-B cells, washed, hybridized with Jκ DNA probe RP24-387E13, and stained with antibodies specific for e-Pol II followed by hybridization with RNA probe targeting 5′ to Jκ. Shown are two representative examples, arranged horizontally. The scale bars represent 1 μm.

(E) Percent WT small pre-B cells and *Ccnd3*$^{-/-}$ pro-B cells with Vκ (red) or Jκ (green) germline transcripts scored on 50–60 nuclei per sample (n = 2 experiments) and further scored for monoallelic (m) versus biallelic (b) transcription. Statistical significance was calculated by unpaired Student's t test (**p < 0.01 and ***p < 0.001).

Cyclin D3 Is a General Repressor of Monoallelic Genes

Deletion of cyclin D3 induced expression of approximately 200 genes in addition to Vκ genes in pro-B cells (Powers et al., 2012). These genes were hierarchically ranked by fold increase overexpression in WT pro-B cells, and those with equal or greater than a three-fold increase were plotted (Figure 7A). We next interrogated the Database of Autosomal Monoallelic Expression (dbMAE) (Savova et al., 2016; Figures 7A–7C). In this database, genes are considered to have random monoallelic expression (MAE) if they demonstrate monoallelic expression in cell clones from several different tissues. Of the 65 genes upregulated three-fold or higher in *Ccnd3*$^{-/-}$ pro-B cells, 62 could be assessed for MAE either directly or indirectly by epigenetic marks. Interestingly, 63% (39 of 62) of cyclin-D3-repressed genes have been directly demonstrated to be monoallelically expressed in at least five different F1 tissues, including B cells (dark blue bars, Figure 7A). Only 11% (7) genes were biallelically expressed in multiple tissues (green, Figure 7B).

We then compared the frequency of randomly monoallelic genes in *Ccnd3*$^{-/-}$ pro-B cells to that in WT pro-B cells (~10,000 genes) using the criteria above (Figure 7D, left) or a more stringent criteria, in which monoallelic expression must occur in at least ten tissues (Figure 7D, right). With either criterion, cyclin D3 preferentially repressed randomly monoallelic expressed genes in pro-B cells. As was observed for the Vκ genes, cyclin-D3-repressed genes were not appreciably marked with H3K27me3 (Figures 7E and 7F). These data suggest that non-epigenetic cyclin-D3-mediated repression is a general mechanism linking cell cycle exit to random monoallelic expression.

DISCUSSION

For Vκ-Jκ recombination, Vκ genes must be made accessible through mechanisms that both ensure developmental-stage-specific recombination yet allow diverse use of functional Vκ gene segments arrayed over approximately 3 mb. Herein, we demonstrate that Vκ-gene-containing TADs are stochastically captured by transcription factories that then track and transcribe over long distances to open multiple Vκ genes. In each cell, different TADs can be stochastically captured by different transcription factories and transcription initiated at one of multiple

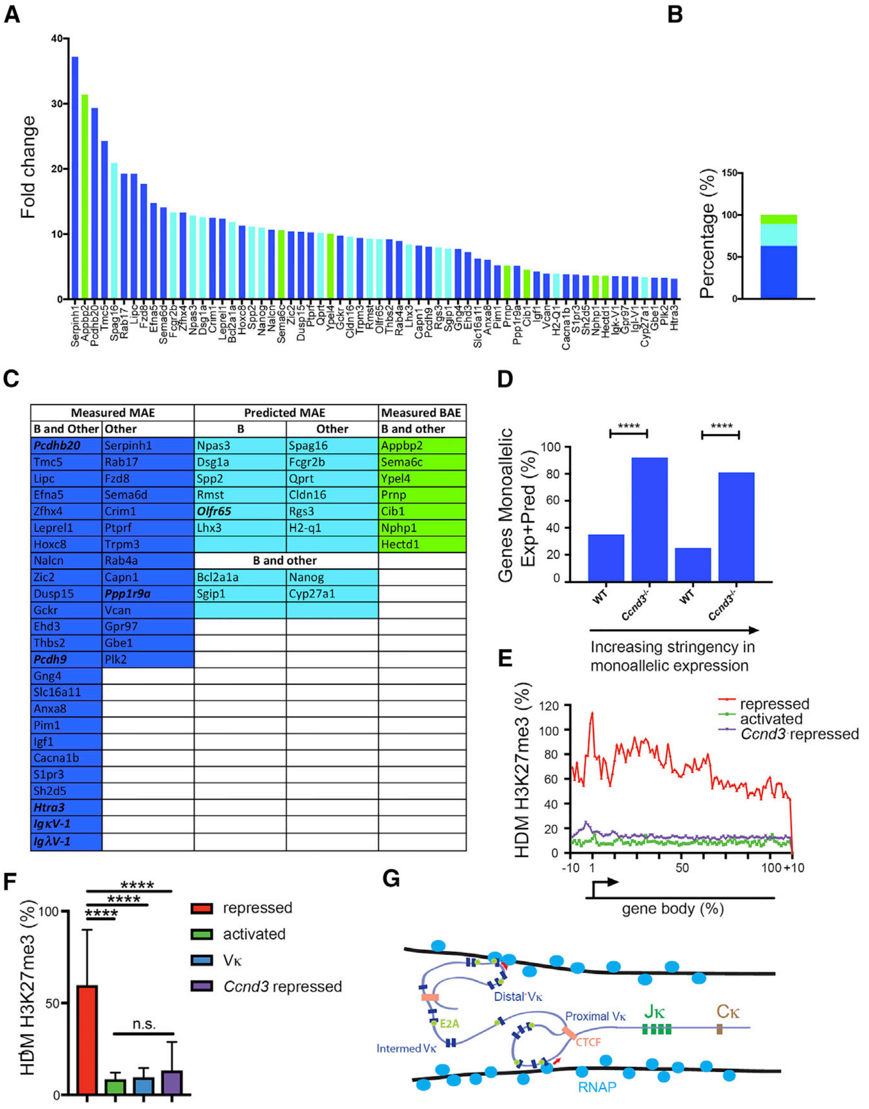

Figure 7. Cyclin D3 Represses Other Monoallelically Expressed Genes

(A) Upregulated genes in *Ccnd3*$^{-/-}$ pro-B cells on microarray hierarchically ranked by fold increase over WT pro-B cells (= >3-fold) plotted. Genes experimentally measured as monoallelic in B and other tissues are shown in blue, those predicted monoallelic in B cells and other tissues are shown in light blue, and those that are measured biallelic in B cells and other tissues are shown in green.

(B) Percentage of genes measured as in (A). Monoallelic (MAE) in B cells and other tissues (blue), predicted monoallelic (light blue), and biallelic (BAE) in B cells and other tissues (green) are shown.

(C) List of monoallelic and biallelic genes in *Ccnd3*$^{-/-}$ pro-B cells, where genes are color coded as in (A) and (B). Imprinted genes (Ppp1r9a and Htra3), olfactory receptor genes (Olfr65), Igκ variable (IgκV-1), Igλ (IgλV-1) variable, and protocadherin genes (Pcdhb20 and Pcdh9) are bold and italicized.

(D) Percentage of randomly monoallelic genes expressed in WT and *Ccnd3*$^{-/-}$ pro-B cells. Two criteria are used. Bars on left show fraction of expressed genes known to be monoallelically expressed in at least three of five B cell lines and in at least five other tissues. Bars on right show fraction of expressed genes known to be monoallelically expressed in at least ten other tissues. Statistical significance was measured by Fisher's exact t test (left, ****$p = 1.3 \times 10{-}07$; right, ****$p = 2.4 \times 10{-}09$).

(E and F) Metagene analysis of H3K27me3 ICeChIP displaying the average HMD over the length of each gene body for the following gene sets: *Ccnd3* repressed genes; activated genes; and repressed genes in pro-B cells (E). H3K27me3 HMD in pro-B cells comparing *Ccnd3* repressed genes to Vκ gene-segments, activated genes, and repressed genes (F) is shown. Statistical significance was determined by ANOVA $p < 0.0001$ in combination with Tukey's multiple comparison test. Error bars represent the average ± SD. ****$p \leq 0.0001$.

(G) Model of Vκ repertoire diversity. Our data indicate that Vκ genes are surrounded by transcription factories (NM-bound RNAP; cross-section shown). With loss of cyclin D3, Vκ-gene-containing TADs can stochastically engage one or more transcription factories with transcription either being initiated at E2A-bound promoters (red arrows) or CTCF sites.

E2A-bound promoters or CTCF sites (Figure 7G). In this way, unique stochastic repertoires of Vκ genes are transcribed in each cell. We propose that these transcribed Vκ genes define the repertoire available for recombination to Jκ (Yancopoulos and Alt, 1985). In any one cell, Vκ transcription is initiated primarily at one allele and transcription is repressed by cyclin D3. Therefore, our studies provide a mechanism for understanding several important features of *Igκ* recombination, including the generation of Vκ diversity, monoallelic choice, and the restriction of recombination to non-dividing cells.

Capture of genes by fixed RNAP factories has been observed for other genes, including c-*myc* (Osborne et al., 2007), and other clustered genes, including *Igh* (Park et al., 2014), protocadherin genes (Guo et al., 2012), and olfactory receptor genes (Clowney et al., 2012). Indeed, it has been proposed to be the most common mechanism of transcriptional activation (Papantonis and Cook, 2013). Stochastic chromatin loop capture provides a mechanism by which Vκ genes, arrayed over large genomic distances, could be coordinately and stochastically expressed in individual cells.

Transcription of Vκ genes tended to be initiated at Vκ promoters, especially those known to be pre-bound by E2A (Lin et al., 2010). However, we also observed a strong preference for transcribing Vκ genes that were proximate to, and oriented away from, CTCF-bound sites. This is consistent with chromatin loops being captured at or near CTCF-bound sites with only transcription away from these sites being productive. This is not unexpected, as it is known that RNAP is recruited to CTCF sites (Chernukhin et al., 2007). Furthermore, two anchor mechanisms for initiating transcription is consistent with the pattern of Vh

usage observed in the primary *Igh* repertoire (Bolland et al., 2016), suggesting a similar underlying mechanism.

Previous studies have relied on bulk cultured pre-B cells or pre-B cell clones from F1 (C57BL/6 × CAST/EiJ) mice, in which *Igκ* recombination is induced by withdrawing IL-7 or by shifting temperatures in a sensitive A-MuLV cell line (Farago et al., 2012; Levin-Klein et al., 2017). These experimental approaches have significant limitations. Most notably, cell cycle exit and Igκ recombination progresses asynchronously in these cell populations, and therefore, it is difficult to differentiate mechanisms of initial allelic choice from those that reinforce allelic choice. Our findings make it likely that epigenetic changes observed at the Vκ genes in cell lines reinforce allelic decisions and do not play a role in allelic choice (Farago et al., 2012; Levin-Klein et al., 2017). Likewise, the recent observation that Vκ gene transcription is biallelic in cell lines (Levin-Klein et al., 2017) likely reflects expression in a heterogeneous cell population. Another limitation of most cell line experiments is a lack of controls relevant to normal lymphopoiesis. Whereas in F1 cell line populations, the *Igκ* alleles can be compared, the presence or magnitude of observed differences in relevant primary cells remains largely unexplored.

Cyclin-D3-mediated repression of Vκ transcription is independent of its role in cycle progression (Powers et al., 2012). Different domains of cyclin D3 mediate cell cycle progression and Vκ repression, as inhibition of CDK4/6, dampens cell cycle progression, but does not induce Vκ transcription (Powers et al., 2012). Furthermore, Vκ repression is mediated by the large fraction of cyclin D3 bound to the nuclear matrix and not available to productively bind CDK4/6. Fundamentally, we do not understand how genes translocate to transcription factories, and therefore, it is difficult to postulate how cyclin D3 might regulate this process. However, our data identify a specific mechanism regulating gene activation by transcription factories.

Our observation that elongating RNAP can track over long genomic distances is similar to the behavior of the RAG proteins, which scan for recombination signal sequences (Hu et al., 2015). Both mechanisms are constrained by CTCF-bound sites and therefore both function within TADs. Together, these two sequential loop capture events, first by NM-RNAP and then by RAG1/2 (Hu et al., 2015), are predicted to shape repertoire and lead to monogenic Vκ-Jκ recombination.

Other V genes, including *Igh* and *Tcrγ* V genes, were derepressed in *Ccnd3*$^{-/-}$ pro-B cells. This suggests that monoallelic choice at these loci might also be determined by V accessibility. More broadly, cyclin D3 predominantly repressed monoallelically expressed genes, including members of the olfactory and protocadherin gene families. Olfactory receptors are encoded by a very diverse family of receptor genes (approximately 1,400 in mice; Monahan and Lomvardas, 2015) that are expressed monoallelically and monogenically in terminally differentiated olfactory neurons. Like the Vκ genes, multiple olfactory genes can be transcribed in single immature neurons prior to terminal maturation and the choice of a single gene (Hanchate et al., 2015). Also, similar to antigen receptor genes, protocadherin and olfactory gene segments are clustered within topological domains (Guo et al., 2012; Monahan and Lomvardas, 2015). These data suggest that TAD capture transcription, and its regulation by cyclin D3, is a general mechanism of monogenic choice among clustered gene families.

EXPERIMENTAL PROCEDURES

Mice

WT (C57BL/6), *Ccnd3*$^{-/-}$ (C57BL/6), *Rag2*$^{-/-}$ (Balb/C), and C57BL/6 × CAST/EiJ mice were housed in clean animal facility at University of Chicago and used at 6–12 weeks of age under Institutional Animal Care and Use Committee (IACUC) protocol.

Isolation and Culture or Sorting of WT, *Ccnd3*$^{-/-}$ Pro-B, and *Rag2*$^{-/-}$ Pro-B Cells

Pro-B cells were isolated by positive selection (CD19$^+$) for *Rag2*$^{-/-}$ pro-B cells or by negative selection (CD3$^-$CD4$^-$CD8$^-$Ter-119$^-$IgM$^-$CD11b$^-$CD11c$^-$Gr-1$^-$NK1.1$^-$) for WT and *Ccnd3*$^{-/-}$ mice using magnetic-activated cell sorting (MACs) columns and culturing them in 12 ng/μL of IL-7 for 2 days. Alternatively, cells were fluorescence-activated cell sorting (FACS) sorted for pro-B cells (CD19$^+$B220$^+$IgM$^-$CD43$^+$) and small pre-B cells (CD19$^+$B220$^+$IgM$^-$CD43$^-$small).

In Situ Hybridization

Vκ (RP-23 182E6), Cκ (RP-24 387E13), and Vβ (RP-23 184C1) BACs (Children's Hospital Oakland Research Institute [CHORI]) were labeled using nick translation. The Vκ RNA probe (Vκ1-117) bound between heptamer recombination signal sequence (RSS) and 3′ UTR of Vκ1-117 (Table S3), and the Jκ RNA probe bound 5′ of Jκ1 (Table S3; Affymetrix). RNA probes were used with ViewRNA ISH kit (Affymetrix; QVC0001).

Combined Immuno DNA-RNA FISH

Cultured or FACS-sorted pro-B (B220$^+$CD19$^+$IgM$^-$CD43$^+$) and small pre-B (B220$^+$CD19$^+$IgM$^-$CD43$^-$) cells were plated on poly-L lysine and washed on ice with CSK buffer with 0.5% Triton (Sawasdichai et al., 2010). For DNA FISH plus IF, cells were fixed with 2% paraformaldehyde (PFA), treated with 0.2 μg/mL RNase for 30 min in 37°C followed by 0.7% Triton/0.1 M HCL for 10 min on ice, denatured in 50% formamide/2× saline sodium citrate (SSC) for 10 min in 80°C, hybridized to probes at 37°C for 16 hr, and washed, blocked, and stained the next day (Chaumeil et al., 2013). Samples were stained for pSer2 RNA Pol II (clone 3E10; EMD Millipore 04-1571) and cyclin D3 (Cell Signaling Technology; DCS22 mAb 2936) and mounted in Prolong Gold (Molecular Probe P36930). For DNA-RNA FISH, the RNase step was omitted, and after DNA-FISH and IF steps, samples were incubated at 40°C with RNA probe followed by pre-amplification mix, amplification mix, and finally labeled probe (as described by the manufacturer; ViewRNA ISH Assay Affymetrix; QVC0001).

Combined Immuno RNA FISH

For RNA FISH using distal (488-labeled) and proximal RNA probe (647-labeled) cocktails, probes were custom made by Affymetrix (Table S1). FACS-sorted CD43^{low} small pre-B cells (B220$^+$CD19$^+$IgM$^-$CD43^{low}) were plated on poly-L-lysine-coated coverslips, fixed, and permeabilized. After immuno-staining for pSer2 RNA Pol II, cells were hybridized with RNA probes using manufacturer's protocol (ViewRNA ISH Assay Affymetrix; QVC0001). Samples were mounted in Prolong Gold for imaging.

Imaging

Images were captured with a Leica TCS SP5 II STED laser scanning confocal microscope (Leica Microsystems), and image processing was performed using ImageJ. For super-resolution imaging, samples were mounted in Prolong Gold and images captured with a Leica SR GSD 3D Ground State Depletion Microscope 3 days post-mounting. Alexa Fluor 647 was depleted with 15% laser (approximately 23 mW) and acquired at threshold of 10 events with 5% laser and 10% back-pump (approximately 2 mW of 405-nm laser power). Alexa Fluor 532 was depleted with 100% laser and acquired with threshold of 20 with 20% laser.

ICeChIP

Preparation of Chromatin

Pro-B cells from *Rag2*$^{-/-}$ bone marrow were cultured in complete Opti-MEM supplemented with IL-7 (10 ng/mL). After one week, 65 million cells were isolated and nuclei prepared for ICeChIP as described (Grzybowski et al., 2015). Next, semisynthetic nucleosome standards for H3K27me3, H3K4me3, H3K9me3, H3K36me3, and H3K79me2 were "spiked in." Native chromatin (containing standards) was digested with MNase (Worthington). Chromatin was released from nuclei with 0.6 M NaCl, centrifuged, and soluble chromatin (supernatant) collected. Mononucleosomes were purified from the clarified fragmented chromatin extract using hydroxyapartite (HAP) resin (Bio-Rad Ceramic HAP type I 20 μm) and Millipore Ultrafree MC-HV Centrifugal Filters (0.45 μm).

Semisynthetic nucleosome standards were generated by refolding (at equal molar) recombinant core histones (H2A, H2B, and H4) with semisynthetic histones (H3) consisting of a recombinant H3 protein harboring the indicated synthetic histone modification. Purified histones were then mixed with barcoded ladder DNA identifiable by sequencing.

H3K27me3 ChIP was performed with 10 μg of chromatin and the remainder used as input control (Grzybowski et al., 2015). Protein A Dynabeads conjugated to H3K27me3-specific antibodies (CST C36B11) were incubated with chromatin purified and spiked as above for 10 min at 4°C. Beads were washed as described (Grzybowski et al., 2015), samples eluted, and both samples and input treated with RNase A. Proteins were digested with proteinase K and DNA recovered using a Qiaquick DNA Purification kit (QIAGEN).

Data Preparation and Analysis

Paired-end DNA libraries were prepared from input and immunoprecipitation (IP) samples followed by sequencing on an Illumina NextSeq500 by the University of Chicago Functional Genomics Core facility. Data were prepared by the following pipeline (Grzybowski et al., 2015). Raw FastQ reads were run through FastQ Grommer and then aligned with Bowtie alignment software (sensitive preset option; end-to-end alignment) to the mm9 reference genome (mm9_NCBI_build_37.1) concatenated with semisynthetic nucleosome standard barcode sequences. Reads that were unpaired, unmapped, and in wrong pair were filtered out using SAMtools. Low-quality reads (MAPing Quality [MAPQ] score < 20) were also removed. High-quality paired-end reads were flattened into single entries, and reads longer than 220 bp were removed to exclude fragments larger than mononucleosomes. Resulting files were then converted to genome coverage bedgraphs using BEDTools.

IP specificity and HMD were determined with the following equations (Grzybowski et al., 2015):

$$Barcode\ \ P\,enrichment = \frac{\sum_1^N IP}{\sum_1^N input};$$

$$HMD\ (per/bp) = \frac{\frac{IP}{input} * 100\%}{Barcode\ IP\ enrichment}.$$

Barcode enrichment corresponds to the number of barcode reads for each semisynthetic nucleosome standard in the IP compared to the input sample. This information was then used to calculate HMD. BEDtools MapBed was used to calculate the average H3K27me3 HMD over gene regions. For meta-analysis of HMD and accessibility (ATAC-seq), cushions corresponding to 10% of each gene's length were added to the beginning and end of each gene. Annotate peaks (Homer software) was then used to generate 100 bins for each gene and then calculate the average HMD for each percentage of the gene region. Accessibility data were further analyzed focusing on the region −10% to +10% corresponding to the transcriptional start site.

Single-Cell RNA-Seq of B6XCAST F1

CD19^{+}B220^{+}IgM^{-}CD43^{low} small pre-B cells were bulk sorted and then single cell sorted on pre-primed C1 Fluidic Chips. Cells were lysed, RT performed (Clontech SMARTer), and cDNAs transferred to 96-well plates. Libraries were prepared using Nextera XT (Illumina) and then tagmented. Pooled libraries were purified with AMPure magnetic separation assayed for quality with Qubit dsDNA HS (Life Technologies) and sequenced (75 bp paired). Raw fastq data were quality trimmed to a minimum Phred score of 20 using trimmomatic. Reads were then filtered against mouse ribosomal sequences using bowtie2, followed by full genome and transcriptome alignment to mouse reference mm10 using STAR. Apparent PCR duplicates and unassignable reads were removed using Picard Mark Duplicates (http://broadinstitute.github.io/picard). SNPs for CAST/EiJ mice were obtained from release-1505-GRCm38 from Sanger Institute's ftp site (ftp://ftp-mouse.sanger.ac.uk/). SNPs with quality score of at least 100, reported by Sanger, were retained for further analysis. A coverage threshold of 30 per SNP per expressed Vκ segment was used for allelic assignment.

Unrearranged cells were discriminated by biallelic expression of distal 5′Jκ1 promoter and/or proximal promoter (Jκp2), presence of heptamer and nonamer RSS motif in the J segment reads, and absence of recombination products. Read alignments were split into separate bed regions based on their CIGAR assignment for splicing (N in CIGAR strings) using bedtools bamtobed. V segments annotated using bedtools were counted to get V-V splicing. Spliced Vκ genes were interpolated and used for the analysis. Splice junction sequences were consistent with published literature (Padgett, 2012). Only segment-to-segment splicing that was observed at least 10 times was reported.

Monoallelic Gene Expression Database

Gene ids of 62 cyclin-D3-repressed genes were entered on dbMAE database (https://mae.hms.harvard.edu/) and tested for random monoallelic expression in multiple tissues. Allele-specific expression data from multiple tissues compiled from various studies were used to assess random monoallelic expression (Savova et al., 2016).

DATA AND SOFTWARE AVAILABILITY

The accession number for the scRNA-seq data reported in this paper is GEO: GSE84705.

SUPPLEMENTAL INFORMATION

Supplemental Information includes five figures and three tables and can be found with this article online at https://doi.org/10.1016/j.celrep.2018.07.091.

ACKNOWLEDGMENTS

We thank members of Clark Lab, M. Mandal, K. Mclean, M. Okoreeh, D. Kennedy, and our collaborators for critically reading the manuscript and providing helpful suggestions. Work was supported by NIH (AI120715,AI082724, and GM101090). Imaging work was done at the Integrated Microscopy Core Facility supported by Cancer Center Support Grant P30 CA014599. Single-cell sequencing data analysis was supported in part by the National Center for Advancing Translational Sciences, NIH, through grant UL1TR000050, affiliated with Center for Research Information (CRI) at University of Illinois at Chicago (UIC).

AUTHOR CONTRIBUTIONS

Imaging was done by S.K., and *in situ* hybridization was done by S.K. with technical advice from E.D. from the M.M.L.B. laboratory (University of Chicago) and the Skok laboratory (New York University). Bulk sorting for single-cell sorting was done by S.K. and H.X., and single-cell RNA-seq experiments were performed by S.K. and the Cincinnati Children Hospital Genomic core in collaboration with H.S. laboratory. ICeChIP-seq was performed by D.E.K. with help from the A.J.R. laboratory. D.E.K. and K.M. performed analysis of ATAC-seq data. Single-cell RNA-seq data analysis was performed by M.M.-C. M.R.C. oversaw the whole project and preparation of the final manuscript. Our work was based on previous work by S.E.P. and M.M., who also provided ongoing advice on the project. S.B. and A.R.D. provided assistance with modeling of *Igk* transcription. A.T.G. assisted with ICeChIP protocol. C.L. provided assistance with imaging.

DECLARATION OF INTERESTS

The authors declare no competing interests.

Received: September 25, 2017
Revised: April 25, 2018
Accepted: July 27, 2018
Published: August 28, 2018

REFERENCES

Amin, R.H., Cado, D., Nolla, H., Huang, D., Shinton, S.A., Zhou, Y., Hardy, R.R., and Schlissel, M.S. (2009). Biallelic, ubiquitous transcription from the distal germline Igkappa locus promoter during B cell development. Proc. Natl. Acad. Sci. USA *106*, 522–527.

Aoki-Ota, M., Torkamani, A., Ota, T., Schork, N., and Nemazee, D. (2012). Skewed primary Igκ repertoire and V-J joining in C57BL/6 mice: implications for recombination accessibility and receptor editing. J. Immunol. *188*, 2305–2315.

Bolland, D.J., Koohy, H., Wood, A.L., Matheson, L.S., Krueger, F., Stubbington, M.J., Baizan-Edge, A., Chovanec, P., Stubbs, B.A., Tabbada, K., et al. (2016). Two mutually exclusive local chromatin states drive efficient V(D)J recombination. Cell Rep. *15*, 2475–2487.

Chaumeil, J., Micsinai, M., and Skok, J.A. (2013). Combined immunofluorescence and DNA FISH on 3D-preserved interphase nuclei to study changes in 3D nuclear organization. J. Vis. Exp., e50087.

Chernukhin, I., Shamsuddin, S., Kang, S.Y., Bergström, R., Kwon, Y.W., Yu, W., Whitehead, J., Mukhopadhyay, R., Docquier, F., Farrar, D., et al. (2007). CTCF interacts with and recruits the largest subunit of RNA polymerase II to CTCF target sites genome-wide. Mol. Cell. Biol. *27*, 1631–1648.

Choi, N.M., and Feeney, A.J. (2014). CTCF and ncRNA regulate the three-dimensional structure of antigen receptor loci to facilitate V(D)J recombination. Front. Immunol. *5*, 49.

Clark, M.R., Mandal, M., Ochiai, K., and Singh, H. (2014). Orchestrating B cell lymphopoiesis through interplay of IL-7 receptor and pre-B cell receptor signalling. Nat. Rev. Immunol. *14*, 69–80.

Clowney, E.J., LeGros, M.A., Mosley, C.P., Clowney, F.G., Markenskoff-Papadimitriou, E.C., Myllys, M., Barnea, G., Larabell, C.A., and Lomvardas, S. (2012). Nuclear aggregation of olfactory receptor genes governs their monogenic expression. Cell *151*, 724–737.

Cooper, A.B., Sawai, C.M., Sicinska, E., Powers, S.E., Sicinski, P., Clark, M.R., and Aifantis, I. (2006). A unique function for cyclin D3 in early B cell development. Nat. Immunol. *7*, 489–497.

Farago, M., Rosenbluh, C., Tevlin, M., Fraenkel, S., Schlesinger, S., Masika, H., Gouzman, M., Teng, G., Schatz, D., Rais, Y., et al. (2012). Clonal allelic predetermination of immunoglobulin-κ rearrangement. Nature *490*, 561–565.

Grzybowski, A.T., Chen, Z., and Ruthenburg, A.J. (2015). Calibrating ChIP-seq with nucleosomal internal standards to measure histone modification density genome wide. Mol. Cell *58*, 886–899.

Guo, Y., Monahan, K., Wu, H., Gertz, J., Varley, K.E., Li, W., Myers, R.M., Maniatis, T., and Wu, Q. (2012). CTCF/cohesin-mediated DNA looping is required for protocadherin α promoter choice. Proc. Natl. Acad. Sci. USA *109*, 21081–21086.

Hanchate, N.K., Kondoh, K., Lu, Z., Kuang, D., Ye, X., Qiu, X., Pachter, L., Trapnell, C., and Buck, L.B. (2015). Single-cell transcriptomics reveals receptor transformations during olfactory neurogenesis. Science *350*, 1251–1255.

Hu, J., Zhang, Y., Zhao, L., Frock, R.L., Du, Z., Meyers, R.M., Meng, F.L., Schatz, D.G., and Alt, F.W. (2015). Chromosomal loop domains direct the recombination of antigen receptor genes. Cell *163*, 947–959.

Iborra, F.J., Pombo, A., McManus, J., Jackson, D.A., and Cook, P.R. (1996). The topology of transcription by immobilized polymerases. Exp. Cell Res. *229*, 167–173.

Levin-Klein, R., Fraenkel, S., Lichtenstein, M., Matheson, L.S., Bartok, O., Nevo, Y., Kadener, S., Corcoran, A.E., Cedar, H., and Bergman, Y. (2017). Clonally stable Vκ allelic choice instructs Igκ repertoire. Nat. Commun. *8*, 15575.

Lin, Y.C., Jhunjhunwala, S., Benner, C., Heinz, S., Welinder, E., Mansson, R., Sigvardsson, M., Hagman, J., Espinoza, C.A., Dutkowski, J., et al. (2010). A global network of transcription factors, involving E2A, EBF1 and Foxo1, that orchestrates B cell fate. Nat. Immunol. *11*, 635–643.

Lin, Y.C., Benner, C., Mansson, R., Heinz, S., Miyazaki, K., Miyazaki, M., Chandra, V., Bossen, C., Glass, C.K., and Murre, C. (2012). Global changes in the nuclear positioning of genes and intra- and interdomain genomic interactions that orchestrate B cell fate. Nat. Immunol. *13*, 1196–1204.

Mandal, M., Powers, S.E., Maienschein-Cline, M., Bartom, E.T., Hamel, K.M., Kee, B.L., Dinner, A.R., and Clark, M.R. (2011). Epigenetic repression of the Igk locus by STAT5-mediated recruitment of the histone methyltransferase Ezh2. Nat. Immunol. *12*, 1212–1220.

Martinez-Jean, C., Folch, G., and Lefranc, M.P. (2001). Nomenclature and overview of the mouse (Mus musculus and Mus sp.) immunoglobulin kappa (IGK) genes. Exp. Clin. Immunogenet. *18*, 255–279.

Monahan, K., and Lomvardas, S. (2015). Monoallelic expression of olfactory receptors. Annu. Rev. Cell Dev. Biol. *31*, 721–740.

Osborne, C.S., Chakalova, L., Brown, K.E., Carter, D., Horton, A., Debrand, E., Goyenechea, B., Mitchell, J.A., Lopes, S., Reik, W., and Fraser, P. (2004). Active genes dynamically colocalize to shared sites of ongoing transcription. Nat. Genet. *36*, 1065–1071.

Osborne, C.S., Chakalova, L., Mitchell, J.A., Horton, A., Wood, A.L., Bolland, D.J., Corcoran, A.E., and Fraser, P. (2007). Myc dynamically and preferentially relocates to a transcription factory occupied by Igh. PLoS Biol. *5*, e192.

Padgett, R.A. (2012). New connections between splicing and human disease. Trends Genet. *28*, 147–154.

Papantonis, A., and Cook, P.R. (2013). Transcription factories: genome organization and gene regulation. Chem. Rev. *113*, 8683–8705.

Park, S.K., Xiang, Y., Feng, X., and Garrard, W.T. (2014). Pronounced cohabitation of active immunoglobulin genes from three different chromosomes in transcription factories during maximal antibody synthesis. Genes Dev. *28*, 1159–1164.

Powers, S.E., Mandal, M., Matsuda, S., Miletic, A.V., Cato, M.H., Tanaka, A., Rickert, R.C., Koyasu, S., and Clark, M.R. (2012). Subnuclear cyclin D3 compartments and the coordinated regulation of proliferation and immunoglobulin variable gene repression. J. Exp. Med. *209*, 2199–2213.

Ribeiro de Almeida, C., Stadhouders, R., de Bruijn, M.J., Bergen, I.M., Thongjuea, S., Lenhard, B., van Ijcken, W., Grosveld, F., Galjart, N., Soler, E., and Hendriks, R.W. (2011). The DNA-binding protein CTCF limits proximal Vκ recombination and restricts κ enhancer interactions to the immunoglobulin κ light chain locus. Immunity *35*, 501–513.

Romanow, W.J., Langerak, A.W., Goebel, P., Wolvers-Tettero, I.L., van Dongen, J.J., Feeney, A.J., and Murre, C. (2000). E2A and EBF act in synergy with the V(D)J recombinase to generate a diverse immunoglobulin repertoire in nonlymphoid cells. Mol. Cell *5*, 343–353.

Savova, V., Patsenker, J., Vigneau, S., and Gimelbrant, A.A. (2016). dbMAE: the database of autosomal monoallelic expression. Nucleic Acids Res. *44* (*D1*), D753–D756.

Sawasdichai, A., Chen, H.T., Abdul Hamid, N., Jayaraman, P.S., and Gaston, K. (2010). In situ subcellular fractionation of adherent and non-adherent mammalian cells. J. Vis. Exp., 1958.

Schatz, D.G., and Ji, Y. (2011). Recombination centres and the orchestration of V(D)J recombination. Nat. Rev. Immunol. *11*, 251–263.

Xu, C.-R., and Feeney, A.J. (2009). The epigenetic profile of Ig genes is dynamically regulated during B cell differentiation and is modulated by pre-B cell receptor signaling. J. Immunol. *182*, 1362–1369.

Yancopoulos, G.D., and Alt, F.W. (1985). Developmentally controlled and tissue-specific expression of unrearranged VH gene segments. Cell *40*, 271–281.

Cell Reports, Volume *24*

Supplemental Information

Regulated Capture of Vκ Gene Topologically Associating Domains by Transcription Factories

Sophiya Karki, Domenick E. Kennedy, Kaitlin Mclean, Adrian T. Grzybowski, Mark Maienschein-Cline, Shiladitya Banerjee, Heping Xu, Elizabeth Davis, Malay Mandal, Christine Labno, Sarah E. Powers, Michelle M. Le Beau, Aaron R. Dinner, Harinder Singh, Alexander J. Ruthenburg, and Marcus R. Clark

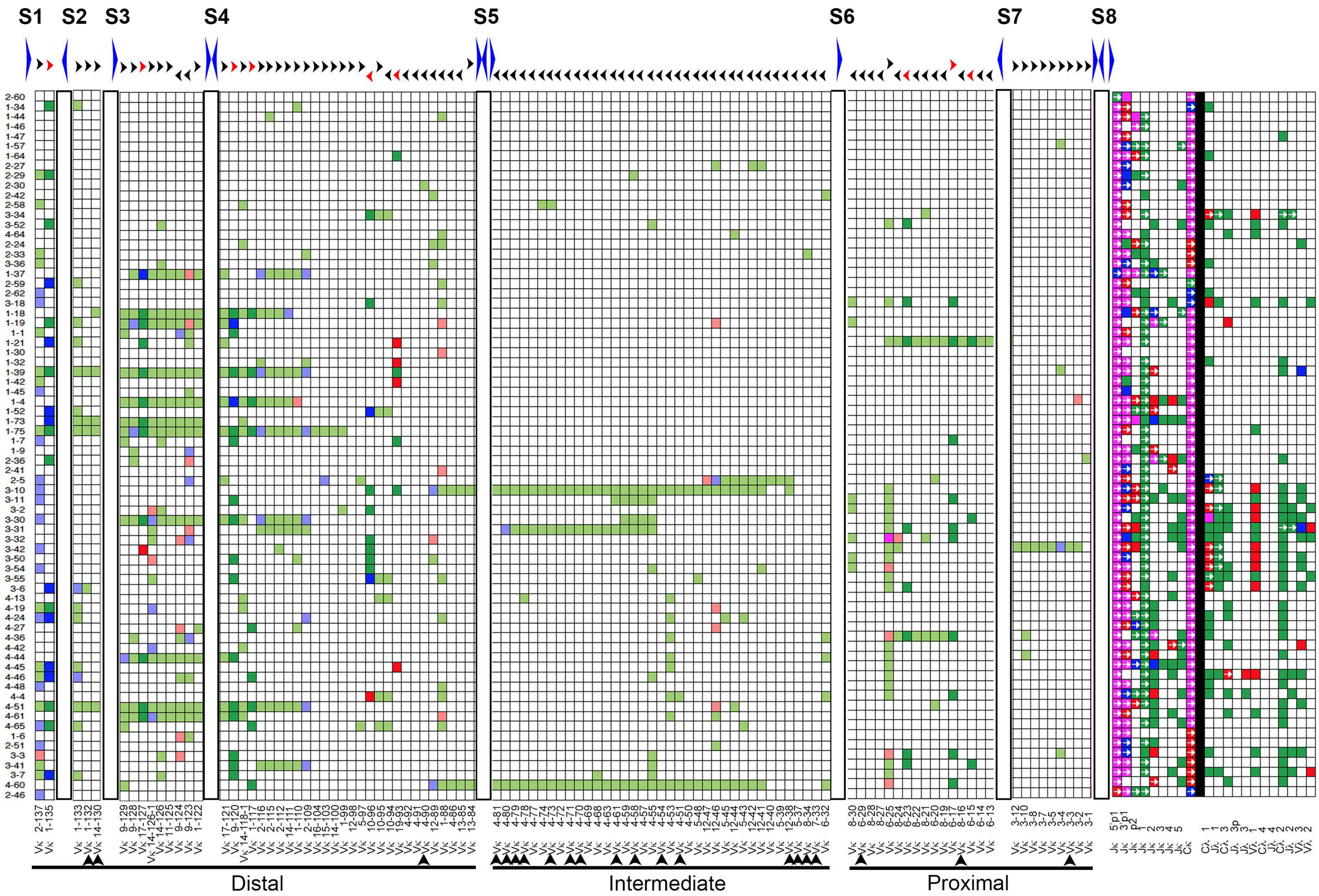

S1
S2
S3
S4
S5
S6
S7
S8
Distal
Intermediate
Proximal

Figure S1 Related to Figures 1 and 2. Single Cell RNA-seq Heatmap of *Igκ* and *Igλ* Expression.

The heatmap shows 72 analyzable cells in germline configuration, with Vκs expressed from B6 shown in red, CAST shown in blue and unassigned shown in green. High usage Vκs are shown as dark red (B6), dark blue (CAST) and dark green (unassigned). Biallelic expression of Vκ or Jκ segments is shown as magenta. All Jκ and Cκ segments are shown as dark red, blue and green for B6, CAST and unassigned respectively. Only 105 of 162 Vκ are shown, which includes 93 that have been evaluated to be functional [S1] and 12 that showed expression in 3 or more cells. Tracks of sequential Vκ expression represent those Vκs, which had undergone splicing between the first, and the last Vκ in the series and intervening Vκs were interpolated. CTCF location and orientation are shown as blue arrows on the top. Only peaks with strong binding and high contact probability (see experimental procedures) are shown [S2][S3]. Individual CTCF sites are numbered S1-S8. Smaller black arrows on the top represent transcriptional orientation of each Vκ, with red highlighting orientation of high used Vκ. Solid arrow heads at bottom denote poorly used Vκ genes.

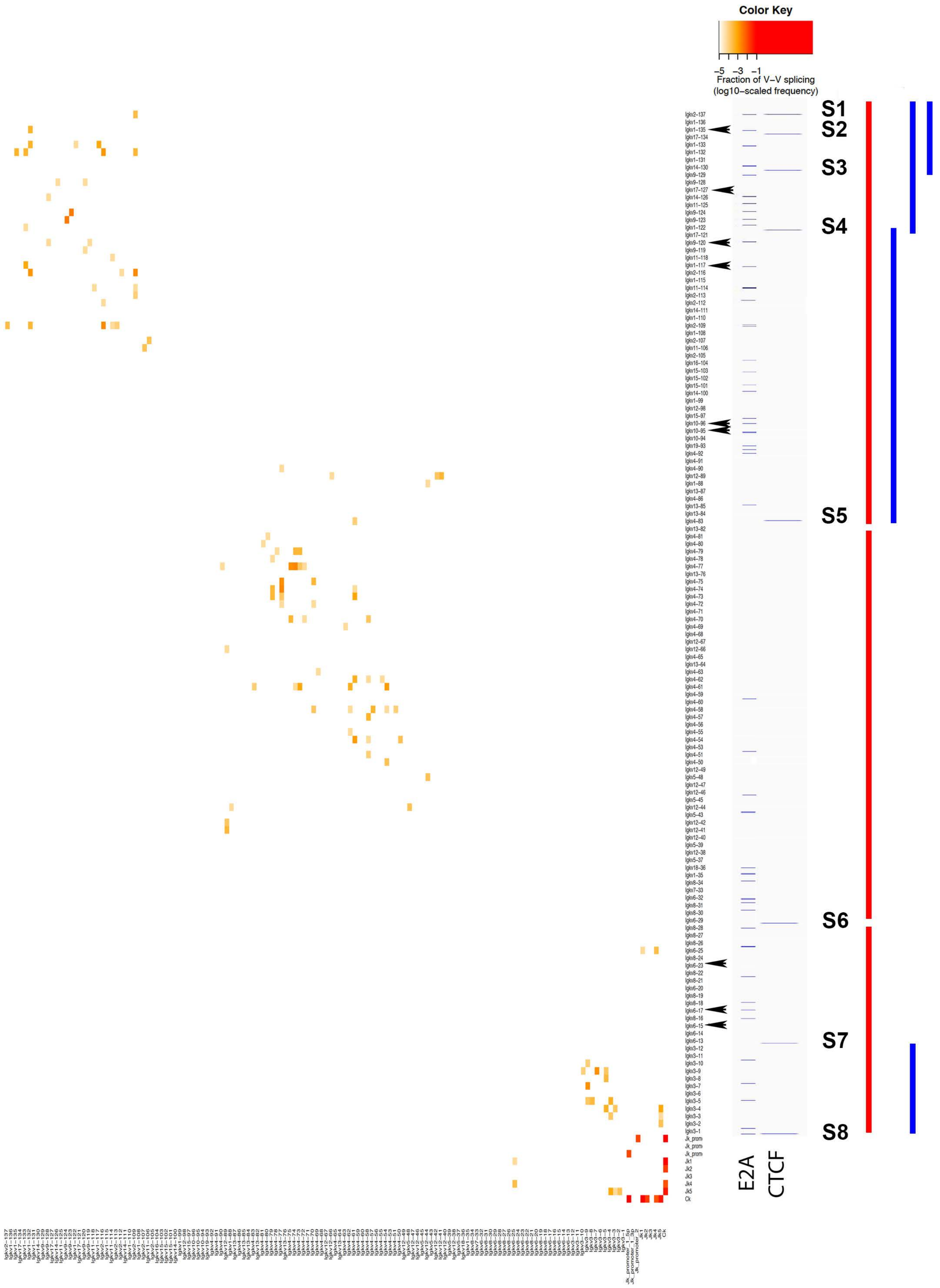

Color Key
−5 −3 −1
Fraction of V−V splicing
(log10−scaled frequency)
S1
S2
S3
S4
S5
S6
S7
S8
E2A
CTCF

Figure S2 Related to Figure 2. Heatmap of Vκ Spliced over *Igκ* Locus.

All spliced Vκs (colored) and unspliced Vκs (white space) shown. Highly used Vκs shown as black arrow. Bed files comparing CTCF peaks and E2A peaks from *Rag2*$^{-/-}$pro-B cells [S3] Individual CTCF sites are numbered as S1-S8. Red and blue lines represent TADs and sub-TADs, respectively as predicted from CTCF sites.

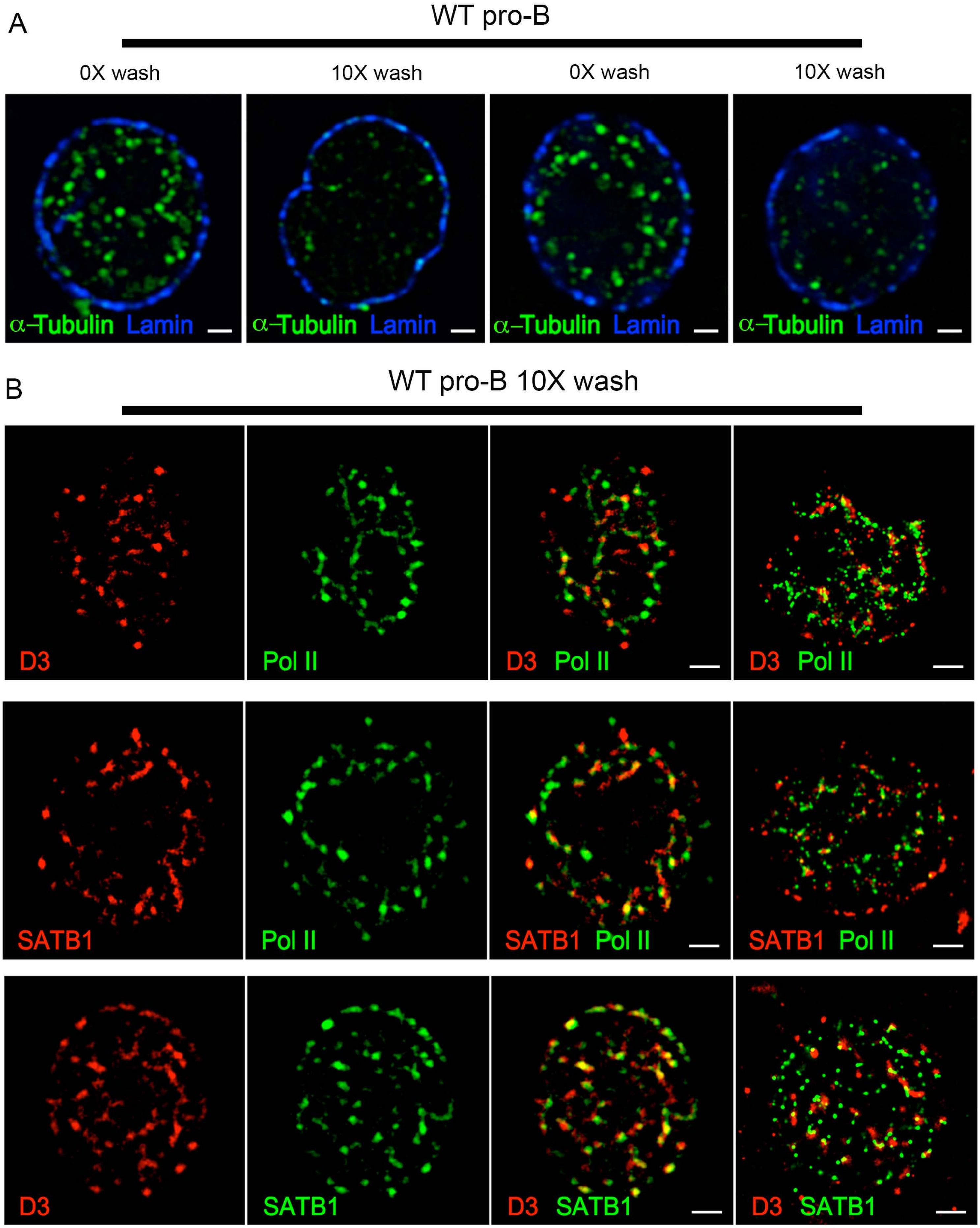
A
WT pro-B
0X wash
10X wash
0X wash
10X wash
α-Tubulin Lamin
α-Tubulin Lamin
α-Tubulin Lamin
α-Tubulin Lamin
B
WT pro-B 10X wash
D3
Pol II
D3 Pol II
D3 Pol II
SATB1
Pol II
SATB1 Pol II
SATB1 Pol II
D3
SATB1
D3 SATB1
D3 SATB1

Figure S3 Related to Figure 4. Assembly of cyclin D3 and RNAP on SATB1$^+$ Nuclear Matrix.

(**A**) Representative confocal images of WT pro-B cells (40 cells, n=2 experiments) without CSK+0.5% triton washing, fixed and stained for the soluble nuclear protein α−tubulin and lamin (panel 1 and 3). Confocal images of WT pro-B cells washed 10 times (3 minutes/wash) with CSK+0.5% triton (panel 2 and 4). Scale bar: 1μm.

(**B**) Representative confocal images of WT pro-B cells (40 cells, n=2 experiments) washed 10X (3' each) with CSK+0.5% triton buffer and fixed and stained for cyclin D3/ RNAP, SATB1/ RNAP and SATB1/cyclin D3 (panel 1-3). Super-resolution image of WT pro-B cells washed and stained with cyclin D3/ RNAP, SATB1/ RNAP and SATB1/cyclin D3 (panel 4). Scale bar: 1μm.

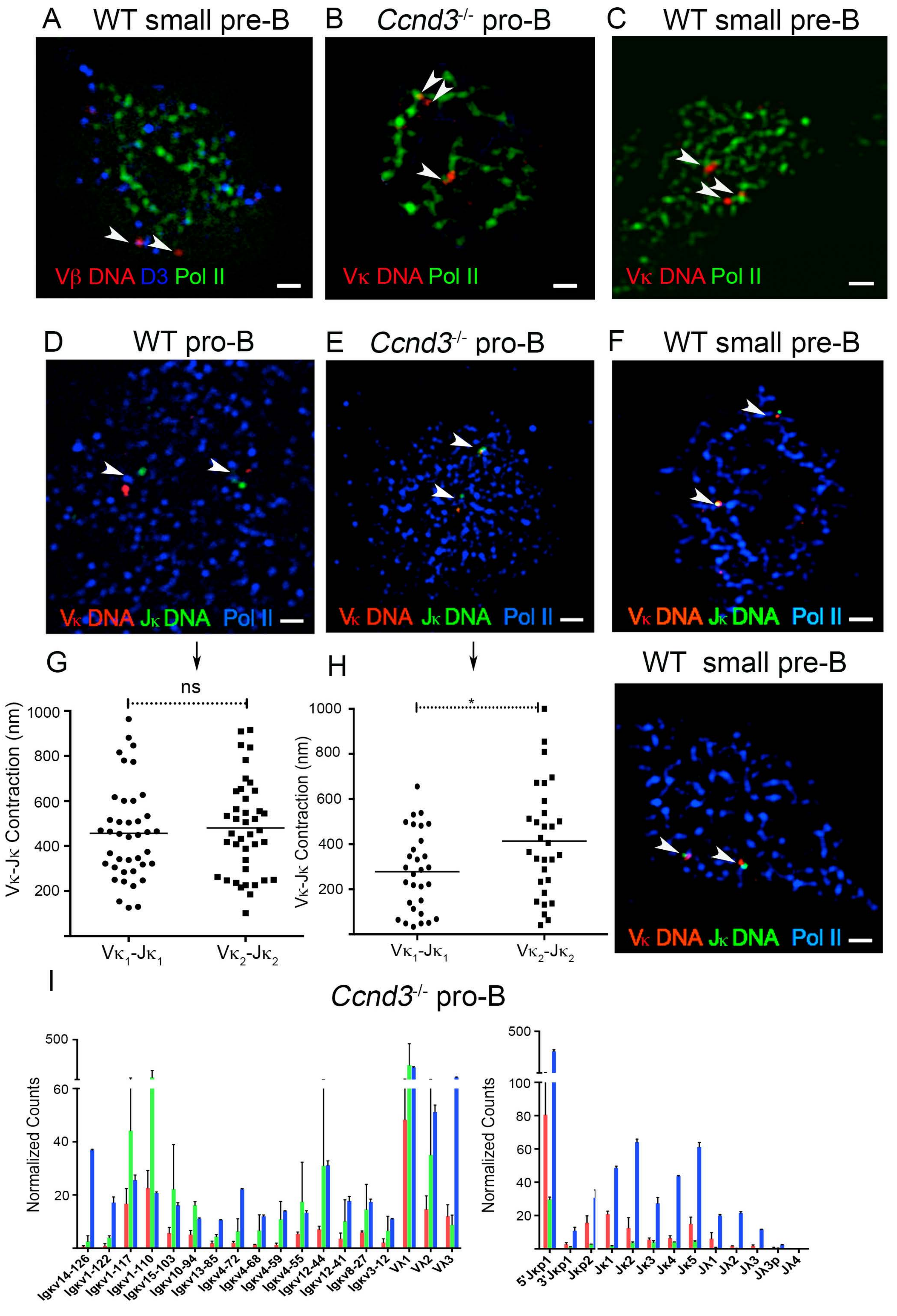

A WT small pre-B
B Ccnd3-/- pro-B
C WT small pre-B
Vβ DNA D3 Pol II
Vκ DNA Pol II
Vκ DNA Pol II
D WT pro-B
E Ccnd3-/- pro-B
F WT small pre-B
Vκ DNA Jκ DNA Pol II
Vκ DNA Jκ DNA Pol II
Vκ DNA Jκ DNA Pol II
G
ns
Vκ-Jκ Contraction (nm)
Vκ1-Jκ1
Vκ2-Jκ2
H
*
Vκ-Jκ Contraction (nm)
Vκ1-Jκ1
Vκ2-Jκ2
WT small pre-B
Vκ DNA Jκ DNA Pol II
I
Ccnd3-/- pro-B
Normalized Counts
Igkv14-126
Igkv1-122
Igkv1-117
Igkv1-110
Igkv15-103
Igkv10-94
Igkv13-85
Igkv4-72
Igkv4-68
Igkv4-59
Igkv4-55
Igkv12-44
Igkv12-41
Igkv8-27
Igkv3-12
Vλ1
Vλ2
Vλ3
Normalized Counts
5'Jκp1
3'Jκp1
Jκp2
Jκ1
Jκ2
Jκ3
Jκ4
Jκ5
Jλ1
Jλ2
Jλ3
Jλ3p
Jλ4

Figure S4 Related to Figure 5. Vκ, not TCR Vβ, Co-localizes to NM-RNAP and Transcribing Vκ Replicates Early and Contracts Preferentially.

(**A**) Representative confocal images of WT small pre-B cells (50 cells, n=3 experiments) washed with CSK+0.5% Triton buffer, hybridized with TCR Vβ DNA probe (RP23-184C1) and stained with cyclin D3 and RNAP. Arrows show individual alleles. Scale bar: 1μm.

(**B-C**) Representative confocal images of *Ccnd3*$^{-/-}$ pro-B cells (50 cells, n=3 experiments) and WT small pre-B cells and washed with CSK+0.5% Triton buffer, hybridized with Vκ DNA probe (RP23-182E6: top) spanning 10 distal Vκ genes, and stained with e-Pol II. Arrows show individual alleles. Double arrow show asynchrony in replication. Scale bar: 1μm.

(**D-F**) Representative confocal images of WT pro-B cells, WT small pre-B cells and *Ccnd3*$^{-/-}$ pro-B cells (40 cells, n=2 experiments), washed with CSK+0.5%Triton, hybridized to DNA probe RP23-182E6 (Vκ: red) and RP24-382E13 (Jκ: green) and stained for e-Pol II (blue). Arrows show individual alleles. Scale bar: 1μm.

(**G**) Minimum distances between Vκ and Jκ, plotted for alleles <200nm (Vκ1-Jκ1) from RNAP and >200nm (Vκ2-Jκ2) from RNAP using Euclidean Distance Transformation on Imaris. Analysis performed on same images used in (D). Statistical significance calculated on=37 by paired Student's t test ($p<0.05$ *, $p<0.01$ ** and $p<0.001$ ***).

(**H**) Minimum distances between Vκ and Jκ, plotted for alleles <200nm (Vκ1-Jκ1) from RNAP and >200nm (Vκ2-Jκ2) from RNAP using Euclidean Distance Transformation on Imaris. Analysis performed on same images used in (E). Statistical significance calculated on=37 by paired Student's t test ($p<0.05$ *, $p<0.01$ ** and $p<0.001$ ***).

(**I**) *Ccnd3*$^{-/-}$ pro-B RNA-seq normalized read counts, showing expression of multiple Vκ and Vλ gene segments (first panel) and repression of Jκ-Cκ and Jλ-Cλ (second panel). Red bar denotes expression in WT pro-B cells, green denotes expression in *Ccnd3*$^{-/-}$ pro-B cells and blue denotes expression in WT small pre-B cells.

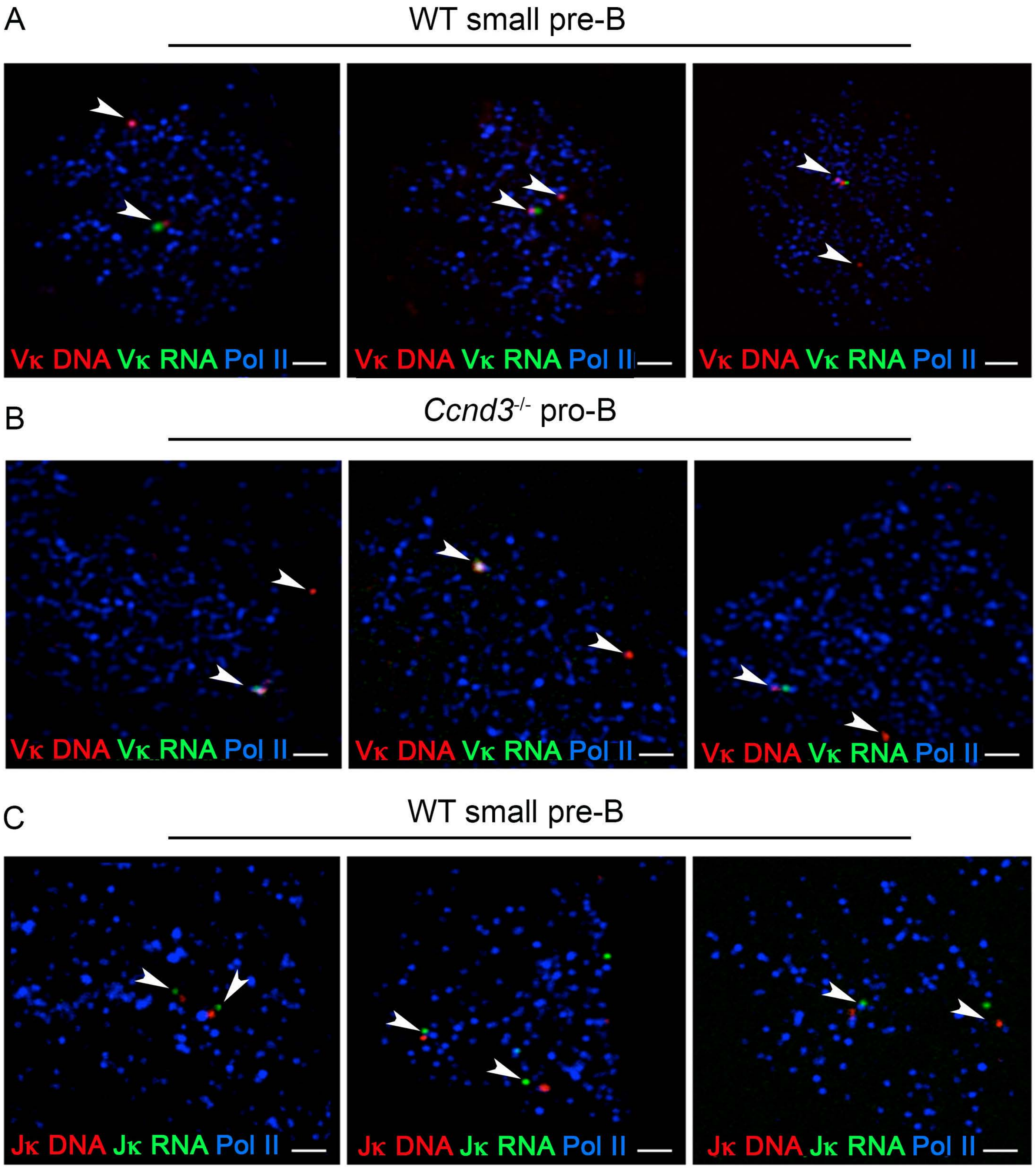

A
WT small pre-B
Vκ DNA Vκ RNA Pol II
Vκ DNA Vκ RNA Pol II
Vκ DNA Vκ RNA Pol II
B
Ccnd3-/- pro-B
Vκ DNA Vκ RNA Pol II
Vκ DNA Vκ RNA Pol II
Vκ DNA Vκ RNA Pol II
C
WT small pre-B
Jκ DNA Jκ RNA Pol II
Jκ DNA Jκ RNA Pol II
Jκ DNA Jκ RNA Pol II

Figure S5 Related to Figure 6. Mono-allelic Vκ Transcription upon Loss of cyclin D3.

(**A**) Representative confocal images of WT small pre-B cells (40 cells, n=2 experiments), washed with CSK+0.5%Triton, hybridized to Vκ DNA probe RP23-182E6 and RNA probe targeting Vκ 1-117, and stained for RNAP, shown horizontally from two independent experiments. Arrows show individual alleles. Scale bar: 1μm.

(**B**) Representative confocal images of *Ccnd3*$^{-/-}$ pro-B cells (40 cells, n=2 experiments), washed with CSK+0.5%Triton, hybridized to Vκ DNA probe RP23-182E6 and RNA probe targeting Vκ 1-117, and stained for RNAP, shown horizontally from two independent experiments. Arrows show individual alleles. Scale bar: 1μm.

(**C**) Representative confocal images of WT small pre-B cells (40 cells, n=2 experiments), washed with CSK+0.5%Triton, hybridized to Jκ DNA probe RP24-387E13 and RNA probe targeting GL Jκ and stained for RNAP, shown horizontally from two independent experiments. Arrows show individual alleles. Scale bar: 1μm.

```
>Type4
tcagcagagtggaggctgaggatttgggagtttattattgctggcaaggtacacattttcctcacacagtgattcagacc
cgaacaaaaacttccttgcctggggcagcccagctgacaatgtgcaatctggagaggagcagagagcatcttgtgtctgt
gtgagagggaggggctggggaacatgagtacttgttcacagctgtgagctctgttcagactcatatggctttatgagtgt
tttgactagatctcaagtgacatgaatttctggtccttcttgtaataggcattgtgttgcctctgcatcaggattatttc
cttttactaatctagctgatttttgatgatttaactattttattaattatttgagcttcatatttgttattcttaatga
agtatgtgtgtttgttaaattcattttcagattttaaattcttagatttgcatccatatggttaacatgcctctcacag
tgctatgtcctcttacagaaacctgcagatgctgtcagtgccgctacatcagctgcaattgaagctgttcagtgtgtgac
ctcatcaattgttggtttttattccttccatgcatatgcactctcacttcctttttcagtgtctggagcattcttggccc
cctattacttcctgaacccttcccttaagagagacagtaatagtttagatataattttccagggaagcctgtatcctgct
gcactgcagaggtactaagtcctgtccattggtacactcagttttacatttgagataaaatcaaagaatttgacttgtgc
tgaatctggtttaggtccatatataattcgttttgatattatctaatactaggaaatgagcattgtaattcaggaattat
tgatcagaagagatttaattttgtgagatgtttgtgagccatgtgtttaccaacttttgctcttaagaaactttagaaaa
atgattgctatagacttgaaaatatgcgattttattacagaaaaaaaaaaaaagcttccagaggggctgggcaaagtga
aagaggttgaaagaggaagacccagagaagagtcagaaagaattggactctcgcagatagagtggtgaaggattttgtct
caatgggttgtaagtattttgcaggggtgaattgtgtcagaataggaaggaagcaattattgttaagttggtggagacca
gtaggtaatcatttgggattaaggaagtattaggagatggtaggagatggtgaatattgagaaatcagaaagagacagtt
atgtgcacaggcccataaagcagtcagtcattttgtaaagctgtagcatgagagcggtgtggcttcatagtgactgagac
atattgacagccttggctccagggaacaatggaatctaatgaacagatagactgagtagctacatgaataagttaaacca
gccacttttaagaggtggtgcattctgtttaaaagtgagtctgaagattttgtagactattactgtctacaatatgctag
ttctcctcccacagtgatacaaatcataacataaaccccatgaaagcagaaatgagaggctgggctgctctagctgctcc
ttctcatgaatcacccactctgaatttctcagatggaacaggtctctctgagagtttggggtgactcctaagacacaaaa
tacacatatgcaatgcttctggaaggcaggagagcactacaattctgattactctaattccatacaaaagagaataataa
agttagaagatataatgccacctttctagggttccctagttatgctgtcctttgactgtctccataaattctagactgag
tttgccaactttcaaaactctttctcgaataccatttcattacactgagtttttttttcttttttttttttatcaaact
ggagatggagtttattactgctttcaaggttcacatgttcctcccacagtgatacagaccctaacaaaaatagtcttact
ttgggtatcacagctgctaaatatgttattcatgtttgagagaggttctgagggactccagtttattggttccagctgag
ggcaagtgcagcctggctacagctcactggtcattcatttttggcttcattgccacaataacaatatgaacaggtgccct
cccagaagccagaaaaaaatatttaaaaagctgagtgaaaacccatttagattcaaaaggcttgactgccttttcaatca
atcaatgttaaccagtaaagcagatggaaagccaagatatatttcttccttaaaatattttctatgttatatttcatttg
ccatattttatgttgttcactttaattcatatgtatcctttttttgaatttcaagttatagcaaaggattatggttaggcc
accttccttttctcagacaaatgtgatcaagctgttgtaagaaaaccaggaatcctatgtactgttccttttctcagtaa
tatttctctgccttttaaatagtagtgttttgtagctaattttcatcatttaaataatgttcactggaatttgcacagta
gcatccattcttctctgagtctccactgatgatacaactcaatcacttcaaaggaagcaactgcatcctttgttaaggta
gaaagggcattggtactcatgaaggggaaggaagataggtatacagagggctgtttagcccctcctaccaaaacaaatga
ctcttcatgaacttagctaaaactcctttgacatctgtgatatagtttctgttggtgactcaccatgaggaacatctgga
gaagccaaaccccacacttctgctttcatggaggtttatgttatgacttatatcattgtgggaggaagcgtattaccctg
ttggcaaaagtaagtggcaatatcttcttgctccaggttgctattattattattactctgatgatgattggtgtactaat
aaaatttcaaatcttcattgtcataataaaaaagtgtattcctcagaaccttatcacattccacatatttgttgagcaac
agtatgataaaattgtaggttattacaacattatatgagttattaaaattataaacagaaacccaaaatcatggagtgca
gtatcttgaattttatatttatatctcaatttcctttgcaaaattcaagcatatatcagtatatctaaagcagtcatttg
atatttctcttttacacatttcacaccatttcttcttactgtttagtgcatgatttttattttttaaatttattttgcaata
gcccagatttgtacatggaggtaaattcaactttattctcagaaataaaacctaaaaagaaatctgttaaaattaggccc
aaggagacttcactaatatcaattcttcatcactacaatatctaaaagcacattccccagagccttatttctttcaataa
gttcctgtagagcttatgaatgaagtgtccataagattaaagatcagagaattataatataactattgaaatattcaagt
aggactgtggatagaagtgatgactgaggcgatacaggcatggcaacacacgatcactcacatttttgttttcctgaaca
catctgaggatcacatgatcaaatgatcagatagcctttccttctagtaaagatggatttgattgcagagagcttctgag
aataacatgttcctatatatacttaattgacgtcaggggtttaaaagctggaggtagatatgtttaaattactacagtac
tcacagctctttctctttctttctctctctgtttatgtatgtgtgcccacaaagaatctctgtaatcacataactctcaa
ttgatatacttcttaaactactcaagaaaaatgatttcatacattttgatcattgaaagcatagccatatttttttatcca
gagcttttttaaaagatagatgttttttattttttttttctttttttttttcagttctctttttactaaaaggtcccaaagactt
tgcaacttagaaaaaaaaaatctcttagcaaaatggttaagacaaagggtaagaatttgtgggcaacctagcctctcactt
ctgcttccctgggtggtttttgttatgatttgtatcactgtgggtagaagattatcatactgtagacaataataagttgc
aatatcttcaggctccaggttg
```

```
>Type6
gctatttaaatagctatttactgtatcctagatttttatagattcataaaaccctgtgtgtgtgtgtactggtatgtgca
tgtgtgtgtgtgtgtgtgtgtgtgtgtgtgcatgtcagagggcacaactgtatgtcacatagaaaaaggtaaatttgttatg
ttcaatacacagaatttgagcatgagaattaaaaaaaataaagatttaatctctttacttctttttttccataaagacct
tccaaatcaaaggatcagttcgttaatttctaccaaactttgcaacacacaggcatagcacctgcaggcagaggggaag
tgtgtaggtctagggaggtgtagtgcaggcagctggttagaatctcagaggaggtttatgtaggaggctgaagcactgtg
agaggatagctgctatattggtttattactgtcagcaacattatagtactcctcccacagtgcttcagcctcctacacaa
acctccttgagagtttaaacatctgtgtacaccacacacagctctggttatgcacacttctccattcagacttagaggtg
ccatgtctgaaatgctgacgaaaaagtttacagagcccagtaaaaaataatgtgttcaatgcttcttatgtcttttggta
taaaacttttatacaccaaaatgcagggaaaaaactccacagtattgtttttctattccttctgcaacatattctgtaat
aaaaggctctgactggatttgcagctcatagtgaccttctgtcctactgacatagccagggaggatggagactgtgtcat
cacaatgtctgcacaggcacctgcaataattaataagcagtgaaaatgcatagattactgtatcctagatttttatagat
tcataaaaccctgtgtgtgtgtgtgtactggtatgtgcatgtgtgtgtatgtgtgtgtgtgtgtgtgtgtgtgtgtgtgt
gtgcatgtcagagggcacaactgtatgtcacatagaaaaaggtaaatttgttatgttcaatacacagaatctgagcatga
gaattaaaaaaaataaagatttaatctctttacttctttttttgccattaaaacattccaagtcaaaggatctattcatta
atttctaccaaactttgcaacagacaggcatagcagctgcaggcagaggggaagtgtgtaggtctagggatgtgaggtg
caggcagctggttagaatctcagaggaggtttatgtaggaggctgaagtactgtgagaggatagctgttatattg
```

Table S1 Related to Figure 1. RNA Probe Sequences of Distal and Proximal V Cocktail used for RNA ImmunoFISH.

RNA probes were custom designed by Affymetrix, targeting the distal (Vκ 1-135, 17-127, 9-120, 1-117, 10-96, 19-93) and proximal (6-23, 6-17 and 6-15) V sequences. The target sequence for each V included RSS and 3'UTR to obtain probes that would be specific for germline transcripts. Probe sequences for distal probe cocktail (Type 4: 488 labeled) and proximal probe cocktail (Type 6: 647 labeled) are shown. The target sequences have been combined and probe sequences are shown in green. Probes were hybridized using ViewRNA ISH assay and immunofluorescence was performed (see experimental procedure).

List of genes used in this study

Vκ	Activated	H3K27me3 Repressed	Ccnd3 Repressed
Igkv2-137	Btg2	Cyp27b1	MYO1B
Igkv1-135	Vpreb3	Sp6	SPAG16
Igkv1-133	Tcf3	Srcin1	CYP27A1
Igkv1-132	Btg1	Ramp2	SPP2
Igkv14-130	Ikzf1	Galr2	RAB17
Igkv9-129	Ebf1	Tmem30b	CACNA1E
Igkv9-128	Ikzf3	Dnajc22	FCGR2B
Igkv17-127	Cd79b	Ntn3	SCML4
Igkv14-126-1	Fos	H2-T3	IGF1
Igkv14-126	Irf4	Lbx1	RMST
Igkv11-125	Syk	Pitx3	DDC
Igkv9-124	Il7r	Gm996	GRB10
Igkv9-123	Ccnd3	Lhx6	BCL11A
Igkv1-122	Rag1	Hrh3	TNFSF13
Igkv17-121	B2m	AI46131	SLC16A11
Igkv9-120	Cd93	Vax2os	CCL9
Igkv14-118-1	Lef1	Zfp580	APPBP2
Igkv1-117	Bach2	Dmpk	42982
Igkv2-116	Pax5	Hmx2	DGKE
Igkv1-115	Jun	Cilp2	NME1
Igkv2-112	Actb	Comp	PDK2
Igkv14-111	Ezh2	Foxf1	FBXL20
Igkv1-110	Gapdh	Isl2	GM9866
Igkv2-109	Cd79a		SNTG2
Igkv16-104	Spib		HECTD1
Igkv15-103	Cd19		NPAS3
Igkv14-100	Irf8		KLHDC1
Igkv1-99	Hprt		5830428M24RIK
Igkv12-98			4930447C04RIK
Igkv15-97			BCL11B
Igkv10-96			GPR132
Igkv10-95			GNG4
Igkv10-94			FAM65B
Igkv19-93			JARID2
Igkv4-92			4921525O09RIK
Igkv4-91			SLC6A19
Igkv4-90			SCAMP1
Igkv12-89			CRHBP
Igkv1-88			PLK2
Igkv4-86			SYNPO2L
Igkv13-85			ANXA8
Igkv13-84			OSGEP
Igkv4-81			RAB2B
Igkv4-80			PCDH9
Igkv4-79			ZIC2
Igkv4-78			NALCN
Igkv4-77			PRLR
Igkv4-74			FBXO32
Igkv4-73			GRINA
Igkv4-72			DNAJB7
Igkv4-71			HOXC8
Igkv4-70			LEPREL1
Igkv4-69			CLDN16
Igkv4-68			SEC22A
Igkv4-63			GBE1
Igkv4-61			2810055G20RIK
Igkv4-59			CBR1
Igkv4-58			TMEM181A
Igkv4-57			THBS2
Igkv4-55			GGNBP1
Igkv4-54			4930539E08RIK
Igkv4-53			PIM1
Igkv4-51			GLO1
Igkv4-50			H2-OB
Igkv5-48			MSH5
Igkv12-47			H2-Q1
Igkv12-46			EFNA5
Igkv5-45			LBH
Igkv12-44			EHD3
Igkv5-43			CRIM1
Igkv12-42			HAAO
Igkv12-41			FZD8
Igkv12-40			DSG1A
Igkv5-39			KLHL14
Igkv12-38			LRRTM2
Igkv5-37			PCDHB16
Igkv8-34			PCDHB20
Igkv7-33			CLCF1
Igkv6-32			CAPN1
Igkv8-30			TRPM3
Igkv6-29			SCD1
Igkv8-28			TCF7L2
Igkv8-27			CACNA1B
Igkv6-25			LHX3
Igkv8-24			PDK1
Igkv6-23			YPEL4
Igkv8-22			PRG3
Igkv8-21			COMMD9
Igkv6-20			FJX1
Igkv8-19			BMF
Igkv6-17			SEMA6D
Igkv8-16			NPHP1
Igkv6-15			PRNP
Igkv6-14			DUSP15
Igkv6-13			SULF2
Igkv3-12			SPO11
Igkv3-10			ZFHX4
Igkv3-8			1700034I23RIK
Igkv3-7			PFN2
Igkv3-5			DPM3
Igkv3-4			1700094D03RIK
Igkv3-3			SEMA6C
Igkv3-2			PDZK1
Igkv3-1			DRAM2

Vκ	Activated	H3K27me3 Repressed	Ccnd3 Repressed
			AGL
			CAMK2D
			ELTD1
			ABCA1
			E130308A19RIK
			RGS3
			SLC5A9
			PTPRF
			PPT1
			MTOR
			GCKR
			MXD4
			HTRA3
			AFAP1
			SLC2A9
			KLF3
			IGJ
			2010109A12RIK
			TGFBR3
			USP30
			2410018M08RIK
			PPP1R9A
			1700025E21RIK
			RAB11FIP5
			HDAC11
			NANOG
			CCND2
			ZFP93
			CEACAM2
			SPINT2
			SBSN
			ARRDC4
			CIB1
			FURIN
			9930013L23RIK
			2310010J17RIK
			SERPINH1
			FCHSD2
			OLFR65
			COQ7
			TMC7
			TMC5
			GPRC5B
			QPRT
			IFITM1
			EPS8L2
			KCNQ1OT1
			TNFRSF22
			MYOM2
			RBPMS
			GPR97
			4632415K11RIK
			RAB4A
			1700110K17RIK
			SCN4B
			IL10RA
			SEMA7A
			CYP11A1
			LIPC
			BCL2A1D
			BCL2A1A
			BCL2A1B
			RAB6B
			CISH
			TCTA
			ARPP21
			BCL2A1C
			VILL
			1700048O20RIK
			XLR
			3830403N18RIK
			PLXNB3
			CYSLTR1
			2900056M20RIK
			TMLHE
			EIF2S3Y
			UTY
			DDX3Y

Table S2 Related to Figure 3. List of genes used in H3K27me3 ICeChIP Analysis.

Vκ regions, activated genes, and repressed genes in pro-B cells.

Vκ 1-117 RNA probe	Affymetrix Product # VB6-17816
	ggtctgtatcactgtgggaggaa<br>gatacccaaagtaagactatttttgttag<br>aacatgaataacatatttagcagctgt<br>ggagtccctcagaacctctctca<br>ccctcagctggaaccaataaact<br>ctgtagccaggctgcacttg<br>gccaaaaatgaatgaccagtgag<br>ttcatattgttattgtggcaatgaa
Jκ RNA probe	Affymetrix Product # VB6-18544
	cctgtctaaactcccacataaatactc<br>acggtctctaggctcctgacc<br>tcttttaaagtacttttatttacttctatagttc<br>cctcccctacctcctcacctct<br>ttcccctttcatactgcatatca<br>ctctgcttgtacttttagtattccact<br>ctcatctccctgccttccac<br>tggacccaccaggctacaac<br>gaatgtagagattggctgaggtcta<br>tggggactgaagaaattgaagaa<br>tgtataactactgccagtgaagcc<br>gctctgggcaaaggttggt<br>aaacacttctgtccacacgcat<br>ggctgtggaaaccaccagag<br>gcatcaaggtattccagcgag<br>taacaaggcatggagcctaact<br>aaatattttttccacgcaggaa<br>cgatctgtacataagcttattaagctaaa<br>catgcaccctagcataagagaca<br>cactggattgtacaaaaatacaactg<br>aatctacagggagctctagcagtagt<br>gctaagtagaggaattaaatatgaagatc<br>ttgtccatagacttgtaaggttattgt<br>gctagctagtttgaagatgttttgtc<br>gatcattgaggaagaaggtggtaa<br>aattgaatggccttctttggata<br>atgtaatttggcatatgtgtgctag<br>ccagttttgttccccaggtg<br>tgtgcttggttgtttttaccatt<br>ccccatgtgtctaccactgacatat<br>tgaattctgagctctctgccttc<br>ccagcttactctatacgtgtgataaac<br>ttacatattggctgttgtgggtt<br>gaggggctaagcctctgaaat<br>gtggatcagggtttactaacctca<br>cacttcacatgaaagagaaaacagtaa<br>aggacattaggctacagccattt<br>gtgaagatgtgtctcttgatttcaga<br>aagtctggtcccagtctggaaa<br>gcatgtcacaacgtggcacaa<br>cctttcctgtgtgtgcatgtgt<br>catgtgggcacaagccaagt<br>cccacttgtccctgaagaaagg<br>tgagacgctccttatgtccatt<br>caaaggaggccacgtaagga

Table S3 Related to Figure 6. RNA Probe Sequences used for Combined RNA-DNA ImmunoFISH.

RNA probes were custom designed by Affymetrix from the target sequence provided (Vκ 1-117 RSS+3'UTR and Jκ 3.5kb upstream of Jκ1). Multiple short probes spanning the region of interest were obtained. Probe sequences for Vκ1-117 and Jκ are shown. Probes were hybridized using ViewRNA ISH assay in combination with DNA FISH and immunofluorescence (see experimental procedures).

SUPPLEMENTAL REFERENCES

[S1] Martinez-Jean, C., Folch, G., and Lefranc, M.P. (2001). Nomenclature and overview of the mouse (Mus musculus and Mus sp.) immunoglobulin kappa (IGK) genes. Experimental and clinical immunogenetics *18*, 255-279.

[S2] Choi, N.M., and Feeney, A.J. (2014). CTCF and ncRNA Regulate the Three-Dimensional Structure of Antigen Receptor Loci to Facilitate V(D)J Recombination. Front Immunol *5*, 49.

[S3] Lin, Y.C., Benner, C., Mansson, R., Heinz, S., Miyazaki, K., Miyazaki, M., Chandra, V., Bossen, C., Glass, C.K., and Murre, C. (2012). Global changes in the nuclear positioning of genes and intra- and interdomain genomic interactions that orchestrate B cell fate. Nat Immunol *13*, 1196-1205.
